# Prioritizing quantitative trait loci for root system architecture in tetraploid wheat

**DOI:** 10.1093/jxb/erw039

**Published:** 2016-02-13

**Authors:** Marco Maccaferri, Walid El-Feki, Ghasemali Nazemi, Silvio Salvi, Maria Angela Canè, Maria Chiara Colalongo, Sandra Stefanelli, Roberto Tuberosa

**Affiliations:** ^1^Department of Agricultural Sciences (DipSA), University of Bologna, 40127 Bologna, Italy; ^2^Department of Crop Sciences, Faculty of Agriculture, Alexandria University, 23714 Alexandria, Egypt; ^3^Department of Agriculture, Hajiabad Branch, Islamic Azad University, 21100 Hajiabad, Iran

**Keywords:** Association mapping, drought stress, germplasm collection, GWAS, grain yield, meta-QTLs, root growth angle, rooting depth, root system architecture, seminal root.

## Abstract

Optimization of root system architecture (RSA) traits is an important objective for modern wheat breeding. Linkage and association mapping for RSA in two recombinant inbred line populations and one association mapping panel of 183 elite durum wheat (*Triticum turgidum* L. var. *durum* Desf.) accessions evaluated as seedlings grown on filter paper/polycarbonate screening plates revealed 20 clusters of quantitative trait loci (QTLs) for root length and number, as well as 30 QTLs for root growth angle (RGA). Divergent RGA phenotypes observed by seminal root screening were validated by root phenotyping of field-grown adult plants. QTLs were mapped on a high-density tetraploid consensus map based on transcript-associated Illumina 90K single nucleotide polymorphisms (SNPs) developed for bread and durum wheat, thus allowing for an accurate cross-referencing of RSA QTLs between durum and bread wheat. Among the main QTL clusters for root length and number highlighted in this study, 15 overlapped with QTLs for multiple RSA traits reported in bread wheat, while out of 30 QTLs for RGA, only six showed co-location with previously reported QTLs in wheat. Based on their relative additive effects/significance, allelic distribution in the association mapping panel, and co-location with QTLs for grain weight and grain yield, the RSA QTLs have been prioritized in terms of breeding value. Three major QTL clusters for root length and number (*RSA_QTL_cluster_5#*, *RSA_QTL_cluster_6#*, and *RSA_QTL_cluster_12#*) and nine RGA QTL clusters (*QRGA.ubo-2A.1*, *QRGA.ubo-2A.3*, *QRGA.ubo-2B.2/2B.3*, *QRGA.ubo-4B.4*, *QRGA.ubo-6A.1*, *QRGA.ubo-6A.2*, *QRGA.ubo-7A.1*, *QRGA.ubo-7A.2*, and *QRGA.ubo-7B*) appear particularly valuable for further characterization towards a possible implementation of breeding applications in marker-assisted selection and/or cloning of the causal genes underlying the QTLs.

## Highlight

The genetic variation of root system architecture in the A and B wheat genomes is described, providing the necessary knowledge ultimately to fine-tune the expression of the root system architecture.

## Introduction

Root system architecture (RSA) plays a pivotal role in crop performance, particularly for cultivation under non-optimal water and nutritional supply conditions (Ludlow and Muchow, 1990; de Dorlodot *et al.*, 2007; Paez-Garcia *et al.*, 2015). Based on the expected climate changes and the declining availability of water and fertilizers, enhancing the genetic capacity of the plant to acquire soil resources is a primary target to increase crop productivity and yield stability (Hawkesford *et al.*, 2014; Mickelbart *et al.*, 2015). In the past decade, RSA has received increasing attention in cereals (Hochholdinger and Tuberosa, 2009; Wasson *et al.* 2012, 2014; Bishopp and Lynch, 2015), leading to the development of detailed RSA ideotypes (King *et al.*, 2003; Lynch, 2013; Meister *et al.*, 2014). In rice, a narrow and deep root ideotype for enhancing drought resistance has been successfully pursued based on direct field observation of root distribution (Steele *et al.*, 2013; Uga *et al.*, 2013) or root growth angle (RGA) measurements in rhizotrons (Kitomi *et al.*, 2015). In sorghum, stay-green genotypes have contributed additional evidence for the positive role on yield of narrow RGA quantitative trait loci (QTLs) under drought conditions (Borrell *et al.*, 2014). RGA is also of paramount importance for the acquisition of phosphorus, a low-mobility nutrient usually more abundant in the upper soil layer (Miguel *et al.*, 2015).

Among cereals, wheat is prevalently grown under rainfed conditions in regions where drought stress is the major environmental factor limiting productivity. Accordingly, in at least 60 million rainfed hectares, grain yield of wheat was only 10–50% of that reached under irrigation (Fleury *et al.*, 2010; Langridge and Reynolds, 2015). Drought can affect wheat at all vegetative stages, mainly from flowering to grain filling in Mediterranean environments. Breeding for enhanced water and nutrient uptake would therefore result in increased yield and yield stability, particularly under water-limited environments (Manschadi *et al.*, 2010; Wasson *et al.*, 2012; Christopher *et al.*, 2013; Lopes *et al.*, 2014). Optimizing the anatomy and growth features of roots can significantly increase water-use efficiency (WUE; Richards and Passioura, 1989; Wasson *et al.*, 2012) and/or moisture extraction from deep soil layers (Blum, 2009; Uga *et al.*, 2013; Pinto and Reynolds, 2015). In wheat, lack of information on the effects of RSA QTLs on yield across water regimes has so far hindered the adoption of marker-assisted selection for tailoring RSA, unlike in rice (Steele *et al.*, 2013; Arai-Sanoh *et al.*, 2014) and sorghum (Borrell *et al.*, 2014).

Wheat shows two main root systems, namely the seminal (embryonal) roots and the nodal (crown or adventitious) roots (Chochois *et al.*, 2015). Seminal roots in cultivated wheat include one primary root, two pairs of symmetric roots, and, at times, a sixth central root. Nodal roots usually become visible when the fourth leaf emerges at the tillering stage (Esau, 1965). Seminal roots penetrate the soil earlier and more deeply than nodal roots and remain functional for the entire plant cycle, hence contributing to moisture extraction from deeper soil layers (Manschadi *et al.*, 2013).

Direct measurements of root length density across soil profiles showed that wheat growth on residual moisture greatly depends on roots that reach deep soil layers (Reynolds *et al.*, 2007; Acuna *et al.*, 2012; Hamada *et al.*, 2012; Manschadi *et al.*, 2013). Importantly, rooting depth has been related to the RGA of seminal roots as first reported by Oyanagi *et al.* (1993). More recently, this concept has been adopted for breeding purposes in rice (Uga *et al.*, 2015).

RGA is easily measured on seminal roots of seedlings (Sanguineti *et al.*, 2007; Richard *et al.*, 2015) or on the adventitious roots at the adult stage (Oyanagi *et al.*, 1993; Kato *et al.*, 2006). While the latter appears more relevant for crop performance, direct field-based RSA phenotyping of adult plants remains a labour-demanding undertaking, especially when experimental uniformity is required and experiments involve a large number of accessions (Mace *et al.*, 2012; Tuberosa, 2012). Conversely, RSA characterization at the seminal stage allows for an accurate, fast, and cheap evaluation of hundreds of accessions (Mace *et al.*, 2012). This notwithstanding, limited information is available on QTLs for seminal RSA in wheat (Canè *et al.*, 2014).

QTLs for RSA traits have been reported for diverse cereals (Tuberosa *et al.*, 2003; Hund *et al.*, 2011; Mace *et al.*, 2012; Christopher *et al.*, 2013; Courtois *et al.*, 2013; Acuna *et al.*, 2014; Borrell *et al.*, 2014). In rice, the identification of major QTLs for root depth has been instrumental in increasing adaptation to low water availability (Steele *et al.*, 2013) as well as for the positional cloning of *deeper rooting 1* (*DRO1*; Uga *et al.*, 2013).

Dissecting the genetic control of RSA traits is particularly important in tetraploid durum wheat (*Triticum turgidum* L. var. *durum* Desf.), a crop mainly grown under rainfed conditions and low water availability. In this study, linkage and association mapping were both used to (i) generate a comprehensive view of the QTLome (as defined in Salvi and Tuberosa, 2015) governing RSA traits in elite durum wheat at the seedling stage and (ii) investigate how these QTLs influence yield and overlap with similar QTLs in hexaploid wheat. A high-density tetraploid consensus map facilitated cross-referencing of QTLs from diverse materials and studies, hence allowing us to prioritize RSA QTLs for further studies. The genetic variation for RSA in the A and B wheat genomes is described, providing the necessary knowledge ultimately to fine-tune the expression of the RSA and model its expression based on genetic information.

## Materials and methods

### Plant material

Two recombinant inbred line (RIL) F_6:7_ populations were developed by Produttori Sementi Bologna (Bologna, Italy) from Colosseo×Lloyd (Co×Ld; 176 RILs) and Meridiano×Claudio (Mr×Cl; 181 RILs).

Colosseo is an Italian cultivar released in 1990 with a high yield potential but poorly adaptated to Southern Mediterranean environments under high terminal drought and heat (as from the Italian National Network Trial annual reports, 1995–2005). Pedigree information indicates a direct origin from Creso (one of the founders of the modern germplasm, obtained from Italian and CIMMYT early Green Revolution materials). However, microsatellite data showed that Colosseo has chromosome segments directly introgressed from Mediterranean landraces (Maccaferri *et al.*, 2007). Lloyd (Cando/Edmore) is a US cultivar well adapted to the relatively low-input conditions typical of the USA Northern Plains.

Meridiano is a medium to early maturing, widely adapted Italian cultivar derived from a complex cross between Italian, CIMMYT, and Southwestern US materials (Simeto/WB881//Duilio/F21). Claudio, another Italian cultivar (CIMMYT selection/Durango//ISI938/Grazia), is renowned for its high yield stability across drought- and heat-stressed environments of Southern Europe (Spain, Italy, and Greece). More details are reported in Maccaferri *et al.* (2005, 2011) and De Vita *et al.* (2010).

The association panel, hereafter referred to as Unibo-DP (standing for ‘UniBO Durum Panel’), includes 183 elite cultivars and lines from Mediterranean countries, the Southwestern USA, and Mexico (Maccaferri *et al.*, 2011). Based on the characterization of simple sequence repeat (SSR) markers, the population structure of the Unibo-DP accessions herein considered appeared to be structured into five main subgroups representing the main breeding lineages present in the germplasm, identified by well-defined breeding ideotypes (and corresponding hallmark founders developed in different decades of breeding). Briefly, these subgroups correspond to: S1, ICARDA and Italian accessions for dryland areas from the native Syrian and North African germplasm (from Haurani and related landraces); S2, ICARDA accessions bred for temperate areas (from Cham 1); S3, Italian cultivars related to Valnova and Creso founders and subsequently bred with CIMMYT and Southwestern US accessions (Desert Durum^®^); S4, widely adapted early CIMMYT germplasm introduced to several Mediterranean countries (from Yavaros 79, Karim, Duilio); S5, more recent high yield potential CIMMYT germplasm (from Altar84). Details are reported in Maccaferri *et al.* (2011) and in Letta *et al.* (2013). Parental line Colosseo is grouped in S3 cluster, which harbours 38 accessions, 10 of which are derivatives of Creso, including Colosseo. Parental line Lloyd genome, belonging to the Northern Plains US germplasm, is not well represented in the Unibo-DP, except for the Southwestern US germplasm and recent Italian and French cultivars which inherited several portions of the genome from the US germplasm. Parental line Meridiano appeared mostly related to the S4 group, together with 60 other accessions from ICARDA, CIMMYT, and Mediterranean countries all related to the founder cultivars Yavaros C79, Karim, and Duilio (the same germplasm released at the CIMMYT and introduced in Tunisia and Italy). Parental line Claudio is a more diversified accession which loosely clusters with the CIMMYT-derived germplasm S4 and S5. More details are given in Maccaferri *et al.* (2011).

### Phenotypic analysis

#### Evaluation at the seedling stage

The mapping populations were characterized for RSA traits at the seedling stage using the protocol reported in Canè *et al.* (2014). Briefly, seeds were manually selected for uniformity, weighed, sterilized in a 1% sodium hypochlorite solution for 10′, rinsed in distilled water, and germinated in Petri dishes at 28 °C for 24h.

Homogeneously sprouting seedlings were then grown in moist filter paper sheets in vertical black polycarbonate screening plates (42.5×38.5cm). Seedlings were grown for 9 d at 22 °C (day)/18 °C (night) under a 16h light photoperiod and light intensity of 400 µmol m^–2^ s^–1^ photosynthetically active radiation (PAR). The weight of the seeds used in the experiment, hereafter reported as ‘initial thousand grain weight’ (iTGW), was subjected to QTL analysis together with the RSA traits.

The experiments were conducted according to a randomized complete block design for each of the two populations and the Unibo-DP, with three independent replications (=experiments) grown consecutively in the same growth chamber. The experimental unit was equal to a 60cm wide screening plate allotting eight seedlings of the same genotype, so that one screening plate corresponded to one genotype. To avoid border effects, root seminal architecture traits were measured from the six central seedlings only. Due to the high number of genotypes to be evaluated and considering the time needed for root preparation and acquisition of photographs of the roots, blocking was introduced to control for possible differences in growth rate. Twenty-five to 30 accessions were included in one block, corresponding to six blocks for each of the two populations and the Unibo-DP. Blocks corresponded to shelves in the growth chamber, positioned at the same distance from the floor under uniform light conditions.

Blocking was taken into account in the ANOVA, and linear adjustment for block effect (normalization) was carried out as necessary.

**Fig. 1.**
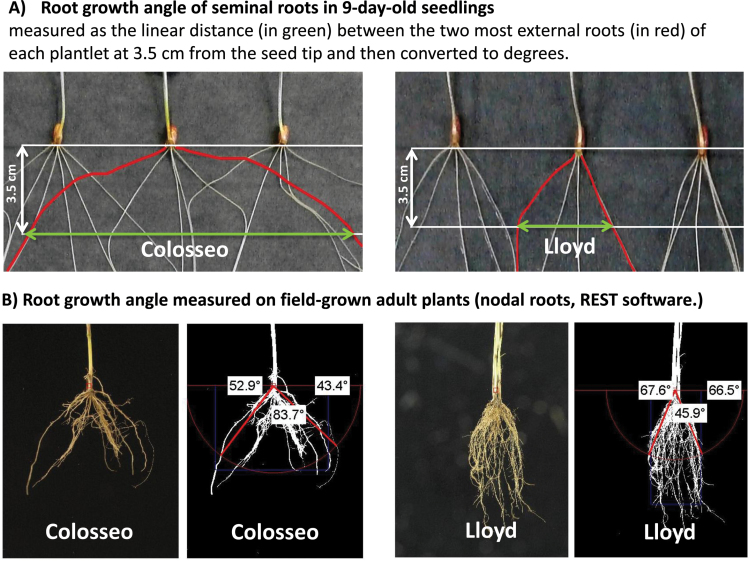
Measurement of root growth angle (RGA) in seminal roots at the seedling stage and in excavated roots of field-grown plants at the end of flowering. The two parental genotypes Colosseo and Lloyd, highly contrasted for RGA at both stages, are shown as an example. Direct measurements were carried out for seminal root RGA, while software-aided digital measurements were carried out for adult root systems (REST software). (This figure is available in colour at *JXB* online.)

Root seminal architecture traits were measured on a single-plant basis: RGA (see Fig. 1A for an example) was obtained as the linear distance between the two most external roots of each plantlet at 3.5cm from the seed tip and then converted to degrees; total number of roots (TRN); presence of the sixth seminal root (Rt6); and shoot length (SL). Additional RSA traits such as single root length, surface, diameter, and volume (PRL, PRS, PRD, and PRV, respectively) were measured on images using a digital camera with a shutter time set at 100.

Images were processed using SmartRoot^®^ (Lobet *et al.*, 2011). Data from the primary roots were considered separately from those of the other roots. Lateral roots were counted on the primary root using a magnifying lens. After measuring SL, shoots and roots were cut and dried to determine the total dry weight for each replicate (SDW and RDW, respectively).

The root seminal architecture data for the DurumPanel accessions are reported in the DArT- and SSR-based association study by Canè *et al.* (2014). The same data were re-analyzed for a more accurate single nucleotide polymorphism (SNP)-based genome-wide association study (GWAS).

#### Field evaluation of RGA

On 22 October 2014, the Unibo-DP was planted in Cadriano, Po Valley, Italy (44°33'N and 11°27'E) under a complete randomized block design with two replicates and optimal agronomic management. The soil is a highly fertile agricultural loam (Udic Ustochrepts, fine silty, mixed, mesic). Accessions were grown in 3-m-long twin rows (20cm apart) with plants spaced apart by 10cm, for a final density of 200 plants m^−2^. Forty accessions were selected for root phenotyping in the field as follows: (i) two sets of 12 accessions each were chosen from the top- and bottom-ranking lists of the 183 accessions sorted for seminal RGA (Canè *et al.*, 2014) and (ii) an additional 16 accessions used as parents of mapping populations developed at UNIBO, plus Colosseo, Lloyd, Meridiano, and Claudio parental lines were considered. The relationship between RGA at the seedling stage and the mature RGA phenotypes of field-grown plants at the post-flowering stage was investigated by using a ‘shovelomics’ approach (Trachsel *et al.*, 2011). Root phenotyping in the field was carried out at the end of the flowering stage (Zadocks 69). In the selected plots, a 40cm long and 30cm wide central section was chosen for a 30cm deep core-root system excavation. Root soil cores of 16–20 plants were water-soaked overnight, rinsed in clean water and allowed to dry. From each plot, the eight undamaged plants most homogeneous in tiller number along the row plot were imaged one by one (see Fig. 1B for an example). Images were processed with the Rest software (http://www.plant-image-analysis.org/software/rest).

### Molecular data and genetic map construction

The SSR and DArT^®^ profiles of the Co×Ld and Mr×Cd RIL populations, their parents, and the Unibo-DP accessions (Maccaferri *et al.*, 2008, 2011; Mantovani *et al.*, 2008) were integrated with the high-density Infinium^®^ iSelect^®^ Illumina 90K SNP array (Wang *et al.*, 2014; Maccaferri *et al.*, 2015). DNA was extracted from a bulk of 25 one-week-old seedlings per accession using the DNeasy 96 Plant Kit (Qiagen GmbH, Hilden, Germany). The final array included 81 587 transcript-associated SNPs, 8000 of which are durum-specific SNPs (Wang *et al.*, 2014). Genotyping was performed by TraitGenetics GmbH (Gaterlesleben, Germany), including duplicates of parental lines and reference cultivars. Briefly, the Infinium^®^ iSelect^®^ Illumina 90K SNP assay was performed on the 24 sample HD BeadChip format where genomic DNA is isothermally amplified, fragmented, and hybridized to the BeadChip. The amplified and fragmented DNA samples anneal to locus-specific, SNP-specific oligomers that are immobilized on the beads, then single-base extension at the SNP position is carried out on the BeadChip using the captured DNA as template and incorporating detectable and spectrally distinct fluorescent dyes associated with the two alternative alleles. The iScan^®^ system laser-excites and laser-scans the BeadChip array at ultra-high resolution, thus providing the light-emitted raw quantitative output for each bead. The automated process is carried out according to the manufacturer’s protocols (Illumina, Inc., San Diego, CA, USA). SNP clustering and genotype calling were performed using GenomeStudio v2011.1 software (Illumina, Inc.). Since the GenomeStudio v2011.1 was developed for diploids, a specific cluster file was developed by manually adjustment of the Illumina Genome Studio cluster calling for each marker to capture variation in data correctly. The cluster file is available upon request from Martin Ganal (TraitGenetics GmbH, Gatersleben, Germany). For the two mapping populations, linkage maps were based on the joint analysis of SSR, DArT^®^, and SNP data from the Illumina 90K SNP assay that were assembled using a common mapping methodology described in Maccaferri *et al.* (2015). Briefly, the common mapping procedure involved the use of stringent thresholds for marker and line quality data filtering and the use of Carthagene v1.2.3 for gouping and mapping. The two genetic maps used for QTL analysis are reported as supplemental material in Maccaferri *et al.* (2015).

A tetraploid consensus map incorporating SSR, DArT^®^, and SNP markers from 13 mapping populations (Maccaferri *et al.*, 2015) has been used to compare QTL results across mapping populations and the durum association panel and with those reported in bread wheat. The consensus map includes SSR, DArT^®^, and SNP markers for a total of 30 244 mapped markers and 2631 cM (11.5 markers cM^–1^, on average).

### Statistical analysis

ANOVA (general linear model, unbalanced ANOVA) was conducted for all RSA traits including blocks, replicates, and genotypes. Block effect, when significant, was accounted for by linear regression correction. The weight of the same seeds used in the RSA experiments (iTGW) was used as a covariate to correct for variation in seed weight in the root experiments. In addition, iTGW was also subjected to QTL analysis, similarly to the other root traits analysed.

ANOVA was carried out in Minitab^®^15, Minitab Ltd, Coventry, UK.

Heritability (*h* ^2^) was calculated on a mean basis across three replications according to the formula:

$$h^{2}=\sigma_{\text{G}}{}^{2}\left(\sigma_{\text{G}}{}^{2}+\sigma_{\text{E}}{}^{2}/r\right)$$

where: σ_G_^2^=genetic variance, σ_E_^2^=residual variance, *r*=number of reps, σ_G_^2^=(MS_genotypes_–MS_residual_)/*r*, σ_E_^2^=MS_residual_, and MS stands for mean square value.

The coefficient of variation (CV) was defined as the SD (standard deviation)/mean: CV=σ/µ; it assesses the extent of residual variability in relation to the phenotypic mean.

QTL analysis was carried out based on single marker analysis and multiple interval mapping (MIM; Kao *et al.*, 1999) in Windows QTL Cartographer v2.5 (http://statgen.ncsu.edu/qtlcart/WQTLCart.htm). Details on the MIM QTL searching model are given in Supplementary Text S1 at *JXB* online.

For each QTL, the effect was computed as the phenotypic difference between the mean values of the RIL groups homozygous for the two parental alleles at the QTL peak position.

As a general rule, two QTLs identified for different root traits were considered as a single QTL cluster whenever their confidence intervals overlapped, QTL peaks mapped within a 15 cM interval, and the direction of the QTL effects showed consistency across traits.

In the Unibo-DP, a total of 19 815 bi-allelic SNP and DArT^®^ markers were mapped to unique positions. Details on the GWAS model adopted are given in Supplementary Text S2. Briefly, three levels of significance were considered for reporting the GWAS QTLs: (i) marker-wise *P*≤0.01 (–log_10_ *P*≥2.00) for suggestive QTLs; (ii) marker-wise *P*≤0.001 (–log_10_ *P*≥3.00) for nominal QTLs; and (iii) experiment-wise *P*≤0.05 (–log_10_ *P*≥4.00) for major QTLs.

Due to the relatively high density of informative SNPs mapped from the panel (13 823 mapped SNPs with minor allele frequency ≥0.05, corresponding to ~5.32 SNPs cM^–1^) compared with the linkage disequilibrium (LD) decay rate observed in the panel (decay to *r* ^2^=0.3 at 2.20 cM distance, on average), the experiment-wise GWAS significance threshold was set according to the actual number of ‘independent SNP tests’. The latter was estimated in Haploview using the tagger function using an *r* ^2^ tag threshold of 0.3 (Mackay, 1996). The total number of tag-SNPs was equal to 773, rounded to 1000, hence the experiment-wise, Bonferroni-corrected significance threshold at *P*=0.05 corresponded to a marker-wise threshold of –log_10_ *P*≥4.00.

Since the observed density of mapped informative SNPs in the Unibo-DP (5.32 SNP/cM) far exceeded the minimum SNP density required to find QTL associations in the panel (based on the average genetic distance for LD decay to *r* ^2^=0.3: 2.2 cM), GWAS QTLs were most frequently detected as multiple marker–trait associations of SNPs in LD with each other. For each QTL, the most associated SNP was considered as the QTL-tagging SNP marker.

The relative positions of RSA QTLs identified in our mapping populations, Unibo-DP, and in previous linkage studies in wheat (Laperche *et al.*, 2006; Kubo *et al.*, 2007; Sanguineti *et al.*, 2007; Guo *et al.*, 2012; Hamada *et al.*, 2012; Ren *et al.*, 2012; Bai *et al.*, 2013; Christopher *et al.*, 2013; Liu *et al.*, 2013; Cao *et al.*, 2014; Atkinson *et al.*, 2015; Petrarulo *et al.*, 2015) were compared based on the projected QTL peaks and confidence intervals on the tetraploid consensus map used as a common reference (Maccaferri *et al.*, 2015). Marker and QTL projection was carried out in Biomercator version 4.2, using QTLProj subroutine (BioMercator V4; Sosnoswski *et al.*, 2012).

### Co-location of RSA QTLs with QTLs for grain weight and grain yield

The Unibo-DP was field-assessed in 15 field experiments carried out in the Mediterranean region in 2004 and 2005. The thousand grain weight (TGW) and grain yield (GY) data used were those published in Maccaferri *et al.* (2011). Trials were classified into three categories of (i) three low-yielding trials with yield from 0.9 t ha^−1^ to 2.9 t ha^−1^); (ii) five medium-yielding trials with yield from 3.5 t ha^−1^ to 4.6 t ha^−1^); and (iii) seven high-yielding trials with yield from 5.4 t ha^−1^ to 6.7 t ha^−1^. TGW and GY data have been reanalysed for GWAS based on the new SNPs. All GWAS QTLs are presented as a unique QTL density plot for each of the three trial categories using 1 cM as plot unit and the QTL significance intervals.

## Results

### Phenotypic variation in Colosseo×Lloyd and Meridiano×Claudio for RSA

Co×Ld and Mr×Cl RIL populations were assessed for seminal RSA traits at the seedling stage. A summary of the phenotypic values for root and shoot traits is reported in Table 2 and distribution frequencies are reported as histograms in Supplementary Fig. S1.

In both populations, the Anova (results reported in Table 2) detected highly significant (*P*≤0.01) differences among the RILs for all considered traits. CVs for RSA traits ranged from 5 to 13% for most recorded traits [except for LRN (lateral root number per primary root) in Mr×Cl, with a CV of 18.5%], while heritability values (*h* ^2^) were mostly between 0.62 and 0.93, with only a few traits showing *h* ^2^ values <0.50 (Table 2). CVs were consistently lower and *h* ^2^ values higher in Co×Ld as compared with Mr×Cl.

Both RIL populations showed an RSA trait distribution approaching normality and strong transgressive segregation for most traits, with the only exception for the presence of the Rt6 that showed a distribution close to bi-modality (Supplementary Fig. S1). The fold range variation in trait values among RILs varied from 0.50 to 1.20 for most traits, with maximum fold range values for Rt6 (3.63) and LRN (2.40) in both populations, followed by total root length (TRL), average root length (ARL), and SDW in Co×Ld (Table 2; Supplementary Fig. S1).

RILs showed heritabilities of medium to high values (≥0.60) for most traits, with the highest values observed for RGA (*h* ^2^=0.90 and 0.65 in Co×Ld and Mr×Cl, respectively), LRN (0.93 and 0.62, respectively), and SDW (0.91 and 0.70, respectively). Colosseo and Lloyd differed markedly for RGA, SL, SDW, primary and total root length (PRL and TRL, respectively), and presence/absence of Rt6 (Table 1).

**Table 1.** Summary of acronyms used for the traits measured in this study

Acronym	Trait	Measuring unit
***Seedling traits***		
ARL	Average root length	cm
iTGW	Thousand grain weight of the seed used in the root seedling evaluation experiment	g
LRN	Lateral root number per primary root	*n*
PRL	Primary root length	cm
PRD, TRD, ARD	Primary, total, average root diameter	mm
PRS, TRS, ARS	Primary, total, average root surface	mm^2^
PRV, TRV, ARV	Primary, total, average root volume	mm^3^
RDW	Root dry weight	mg per plant
RGA	Root growth angle	°
RSR	Root to shoot ratio	ratio
Rt6	Presence of the sixth asymmetric seminal root	% of seedlings
SDW	Shoot dry weight	mg per plant
SL	Shoot length	cm
TRL	Total root length	cm
TRN	Total root number	*n*
***Field traits***		
TGW	Thousand grain weight from the field experiments	g
GY	Grain yield	t ha^−1^
***Other acronyms***		
GWAS	Genome-wide association mapping	
RSA	Root system architecture	
RGA	Root growth angle	
NUE	Nitrogen use efficiency	
SNP	Single nucleotide polymorphism	
UNIBO-DP	UNIBO-Durum Panel	
WUE	Water use efficiency	

**Fig. 2.**
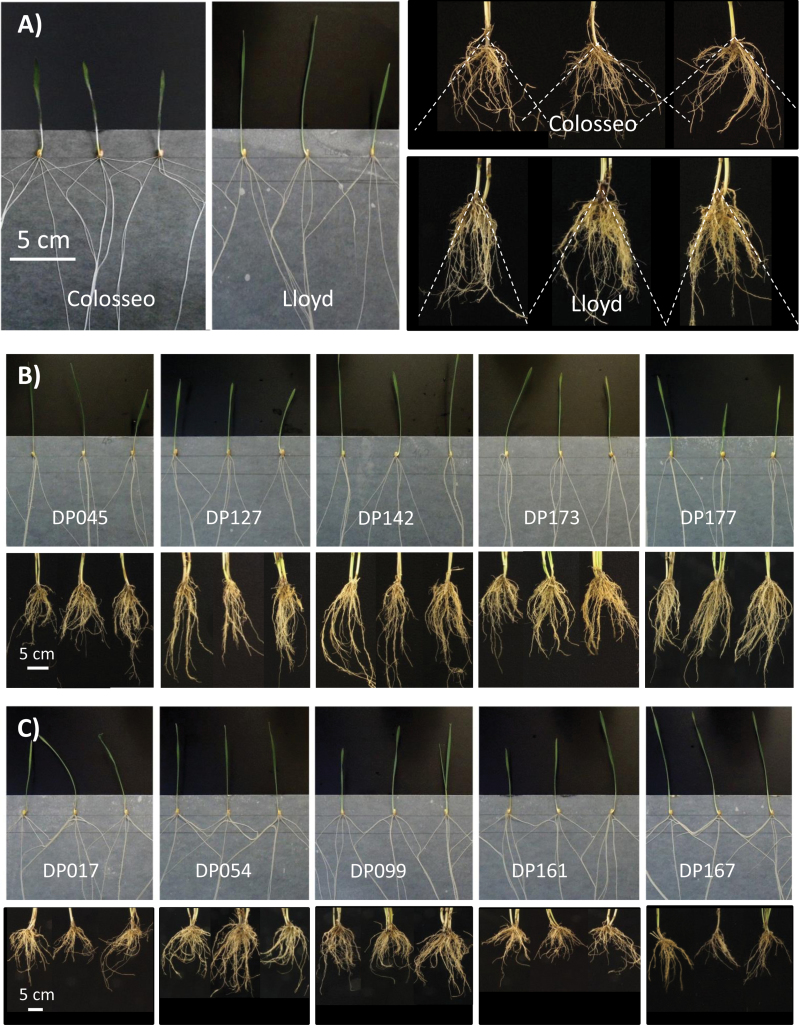
Root system architecture (RSA) of seedlings and field-grown plants of durum wheat parental lines and accessions from the elite Unibo-DP showing contrasting phenotypes for RSA features. (A) Comparison of RSA for the parental lines Colosseo (left) and Lloyd (right) at the adult stage in the field. Dotted lines originating at the crowns were traced to delimit 95% of the 2-D projected area of the root system (as suggested in the REST software manual). (B) Comparison of seedling and adult RGA for the five Unibo-DP accessions with the narrowest RGA according to seedling measurements. (C) Comparison of seedling and field RGA for the five Unibo-DP accessions with the widest RGA according to seedling measurements. (This figure is available in colour at *JXB* online.)

Among the four parents, Colosseo showed the widest RGA (40.9% wider than Lloyd, the parent with the narrowest RGA), as depicted in Fig. 1 which provides a schematic representation of the methodologies used to evaluate RGA, and in Fig. 2, which provides a comparison between RSA of Colosseo and Lloyd at both seedling and adult plant stages. As compared with Colosseo, Lloyd was characterized by significantly longer coleoptiles (SL, +29.1%) and primary roots (PRL, +32.7%), more lateral root tips (LRN, +124%), and higher SDW (42.0%, respectively), while RDW was higher in Colosseo (+49.2%). This resulted in a higher root to shoot ratio in Colosseo than in Lloyd (1.22 versus 0.64). The majority of Colosseo seedlings (70.0%) showed the presence of Rt6, while Lloyd seedlings did not develop this root. As compared with Colosseo and Lloyd, Meridiano and Claudio differed less for RSA traits, showing significant differences for TRN, TRL, ARL, LRN, and RGA, but not for PRL, Rt6, RDW, RSR, SL, and SDW (Table 2, Fig. 2).

**Table 2.** Summary statistics for the root and shoot traits measured at the seedling stage in the parents and recombinant inbred lines (RILs) of two durum wheat populations

Genotype	TGW^*a*^	TRN	Rt6	PRL	TRL	ARL	LRN	RGA	SL	SDW	RDW	RSR
	(g)	(*n*)	(%)	(cm)	(cm)	(cm)	(*n*)	(°)	(cm)	(mg per plant)	(mg per plant)	(ratio)
Colosseo×Lloyd												
Colosseo	53.5	5.6	70.0	20.5	118.2	18.1	6.9	107	11.4	13.8	16.9	1.22
Lloyd	51.3	4.8	0.0	27.2	130.8	21.2	15.5	76	14.6	18.6	12.0	0.64
RILs												
Mean	50.9	5.30	36.3	22.9	96.9	18.6	9.5	82	13.8	14.0	13.3	0.96
Minimum	40.3	4.22	0.0	14.9	52.1	10.5	3.3	47	10.7	7.6	7.6	0.72
Maximum	64.0	6.09	100.0	33.1	143.2	28.2	19.7	109	16.2	20.3	18.2	1.33
Fold range	0.47	0.35	2.75	0.79	0.94	0.95	1.73	0.76	0.40	0.91	0.80	0.64
Significance^*b*^	**	**	**	**	**	**	**	**	**	**	**	**
CV (%)	5.6	4.9	5.40	8.62	8.95	8.09	12.9	6.3	4.4	8.6	7.40	10.0
* h* ^2^	0.87	0.73	0.88	0.48	0.63	0.62	0.93	0.90	0.84	0.82	0.76	0.66
Meridiano×Claudio												
Meridiano	51.6	5.6	30.2	32.9	133.9	22.5	2.2	85	13.7	13.5	12.3	0.91
Claudio	54.0	4.9	28.5	32.7	117.4	26.5	15.0	104	13.8	12.4	13.3	1.07
RILs												
Mean	48.3	5.0	16.0	30.7	122.5	22.9	10.4	98	14.7	12.2	11.6	0.96
Minimum	39.8	4.4	0.0	24.7	95.8	17.7	2.2	68	10.9	9.6	8.6	0.70
Maximum	63.4	5.7	58.1	34.5	142.4	26.2	27.2	129	15.6	14.7	13.7	1.22
Fold range	0.49	0.26	3.63	0.32	0.38	0.37	2.40	0.61	0.32	0.42	0.44	0.54
Significance^*b*^	**	**	**	**	**	**	**	**	**	**	*	**
CV (%)	6.4	13.4	13.5	5.7	7.12	6.6	18.5	13.5	7.0	12.6	13.8	13.0
* h* ^2^	0.79	0.62	0.64	0.52	0.56	0.32	0.62	0.65	0.64	0.70	0.62	0.56

^*a*^ Root trait acronyms: TGW, thousand grain weight; TRN, total root number; Rt6, presence of the sixth root; TRL, total root length; ARL, average root length; LRN, lateral root number on the seminal primary root; RGA, root growth angle; SL, shoot length; SDW, shoot dry weight; RDW, root dry weight; RSR, root to shoot ratio.

^*b*^ Significance of differences among RIL lines: *P≤0.05; **P≤ 0.01.

The phenotypic values for RSA traits observed in the two mapping populations were in the range of those observed for the Unibo-DP as reported in Canè *et al.* (2014). Notably, for RGA, the panel showed a wider range of values than those observed in the two RIL populations (48–147 ° versus 47–110 ° in Co×Ld and 68–129 ° in Mr×Cd), as depicted in Fig. 2.

**Fig. 3.**
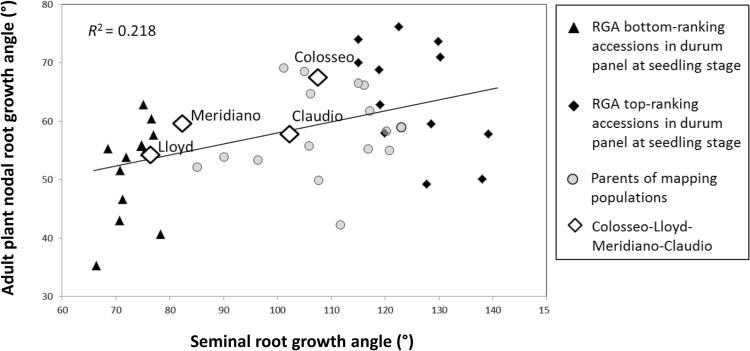
Relationship between seminal root growth angle (RGA) at the seedling stage and adventitious root system growth in the adult plant under field conditions for a selection of 44 Unibo-DP accessions. Three accession groups were considered: (i) 12 Unibo-DP accessions bottom-ranking for seminal RGA; (ii) 12 Unibo-DP accessions top-ranking for seminal RGA; and (iii) 16 parents of mapping populations developed at UNIBO plus the parental lines of the Colosseo×Lloyd and Meridiano×Claudio RIL populations.

The relationship between RGA evaluated at the seedling stage and RGA at the adult stage measured under field conditions (nodal roots) was assessed on selected contrasting ‘tails’ from the RGA phenotypic distribution of the Unibo-DP accessions and in a small panel of parents of mapping populations. Summary statistics of comparison between RGA of seminal roots at the seedling stage and of the adult root system from the field are reported in Table 3. On average, the RGA of adult root systems under field conditions is narrower than the RGA on seminal roots on screening plates. The group of accessions selected for narrow seedling RGA showed a mean field RGA equal to 51 ° versus a mean seedling RGA of 73 ° (with a 29.7% reduction for field RGA) while the group selected for wide seedling RGA had a mean field RGA equal to 64 ° versus a mean seedling RGA of 125 ° (48.7% reduction for field RGA). The parents of mapping populations showed a mean field RGA equal to 58 ° and a mean seedling RGA equal to 106 ° (44.9% reduction for field RGA). The regression of adult RGA on seedling RGA was highly significant, with an *R* ^2^ value equal to 21.8% (Fig. 3). Correlations between RSA traits are reported in Supplementary Table S1 and commented on in Supplementary Text S3.

**Table 3.** Summary statistics for the root growth angle (RGA) at seedling and adult plant stages in 24 selected accessions of the Unibo-DP and in 16 accessions used as parents of mapping populations

Accessions	Seedling(°)	Field^*a*^(°)
Narrow RGA (12 accessions)^*b*^		
Mean	73.1	51.4
Minimum	66.3	35.3
Maximum	78.3	62.8
CV (%)	*10.4*	*17.7*
Wide RGA (12 accessions)		
Mean	125.3	64.2
Minimum	115.0	49.3
Maximum	139.2	76.2
CV (%)	12.4	15.9
Parental lines (16 accessions)		
Mean	106.1	58.4
Minimum	82.5	42.3
Maximum	120.7	69.2
CV (%)	12.0	17.4
Colosseo	107.1	67.1
Lloyd	76.0	53.9
Meridiano	85.2	60.8
Claudio	104.4	58.5

^*a*^ Field RGA was evaluated at the end of flowering stage (Zadok 69) in a field trial carried out in a high-fertility alluvial loam soil (Cadriano, Italy).

^*b*^ Summary statistics are reported for three groups of accessions: (i) 12 bottom-ranking accessions from the 183 Unibo-DP accessions sorted for seminal RGA (Canè *et al.*, 2014); (ii) 12 top-ranking accessions from the 183 Unibo-DP accessions sorted for seminal RGA (Canè *et al.*, 2014); and (iii) 20 parents of RIL populations being developed at UNIBO, including Colosseo, Lloyd, Meridiano, and Claudio parents.

### QTLs for root and shoot traits

Two high-density linkage maps were produced for the Co×Ld and Mr×Cd populations, including 7946 and 5970 mapped markers, respectively. Total map length was 2064 cM for Co×Ld and 2248 cM for Mr×Cd. Each map covered ~70% of the durum wheat genome relative to the reference consensus map (Maccaferri *et al.*, 2015), hence revealing extensive identity by descent regions. In the Unibo-DP, a total of 13 196 polymorphic SNPs showed a minimum allele frequency (MAF) ≥0.05 and were uniquely mapped in the consensus map.

For all traits, both RIL populations showed numerous QTLs (Supplementary Tables S2, S3). The Co×Ld population showed 48 QTLs (20 suggestive and 28 nominal QTLs) which were grouped into 26 QTL clusters. The Mr×Cd population showed 81 QTLs (49 suggestive and 32 nominal QTLs), for a total of 44 QTL clusters.

The detected QTLs showed a broad range of *R* ^2^ values (from 4.0 to 26.9%, Supplementary Tables S2, S3). Determination of the distribution of QTLs between the two genomes showed a relative enrichment for the B genome, with 28 and 42 QTL clusters found in the A and B genome, respectively.

The association mapping panel (not phenotyped for LRN, nor root diameter, surface, and volume) showed 201 QTLs, five of which reached the experiment-wise threshold (–log *P*≥4), 26 exceeded the marker-wise threshold of –log *P*≥3, and 170 had a marker-wise –log *P*-value of between 2 and 3 (suggestive QTLs). The GWAS QTLs from the first two categories were considered as nominal QTLs, while the remaining 170 were classified as suggestive QTLs. All the GWAS QTLs were then grouped into 112 QTL clusters. Considering RSA traits as a whole, the high-density SNP genotyping allowed us to detect many additional QTL clusters as compared with (112 versus 48) those reported in Canè *et al*. (2014) using SSR and DArT^®^. However, the relative QTL mapping efficiency of SNP versus SSR and DArT^®^ varied depending on the trait: for RGA, a highly heritable trait, 76% of the QTLs detected with the high-density SNP map were also detected by Canè *et al*. (2014). Details of GWAS QTL results for the RSA traits are reported in Supplementary Table S4.

All the QTL information was cross-referenced by projecting the QTL confidence intervals on the tetraploid wheat consensus map (Maccaferri *et al.*, 2015). Other RSA QTLs from published studies in wheat were also cross-referenced, whenever possible, by projecting common markers onto the tetraploid wheat consensus. Chromosome arms with a high number of RSA QTLs were 1AS, 1BS, 2A centromeric, 2BS, 2BL, 3A centromeric, 3AL, 3BS, 5AS, 5BS, 6AS, 6BS, 6BL, 7AS, and 7BL (Supplementary Fig. S2).

### Main QTL clusters for root length and number

The prioritization of RSA QTL clusters relied on the significance level of single-component QTLs, the consistency of their effects on root traits across durum and bread wheat, as well as the presence of significant overlap with field TGW and GY from data of the 15 Mediterranean environments available for the Unibo-DP (Maccaferri *et al.*, 2011). Due to the genetic control of RGA which is largely independent from that of the other RSA traits, QTLs for RGA have been prioritized separately.

**Fig. 4.**
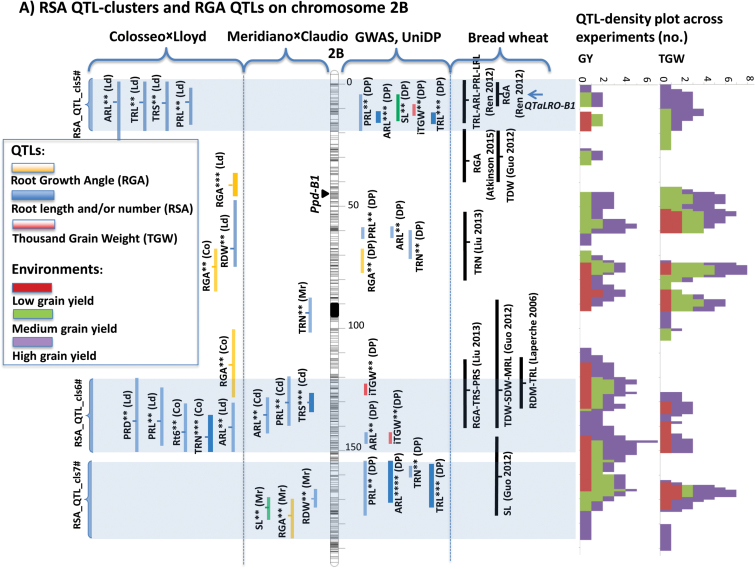

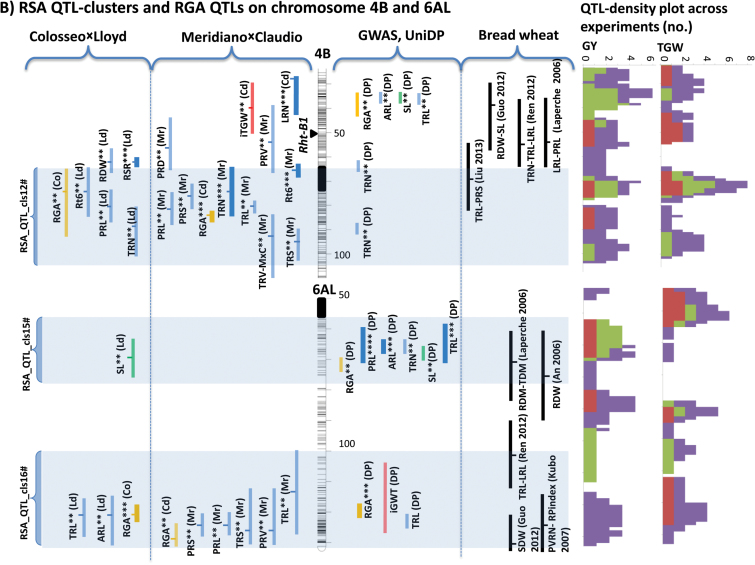

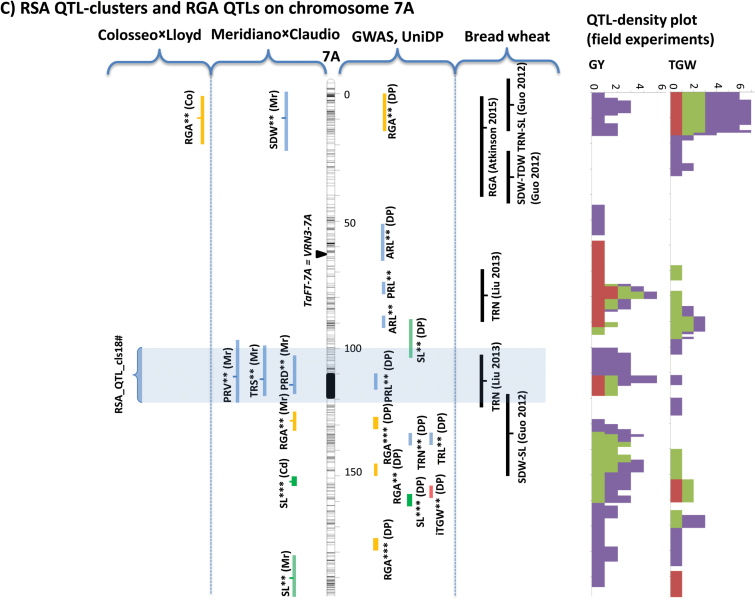
Genetic maps of root system architecture (RSA) QTL clusters and root growth angle (RGA) QTLs for chromosomes 2B, 4B, 6A, and 7A. The reference map is the tetraploid consensus map reported by Maccaferri *et al.* (2015). RSA and RGA QTLs from Co×Ld and Mr×Cl RIL populations, GWAS QTLs from Unibo-DP. and previously published QTLs from bread wheat studies have been projected onto the reference maps. Single-component QTLs are reported as vertical bars corresponding to confidence intervals. RSA QTL clusters are highlighted by horizontal shaded banding. GY and TGW QTLs in the trait acronyms are the same as in Table 1. QTL significance levels are highlighted using ** for suggestive QTLs, *** for nominal QTLs, and **** for GWAS experiment-wise significant QTLs (Unibo-DP only).

Among the 85 RSA QTL clusters for root vigour (root length, weight, and/or number) identified in this study, 20 appeared of particular interest based on the cumulated proof for the presence of RSA QTLs across the genetic materials that were explored and co-location with GY QTLs in drought-stressed environments. Features of these QTL clusters are summarized in Table 2 and detailed in Supplementary Fig. S2. Examples of QTL cluster maps for chromosomes 2B, 4B, 6A, and 7A are provided in Fig. 4. Twelve clusters showed consistent effects on both root length and number (RSA_QTL_clusters_2#, 3#, 4#, 6#, 7#, 8#, 11#, 12#, 13#, 14#, 17#, and 18#). Additionally, RSA_QTL_clusters_1#, 5#, 9#, 10#, 15#, 16#, 19#, and 20# were considered specific for root length/elongation.

### QTLs for root growth angle

Biparental mapping identified 12 RGA QTLs, six in Co×Ld and six in Mr×Cd. Details of RGA QTL features are reported in Table 5. As expected based on the four parental phenotypes, the narrow-angle phenotype was mostly contributed by Lloyd. Three QTLs were classified as major based on additive effects on root angle ≥10 ° and LOD score >3: *QRga.ubo-2B* and *QRga.ubo-6A* in Co×Ld (*R* ^2^=11.7% and 17.8%, respectively) and *QRga.ubo-4B* in Mr×Cd (*R* ^2^=13.8%, see Table 5).

**Table 4.** Main QTL clusters for root system architecture (RSA) traits identified across RIL (linkage mapping), Unibo-DP (association mapping), and projected wheat QTLs from other studies Seminal root growth angle (RGA) QTLs are not included in the table.

RSA QTL cluster	Chr.	Interval^*a*^ (cM)	Main RSA trait^*b*^	Colosseo×Lloyd^*c*^ (trait)	Meridiano×Claudio (trait)	Unibo-DP (trait)	Wheat QTLs (trait, reference)^*d*^
*RSA_QTL_cluster_1#* ^*e*^	1AS	0–20	RL	–	PRV, TRV	TRN, TGW	TRL, TRS, TRV, LRN (Tt1)
*RSA_QTL_cluster_2#*	1BS	0–25	RL, RN	TRL, TRV	TRS, TRV	ARL, SL, TRL, TRN	TRL, TRS, TRV, LRN (Tt1), TRL (Ta10)
*RSA_QTL_cluster_3#*	1BS	30–50	RL, RN	–	ARL, TRL	TGW	LRN (Tt1), TRN (Ta3), TRL (Ta6), TDW, SDW, RDW, TRN (Ta7), RDW (Tt11)
*RSA_QTL_cluster_4#*	2AL	190–210	RL, RN, RGA	–	TRV, LRN, TGW	ARL, TRL, TRN, PRL, TGW, RGA	–
*RSA_QTL_cluster_5#*	2BS	0–20	RL	ARL, PRL, TRL, TRS	–	ARL, SL, PRL, TRL, TGW	TRL, PRL, LRL, qTaLRO-B1 (Ta2-9)
*RSA_QTL_cluster_6#*	2BL	120–150	RL, RN	ARL, PRD, PRL, Rt6, TRN	ARL, PRL, TRS	TGW	RGA, TRS, PRS (Ta6), TDW, SDW, MRL (Ta7), RDM, TRL (Ta10)
*RSA_QTL_cluster_7#*	2BL	155–185	RL, RN, RGA	–	RDW, SL, RGA	ARL, PRL, TRL, TRN	SL (Ta7)
*RSA_QTL_cluster_8#*	3AS	30–40	RL, RN	–	SL	ARL, PRL, TRL, TRN	–
*RSA_QTL_cluster_9#*	3AL	100–135	RL	–	–	ARL, PRL, TRL, TGW	TRL, TRS, TRV (Tt1), LRL, LRV, LRS (Ta5), MRL (Ta7), PRL (Ta9)
*RSA_QTL_cluster_10#*	3BS	0–10	RL	–	PRS	ARL, PRL, SL, TRL, TGW	–
*RSA_QTL_cluster_11#*	3BL	70–100	RL, RN	–	ARL, TRL	–	TRL, TRV, LRV, LRS (Ta5), TRL, PRL, LRV, TRS, TRN, RGA (Ta6)
*RSA_QTL_cluster_12#*	4BL	65–105	RL, RN	Rt6, PRL, TRN, RGA	Rt6, PRL, TRL, TRV, TRN, PRS, RGA	TRN	TRL, PRS (Ta6)
*RSA_QTL_cluster_13#*	5AS	25–65	RL, RN	Rt6, PRL, TRN	–	–	TRV, TRS, SDW, TGW (Ta5), TRN (Ta6), TRN (Ta7), TRN (Ta8)
*RSA_QTL_cluster_14#*	5AL	100–140	RL, RN	–	LRN, SDW	ARL, PRL, TRL	–
*RSA_QTL_cluster_15#*	6AL	55–75	RL	–	–	ARL, PRL, SL, TRL, TRN	RDM/TDM (Ta10)
*RSA_QTL_cluster_16#*	6AL	100–130	RL	ARL, TRL	PRL, TRL, PRS, TRS, PRV	TRL	SDW (Ta7), PVRN, RPindex (Tt11)
*RSA_QTL_cluster_17#*	6BL	75–100	RL, RN, SL	ARL, TRD, SL	ARL, PRL, PRS, ARL, Rt6	TRL	MRL (Ta7)
*RSA_QTL_cluster_18#*	7AL	100–120	RL, RN	–	PRD, PRV, TRS	PRL	TRN (Ta6)
*RSA_QTL_cluster_19#*	7BL	155–175	RL	–	PRS, PRL	PRL, RGA	TRL (Ta6), RDW, SDW, TDW, MRL (Ta7)
*RSA_QTL_cluster_20#*	7BL	185–220	RL	TRL, TRS, ARL	–	–	TDW, RDW (Ta7), LRN/PRL (Ta10)

^*a*^ Chromosome interval refers to the tetraploid wheat consensus map (Maccaferri *et al.*, 2015).

^*b*^ Main RSA trait acronyms: RL, root length; RN, root number; RGA, root growth angle.

^*c*^ Root trait acronyms: TGW, thousand grain weight; TRN, total root number; Rt6, presence of the sixth root; TRL, total root length; ARL, average root length; LRN, lateral root number on the seminal primary root; RGA, root growth angle; SL, shoot length; SDW, shoot dry weight; RDW, root dry weight; RSR, root to shoot ratio.

^*d*^ Literature references are listed in full in Supplementary Fig. S2.

^*e*^ The QTL cluster acronyms have been differentiated from the nomenclature currently used for single-trait QTLs by using consecutive numbering followed by the # symbol.

**Table 5.** QTLs for root growth angle (RGA) identified across Colosseo×Lloyd and Meridiano×Claudio RIL populations, the association panel (Unibo-DP), and projected wheat QTLs

SRA QTL	Chr.	CI^*a*^	QTL-tag SNP	Co×Ld^*b*^	SNP^*c*^	Mr×Cl^*d*^	SNP^*e*^	Association panel^*f*^	SNP^*g*^	Wheat QTLs^*h*^
		(cM)	(ID)	(effect, *R* ^2^, isignif.i)^i^	(allele)	(effect, *R* ^2^, signif.)	(allele)	(effect, *R* ^2^, signif.)	(allele)	(reference)
*QRga.ubo-1B*	1B	61–62–68	*IWA7700*	NS – –	C/T	NS – –	T/T	+7.2 (4.2) **	C/***T***	
*QRga.ubo-2A.1*	2A	7–10–12	*IWB16988*	NS – ––	T/T	NS – ––	T/T	+7.7 (4.7) **	C/***T***	
*QRga.ubo-2A.2*	2A	97–99–102	*IWA1597*	NS	T/T	NS	T/T	+10.8 (3.9) **	C/***T***	(Ta6)
*QRga.ubo-2A.3*	2A	208–211–213	*IWB20424*	NS	A/A	NS	A/A	+9.5 (7.5) ***	***A***/G	(Ta3)
*QRga.ubo-2B.1*	2B	37–43–45	*IWB4604*	–10.0 (11.7) ***	T/C	NS	T/C	NS	C/***T***	
*QRga.ubo-2B.2*	2B	70–72–75	*IWB73449*	NS	A/A	NS	G/G	+8.2 (6.2) **	A/***G***	
*QRga.ubo-2B.3*	2B	68–77–87	*IWB39220*	+6.0 (6.2) **	A/A	NS	G/G	NS	A/***G***	
*QRga.ubo-2B.4*	2B	100–114–130	*IWB62718*	+5.0 (4.4) **	G/A	NS	A/A	NS	***A***/G	(Ta6)
*QRga.ubo-2B.5*	2B	175–176–183	*IWB13830 = IWB7663*	NS	A/G	+5.8 (6.7) ***	A/G	+4.70 (2.4) *	A/***G***	
*QRga.ubo-3A*	3A	171–173–176	*IWB70610*	NS	A/A	NS	A/A	–6.9 (5.5) **	***A***/G	
*QRga.ubo-3B*	3B	181–188–196	*wPt-5947*	NS	1/0	–4.6 (4.2) **	1/0	NS	–	
*QRga.ubo-4A*	4A	154–156–159	*wPt-0538*	NS	1/1	NS	1/1	–12.5 (4.2) **	***1/***0	
*QRga.ubo-4B.1*	4B	33–35–38	*IWB73831*	NS	T/C	NS	T/C	–6.7 (4.6) **	***C***/T	
*QRga.ubo-4B.2*	4B	44–47–49	*IWB69501*	NS	T/C	NS	C/C	–8.6 (3.8) **	***C***/T	
*QRga.ubo-4B.3*	4B	66–75–91	*IWB60481*	+4.2 (2.4) **	C/T	NS	C/C	NS	***C***/T	
*QRga.ubo-4B.4*	4B	82–82–86	*IWB12276*	NS	A/A	–8.2 (13.8) ***	A/G	NS	***A***/G	
*QRga.ubo-5B.1*	5B	2–3–13	*IWB75279*	NS	1/1	–5.6 (6.1) ***	0/1	NS	***1***/0	
*QRga.ubo-5B.2*	5B	47–49–52	*IWB24957*	NS	C/C	NS	C/C	+15.0 (4.6) **	***C***/T	
*QRga.ubo-6A.1*	6A	70–72–75	*IWB50963*	NS	A/G	NS	A/G	–11.9 (5.2) **	***A***/G	
*QRga.ubo-6A.2*	6A	119–122–123	*IWB71119*	+10.0 (17.8) ***	G/A	NS	G/A	–10.6 (8.8) ***	***A***/G	
*QRga.ubo-6A.3*	6A	127–130–136	*IWB24237*	NS	T/T	–5.8 (7.0) **	T/C	NS	C/***T***	
*QRga.ubo-6B.1*	6B	20–22–25	*IWB62692*	NS	A/A	NS	A/A	+6.8 (4.8) **	***A***/G	(Ta3)
*QRga.ubo-6B.2*	6B	143–145–147	*IWB13062*	NS	G/G	NS	G/G	–7.0 (4.5) **	A/***G***	(Ta3)
*QRga.ubo-7A.1*	7A	4–10–21	*wPt-9207*	+6.0 (5.5) ***	0/1	NS	1/1	NS	***1***/0	(Ta4)
*QRga.ubo-7A.2*	7A	128–130–133	*IWA2752*	NS	C/C	NS	C/C	+11.8 (7.1) ***	***C***/T	
*QRga.ubo-7A.3*	7A	125–131–135	*IWB35428*	NS	T/T	+5.6 (6.5) **	C/–	NS	C/***T***	
*QRga.ubo-7A.4*	7A	145–147–150	*IWB56095*	NS	C/T	NS	–/C	–7.2 (5.3) **	***C***/T	
*QRga.ubo-7A.5*	7A	177–180–183	*IWB3169*	NS	C/C	NS	T/C	+10.4 (7.2) ***	***C***/T	
*QRga.ubo-7B*	7B	175–177–180	*IWB371*	NS	G/G	NS	G/G	+12.8 (5.1) **	A/***G***	

^*a*^ Refers to the tetraploid wheat consensus map (Maccaferri *et al.*, 2015).

^*b*^ Allelic effect reported as double-dose substitution of the Colosseo versus Lloyd allele. A positive sign indicates that the Colosseo allele increases the trait, and vice versa.

^*c*^ SNP alleles in the RIL population: Colosseo allele (first)/Lloyd allele (second).

^*d*^ SNP allelic effect (*a*) reported as double-dose substitution (*2a*) of the Meridiano versus Claudio allele. A positive sign indicates that the Meridiano allele increases the trait and vice versa; NS, non-significant.

^*e*^ SNP alleles in the mapping population: Meridiano allele (first)/Claudio allele (second).

^*f*^ SNP allelic effect (*a*) reported as double-dose (*2a*) substitution of the A or C allele (reported first) versus the G or T allele (reported second).

^*g*^ SNP alleles in Unibo-DP. The allele with the highest frequency is reported as bold italic font.

^*h*^ Literature references are listed in Supplementary Fig. S2.

^*i*^ RGA is expressed as degress while *R* ^2^ is expressed as a percentage; QTL effect significance; RIL populations, **LOD 2–3; ***LOD>3; GWAS in Unibo-DP, **marker-wise *P*≤0.01; ***marker-wise *P≤*0.001; ****experiment-wise *P*≤0.05 (=marker-wise *P≤*0.0001).

As compared with the mapping populations, GWAS on the Unibo-DP revealed 17 novel RGA QTLs, nine of which showed an *R* ^2^ coefficient >5.0% (Table 5). Therefore, limited overlap was observed between the QTLs identified in biparental and association mapping. The only QTL that showed a tight co-location between one mapping population (Co×Ld) and the association panel was *QRga.ubo-6A.2*. However, overlap between RGA QTLs in tetraploid wheat and previously reported QTLs in bread wheat was observed on chromosomes 2A, 2B (two cases), 3B, 6B (two cases), and 7A.

Details of RGA phenotypic data and RGA QTL-tagging SNP allelic distribution in the Unibo-DP germplasm subgroups are reported in Supplementary Tables S5 and S6. A schematic representation of contrasting shallow and deep wheat root ideotypes with the QTLs for seminal RGA identified in this study which can potentially contribute (through marker-assisted selection) is provided in Supplementary Fig. S4. The SNP alleles for narrow RGA were present at high frequency (≥0.5) for nine QTLs, at balanced frequency (0.20–0.50) for seven QTLs, and at relatively low frequency (0.10–0.20) for four QTLs only. This indicates that the alleles contributing a narrow RGA are already present at relatively high frequencies in the cultivated accessions. Here, it is also worth noting that QTL effect and significance inferences in association mapping are both influenced by allele frequency in populations, particularly as to the precision of allelic variant effect estimates and the robustness of association. Considering *QRga.ubo-6A.2*, which is one of the most important RGA QTLs found both in Col×Ld and Unibo-DP at high *R* ^2^ levels, it can be noticed that the narrow-angle allele was absent in the ICARDA germplasm for dryland areas (Syrian germplasm, directly derived from the West Asian native germplasm) while being present at higher than average frequencies in the modern high-yielding germplasm including the most recent germplasm from CIMMYT/ICARDA programmes and the Italian and DesertDurum^®^ cultivars. A similar allelic distribution was observed for the second most relevant QTL found in the association panel, *QRga.ubo-2A* (Supplementary Table S6).

Additionally, interesting QTLs with sizeable effects (≥10 °) on RGA were identified by GWAS on chromosomes 1B, 2A, and 7A. For these QTLs, the narrow-angle allele was present at low frequency in the elite germplasm.

### Relationships between QTLs for RSA, GY, and TGW

Co-locations between RSA QTLs and QTLs for TGW and GY were assessed based on the GWAS reanalysis of field data from the evaluation of the Unibo-DP in 15 field trials under a wide range of water availability and grain yield (from 0.99 t ha^−1^ to 6.74 t ha^−1^) in the Mediterranean region (for more details, see Maccaferri *et al.*, 2011). In our study, the addition of 13 196 SNPs to the 957 SSR and DArT markers used by Canè *et al.* (2014) greatly improved detection of QTLs for GY, a trait with medium to low heritability (*h* ^2^ from 0.42 to 0.67) and TGW.

Among the 20 RSA QTL clusters identified in this study, only two had no impact on either field GY or TGW (QTL_cls. 14# on chromosome 5A and 20# on chromosome 7B), while five were associated with peaks in GY but not final TGW (QTL_cls. 1#, 11#, 15#, 18#, and 19#) and six clusters co-located only with TGW (QTL_cls. 5#, 8#, 9#, 10#, 13#, and 17#). Finally, six QTLs co-located with both GY and TGW QTLs (QTL_cls. 3#, 4#, 6#, 7#, 12#, and 16#). Supplementary Fig. S3 reports the detailed co-location between RSA, GY, and TGW QTLs.

Among the 30 RGA QTLs, nine (*QRGA.ubo-2A.1*, *QRGA.ubo-2A.2*, *QRGA.ubo-2A.3*, *QRGA.ubo-2B.2/2B.3*, *QRGA.ubo-6A.2*, *QRGA.ubo-7A.1*, and *QRGA.ubo-7A.2*/*7A.3*, with the *2B.2/2B.3* and *7A.2/7A.3* QTLs to be considered as different QTLs with very close confidence intervals) mapped within relatively small intervals (≤10 cM) that co-localized with strong QTL peaks for GY and TGW, including GY in 3–4 environments and TGW in 4–7 environments, respectively (Supplementary Fig. S3). For *QRGA.ubo-2A.1*, *QRGA.ubo-2B.2/2B.3*, and *QRGA.ubo-7A.1*, it is worth noting that the co-locations of RGA QTLs with those for TGW and GY were specific for RGA.

For each RGA QTL with an *R* ^2^ value ≥ 5.0% among those identified in the Unibo-DP, Supplementary Table S7 reports the concomitant effects on GY and TGW, based on the allelic variants present at the QTL-tag SNPs. Notably, the QTL alleles for wider root angle had a prevailingly negative effect on GY and/or TGW in the less favourable environments. The negative RGA versus GY association was observed for 35 (i.e. 49%) of the 72 QTL–environment pairs in eight drought- and heat-stressed environments (from Granada-2005, rainfed, to Tel Adja-2005, rainfed, for a total of eight environments with mean GY from 0.99 t ha^−1^ to 4.63 t ha^−1^). Conversely, the same negative association was observed in only 17 (i.e. 27%) of the 63 QTL–enviroments pairs in seven medium to highly productive environments (from Rayack-2005, rainfed, to Kef-2005, irrigated, with mean GY from 5.61 t ha^−1^ to 6.78 t ha^−1^). Moreover, the RGA versus GY association was consistently negative across the majority of the RGA QTLs in the most stressed environment (Granada-2005, rainfed) and for another environment with low GY (Tel-Adja-2005, rainfed). Importantly, *QRga.ubo-7B*, *QRga.ubo-6A.1*, and *QRga.ubo-7A.4* showed a consistently negative RGA versus GY association of effect across the majority of environments, including the medium- to high-yielding ones, while the QTLs *QRga.ubo-7A.5*, *QRga.ubo-2B.2*, and *QRga.ubo-3A* showed a negative RGA versus GY association mostly in highly stressed environments. The relationship between RGA and TGW was not as clear as that with GY, possibly due to the highly compensating interaction between yield components in wheat.

## Discussion

Our study reports novel QTLs for RSA traits at the seedling stage in tetraploid wheat, provides a comprehensive survey of the QTLome for RSA features in wheat, and demonstrates that RSA phenotypes such as root length and RGA are governed by allelic variation at major and minor QTLs.

From a methodological standpoint, phenotyping seminal roots proved to be highly accurate and cost-effective for evaluating hundreds of lines (540 in total) as required in QTL discovery studies. Previous studies have investigated RSA on seedlings in wheat and other crops and have shown that QTLs for root features at this stage can be predictive of RSA of field-grown plants (Tuberosa *et al.*, 2002; Landi *et al.*, 2010; Mace *et al.*, 2012; Li *et al.*, 2015; Richard *et al.*, 2015; Uga *et al.*, 2015). Notably, in wheat and barley it has been demonstrated that the functional importance of the seminal roots as compared with the nodal roots increases under drought and other environmental stress (Belford, 1987; Manske and Vlek, 2002; Manschadi *et al.*, 2010). An actively growing seminal root apparatus is important for early vigour and crop establishment in dryland areas (Lopez-Castaneda *et al.*, 1995; Reynolds and Tuberosa, 2008). The seminal root system also plays an important role under high-density planting in high-input conditions (Manske and Vlek, 2002).

The main core of the results and QTLs reported herein refer to phenotypic characterization and QTL identification carried out on wheat seminal roots at an early growth stage (9-day-old seedlings grown in water). The objective here is to identify QTLs with strong and constitutive expression, in order to be able to translate their effects to wheat plants grown under various conditions, including water- and nutrient-stressed fields. Heritability in experiments carried out under standard controlled conditions is higher as compared with experiments where the environmental component of variation is more sizeable, such as in the case of seedlings grown in soil and/or at contrasting temperatures. Environment-specific, adaptive QTLs can be missed by analysis under standard conditions. However, this is counterbalanced by gains in reproducibility and the capability to assess effectively the genetic value of the trait, therefore increasing the capacity to detect the underlying constitutive QTLs (Collins *et al.* 2008).

### GWAS in cultivated durum wheat germplasm

In wheat, QTL detection and genomics-assisted applications have recently benefited from novel marker platforms (Tuberosa and Pozniak, 2014) such as the Illumina 90K wheat SNP array, that allows for highly accurate genome-wide scans at 0.2 cM per marker (Wang *et al.* 2014; Maccaferri *et al.*, 2015), hence greatly facilitating and enhancing the accuracy of comparative analysis and QTL cross-referencing.

The analysis of intermarker LD (the genetic association between markers specifically estimated in the Unibo-DP genotypes) based on the high-density SNP map confirmed that LD was found to decay to the threshold value of *r* ^2^=0.3 (the generally accepted limit to detect association with a QTL) at 2.20 cM on average, as with DArT markers (Maccaferri *et al.*, 2014). This distance has been used to set a general confidence interval for the GWAS QTLs (=4.40 cM). The LD is therefore estimated to extend at the centiMorgan scale which is in accordance with previous observations for wheat and barley (Muñoz-Amatriain *et al.*, 2014; Maccaferri *et al.*, 2015). The QTL mapping resolution in the panel is thus improved compared with recombinant inbred populations where confidence intervals are mostly within 10–20 cM. The average mapping density of informative SNPs in the Unibo-DP (5.32 SNPs cM^–1^) is correspondingly high, allowing for nearly complete genome coverage and high confidence in QTL identification. Similarly to maize experiments, QTLs are identified as multiple tightly linked significant SNPs in strong LD and associated with the trait, and rarely as singletons. The outcomes, in terms of power and accuracy of QTL discovery, are considerably better than what was allowed by previous marker technology (SSR and DART; Maccaferri *et al.*, 2014). Accordingly, as compared with the study of Canè *et al.* (2014), the SNP-based analysis revealed twice as many RSA QTLs as in the previous SSR- and DArT-based analysis.

### Phenotypic variation for RSA traits and identification of QTL clusters for early root length and number and their associated effects on GY and TGW

The most well-known and striking example of selection-driven RSA manipulation in wheat is the introgression of the rye/wheat 1RS.1BL, which is now used worldwide (Howell *et al.*, 2014). In this case, improved adaptation to water stress is due to a deeper root system already detectable at early developmental stages (Sharma *et al.*, 2010; Edhaie *et al.*, 2014; Howell *et al.*, 2014). In other cases, phenotypic variation for RSA observed in RIL populations and diversity panels at the seedling stage (in particular for seminal root length evaluated in rolled germination paper) correlated with measurements carried out on field-grown roots (shovel or extracted soil cores) at vegetative stages up to *r*=0.6–0.8, while the correlation disappeared at the flowering stage (Watt *et al.*, 2013). However, these observations were not targeted to any specific QTLs. In our study we searched for QTLs expressed at the seedling stage under non-stressed conditions, aiming at identifying constitutively expressed QTLs whose effects are eventually maintained in field-grown plants, at either the vegetative or the reproductive stage. Remarkably, the two mapping populations evaluated in our study showed eight major QTLs for root number and length and three major RGA QTLs with *R* ^2^>10%.

Linkage and association mapping proved complementary and effective for dissecting the RSA QTLome in durum wheat, highlighting the presence of multiple QTLs, structured into QTL clusters, that could represent either single causal loci with pleiotropic effects on multiple traits or tightly linked loci not resolved by recombination (Tuberosa *et al.*, 2002).

Notably, limited overlap occurred among QTLs found in mapping populations and GWAS, particularly for RGA. This underlines the importance of undertaking both mapping approaches for a more comprehensive investigation of the QTLome. In this context, QTL pyramiding and/or genomic selection approaches involving multiparental populations (Delhaize *et al.*, 2015; Milner *et al.*, 2015) can be envisaged.

QTL clusters for root length and number at the seedling stage are potential candidates for marker-assisted breeding applications aimed at enhancing early rooting capacity. Chromosomes 1A, 1B, 2B, 3A, 3B, 4B, 5A, 6A, 7A, and 7B harboured the most interesting QTL clusters. Prioritizing QTLs for applying marker-assisted selection to enhance crop productivity should be based on the following factors: (i) evidence for significant QTL effects in different genetic materials; (ii) size of the additive effect and *R* ^2^ value of the QTL; and (iii) consistency of QTL effects on GY across a broad range of environments (Collins *et al.*, 2008; Tuberosa, 2012). Based on these premises, previous QTL results on GY and TGW in durum wheat have been compared with the results on RSA QTLs reported herein. The QTL clusters on chromosomes 1A, 1B, 3A, and 6B overlapped with QTLs previously reported in durum wheat by Petrarulo *et al.* (2015) using the Creso×Pedroso mapping population. QTL clusters on chromosomes 1B, 2B, 4B, and 6A were particularly interesting. The *RSA_QTLcluster_2#* (chromosome 1BS, distal region) features QTLs from Co×Ld, Mr×Cd, the Unibo-DP, Creso×Pedroso (Petrarulo *et al.*, 2015), and the bread wheat population Arche×Recital, with all QTL peaks within a 15 cM interval. For this QTL cluster, GWAS showed little or no effect on GY and final TGW. Conversely, based on their concomitant effects on GY and final TGW, the following RSA QTL clusters appear more valuable for breeding purposes: *RSA_QTLcluster_4#* on chromosome 2AL; *RSA_QTLcluster_5#*, *6#*, and *7#* on chromosome 2B; *RSA_QTLcluster_12#* on chromosome 4B; and *RSA_QTLcluster_15#* and *16#* on chromosome 6A. In particular, *RSA_QTLcluster_4#*, *5#*, *7#*, *12#*, and *15#* showed the strongest consistent effects on GY and TGW, mostly in medium- to high-stress environments. The *RSA_QTLcluster_5#* (chromosome 2BS, distal region) includes QTLs from Co×Ld, GWAS QTLs from the Unibo-DP, and projected QTLs from the hexaploid wheat population Xiaoyan54×Jing 411, which led to the identification of *qTaLRO-B1*, the only major QTL for RSA in wheat for which a functional basis has been provided (He *et al.*, 2014). Notably, the QTL peaks from tetraploid populations are mostly coincident with the map location of *qTaLRO-B1.*

An additional important region with QTLs for RSA, GY, and TGW was identified on chromosome 2BL (*RSA_QTLcluster_6#*) where eight RSA QTLs were mapped in our durum RIL populations as well as in three hexaploid wheat mapping populations. Notwithstanding the relatively high significance and *R* ^2^ values in both RIL populations, this QTL region was not highlighted by GWAS, possibly due to (i) unbalanced frequencies of the causal alleles in the Unibo-DP; or (ii) small QTL effects that failed to reach significance. This could also have been the case for *RSA_QTLcluster_12#* on chromosome 4BL and for *RSA_QTLcluster_16#* on chromosome 6AL.

### QTLs for RGA and their associated effects on GY and TGW

A series of RGA QTLs (~30 in total) were identified. The high heritability of this trait (from 0.65 to 0.90) probably facilitated QTL identification. For RGA, biparental linkage mapping, as compared with GWAS, highlighted a higher number of ‘nominal’ QTL effects (six in RILs versus four in Unibo-DP), most probably as a consequence of the higher heritability of RGA in RILs as compared with the panel. From a breeding standpoint, the panel provided a valuable estimate of the effects of QTL alleles across many different genetic backgrounds and provides and overview on their allelic distribution in the elite germplasm, two important aspects for their development via marker-assisted selection. Several criteria were considered for prioritizing the RGA QTLs. The striking co-locations between RGA QTLs and both GY and TGW QTL peaks from multienvironmental trials together with the analysis of the relationships ascertained between RGA and GY effects suggest that *QRGA.ubo-2A.1*, *QRGA.ubo-2A.2*, *QRGA.ubo-2A.3*, *QRGA.ubo-6A.1*, *QRGA.ubo-7A.1*, *QRGA.ubo-7A.4*, and *QRGA.ubo-7B* deserve further characterization, through the development of near-isogenic lines in reference cultivars and/or cumulative/recurrent selection of the appropriate QTL alleles in segregating populations. Mapping populations allowed us to identify three major QTLs for RGA showing additive effects exceeding 10 °, one of which (*QRga.ubo-6A.2*) was cross-validated in the Co×Ld population and the Unibo-DP. These features make *QRGA.ubo-6A.2* a valuable candidate for positional cloning (Salvi and Tuberosa, 2005). The RGA QTLs reported herein for seminal roots can potentially be considered for further development of breeding applications/studies towards the realization (by marker-assisted selection) of the two shallow versus deep root architecture ideotypes schematically depicted in Supplementary Fig. S4.

Out of 30 RGA QTLs, only six were previously described in the wheat germplasm. Thus, 24 novel QTLs for RGA have been identified, including some with large effects such as *QRGA.ubo-6A.2.* From a breeding standpoint, it should be noted that for most of the RGA QTLs identified in the Unibo-DP, the QTL-tagging alleles for narrow rooting were already present at relatively high frequency in the modern elite germplasm (the SNP allele associated with a narrow angle is present in the germplasm with a frequency >0.50 for nine out of 20 QTLs) while the groups of cultivars more directly derived from the local native germplasm showed fixation for the shallow-rooting alleles at several QTLs. Notably, the *QRGA.ubo-6A.2* QTL allele for shallow rooting is contributed by Colosseo, a cultivar with drought susceptibility features, particularly under terminal drought/heat stress (as from the Italian durum wheat national network trial reports, 1995–2005) and known to harbour several chromosome regions directly inherited from Mediterranean tetraploid landraces (Maccaferri *et al.*, 2007). Collectively, our findings suggest a gradual change in RSA from native wheat genetic resources (with shallow and densely rooted RSA) to modern wheat cultivars, the latter being characterized by a deeper rooting system more balanced in density throughout the soil depth as shown by Gioia *et al.* (2015) and Nakhforoosh *et al.* (2015).

### Conclusions and perspectives

A two-pronged approach based on biparental linkage and association mapping allowed the most comprehensive dissection of the QTLome for RSA in durum and bread wheat at the seedling stage while providing valuable information on the associated effects of these QTLs on grain yield of durum wheat across a range of environments differing widely in water availability and productivity. Comparative analysis with previous QTL studies in durum and bread wheat indicated that a large portion of the QTL clusters for RSA traits described herein (~40% for root vigour and 60% specific for RGA) are novel. On the basis of a multienvironment field study, 15 of the 20 QTL clusters for root vigour overlapped with QTL density peaks for grain weight and/or grain yield. Out of the 30 QTLs for RGA, six did not affect other RSA features, hence providing valuable opportunities for fine-tuning RGA independently of other RSA traits. More importantly, nine RGA QTLs also affected grain yield and, in three cases (*QRga.ubo-7B*, *QRga.ubo-6A.1*, and *QRga*.*ubo-7A.4*), the QTLs consistently affected both traits across most environments. These three QTLs are promising candidates for positional cloning which would eventually allow for a more effective manipulation of RGA via marker-assisted selection and/or genome editing. The phenotyping of near-isogenic stocks at these three QTLs would also contribute valuable data for modelling the effects of RSA on GY across environments with different water and nutrient availability (Tardieu and Tuberosa, 2010), ultimately allowing for the fine-tuning of the RSA for a more sustainable wheat production.

## Supplementary data

Supplementary data are available at *JXB* online.

Text S1. QTL analysis methodology in the two recombinant inbred line populations.

Text S2. Genome-wide association (GWAS) analysis methodology in the Unibo-DP durum panel.

Text S3. Phenotypic correlations between seminal root and shoot traits.

Table S1. Correlations among root system architecture traits for the Colosseo×Lloyd and Meridiano×Claudio recombinant inbred line populations.

Table S2. QTLs detected for root system architecture (RSA) traits in the Colosseo×Lloyd mapping population.

Table S3. QTLs detected for root system architecture (RSA) traits in the Meridiano×Claudio mapping population.

Table S4. Detailed results of genome-wide association tests for root system architecture (RSA) traits in the Unibo-DP durum panel.

Table S5. Allelic distribution for root growth angle (RGA) QTL-tagging SNPs in the five Unibo-DP durum panel subgroups as defined by population structure analysis. Accessions listed by RGA.

Table S6. Allelic distribution for root growth angle (RGA) QTL-tagging SNPs in the five Unibo-DP durum panel subgroups as defined by population structure analysis. Accessions listed by population structure.

Table S7. Effect on grain yield (GY) and thousand grain weight (TGW) of GWAS QTLs for root growth angle (RGA) in the Unibo-DP panel based on 15 field trials carried out in the Mediterranean Basin.

Fig. S1. Frequency distribution of root system architecture (RSA) traits in the recombinant inbred populations Colosseo×Lloyd and Meridiano×Claudio.

Fig. S2. Root system architecture (RSA) QTLs in tetraploid wheat projected onto an SNP-based tetraploid consensus map.

Fig. S3. Root system architecture (RSA) QTLs, grain yield (GY), and thousand grain weight (TKW) in tetraploid wheat projected onto an SNP-based tetraploid consensus map.

Fig. S4. Schematic ideotypes of wheat with shallow and deep root system architecture as a consequence of different seminal root growth angle (RGA) QTLs contributing towards the ideotypes by marker-assisted selection.

Supplementary Data

## References

[CIT0001] Acuna TLB Rebetzke GJ He X Maynol E Wade LJ 2014 Mapping quantitative trait loci associated with root penetration ability of wheat in contrasting environments. Molecular Breeding 34, 631–642.

[CIT0002] Acuna TLB Wade LJ 2012 Genotype × environment interactions for root depth of wheat. Field Crops Research 137, 117–125.

[CIT0003] Arai-Sanoh Y Takai T Yoshinaga S Nakano H Kojima M Sakakibara H Kondo M Uga Y 2014 Deep rooting conferred by *DEEPER ROOTING 1* enhances rice yield in paddy fields. Scientific Reports 4, 5563. 24988911 10.1038/srep05563 PMC4080195

[CIT0004] Atkinson JA Wingen LU Griffiths M 2015 Phenotyping pipeline reveals major seedling root growth QTL in hexaploid wheat. Journal of Experimental Botany 66, 2283–2292. 25740921 10.1093/jxb/erv006 PMC4407652

[CIT0005] Bai C Liang Y Hawkesford MJ 2013 Identification of QTLs associated with seedling root traits and their correlation with plant height in wheat. Journal of Experimental Botany 64, 1745–1753. 23564959 10.1093/jxb/ert041 PMC3617839

[CIT0006] Belford RK Klepper B Rickman RW 1987 Studies of intact shoot–root systems of field-grown winter wheat. 2. Root and shoot developmental pattern as related to nitrogen-fertilizer. Agronomy Journal 79, 310–319.

[CIT0007] Bishopp A Lynch J 2015 The hidden half of crop yields Nature Plants 1, 15117. 10.1038/nplants.2015.117 27250548

[CIT0008] Blum A 2009 Effective use of water (EUW) and not water-use efficiency (WUE) is the target of crop yield improvement under drought stress. Field Crops Research 112, 119–123.

[CIT0009] Borrell AK Mullet JE George-Jaeggli B van Oosterom EJ Hammer GL Klein PE Jordan DR 2014 Drought adaptation of stay-green sorghum is associated with canopy development, leaf anatomy, root growth, and water uptake. Journal of Experimental Botany 65, 6251–6263. 25381433 10.1093/jxb/eru232 PMC4223986

[CIT0010] Canè MA Maccaferri M Nazemi G Salvi S Francia R Colalongo C Tuberosa R 2014 Association mapping for root architectural traits in durum wheat seedlings as related to agronomic performance. Molecular Breeding 34, 1629–1645. 25506257 10.1007/s11032-014-0177-1 PMC4257993

[CIT0011] Cao P Ren Y Zhang K 2014 Further genetic analysis of a major quantitative trait locus controlling root length and related traits in common wheat. Molecular Breeding 33, 975–985.

[CIT0012] Chochois V Vogel JP Rebetzke GJ Watt M 2015 Variation in adult plant phenotypes and partitioning among seed and stem-borne roots across *Brachypodium distachyon* accessions to exploit in breeding cereals for well-watered and drought environments. Plant Physiology 168, 953–967. 25975834 10.1104/pp.15.00095 PMC4741322

[CIT0013] Christopher J Christopher M Jennings R Jones S Fletcher S Borrell A Manschadi AM Jordan D Mace E Hammer G 2013 QTL for root angle and number in a population developed from bread wheats (*Triticum aestivum*) with contrasting adaptation to water-limited environments. Theoretical and Applied Genetics 126, 1563–1574. 23525632 10.1007/s00122-013-2074-0

[CIT0014] Courtois B Audebert A Dardou A 2013 Genome-wide association mapping of root traits in a japonica rice panel. PLoS One 8, e78037. 24223758 10.1371/journal.pone.0078037 PMC3818351

[CIT0015] Collins NC Tardieu F Tuberosa R 2008 Quantitative trait loci and crop performance under abiotic stress: where do we stand? Plant Physiology 147, 469–486. 18524878 10.1104/pp.108.118117 PMC2409033

[CIT0016] Delhaize E Rathjen TM Cavanagh CR 2015 The genetics of rhizosheath size in a multiparent mapping population of wheat. Journal of Experimental Botany 66, 4527–4536. 25969556 10.1093/jxb/erv223 PMC4507764

[CIT0017] de Dorlodot S Forster B Price A Tuberosa R Draye X 2007 Root system architecture: opportunities and constraints for genetic improvement of crops. Trends in Plant Science 12, 474–481. 17822944 10.1016/j.tplants.2007.08.012

[CIT0018] De Vita P Mastrangelo AM Matteu L Mazzucotelli E Virzì N Palumbo M Lo Storto M Rizza F Cattivelli L 2010 Genetic improvement effects on yield stability in durum wheat genotypes grown in Italy. Fields Crops Research 119, 68–77.

[CIT0019] Ehdaie B Maheepala DC Bektaş H Waines JG 2014 Phenotyping and genetic analysis of root and shoot traits of recombinant inbred lines of bread wheat under well-watered conditions. Journal of Crop Improvement 28, 834–851.

[CIT0020] Esau K 1965 Plant anatomy , 2nd edn. New York: John Wiley.

[CIT0021] Fleury D Jefferies S Kuchel H Langridge P 2010 Genetic and genomic tools to improve drought tolerance in wheat. Journal of Experimental Botany 61, 3211–3222. 20525798 10.1093/jxb/erq152

[CIT0022] Gioia T Nagel KA Beleggia R Fragasso M Ficco DBM Pieruschka R De Vita P Fiorani F Papa R 2015 Impact of domestication on the phenotypic architecture of durum wheat under contrasting nitrogen fertilization. Journal of Experimental Botany 66, 5519–5530. 26071535 10.1093/jxb/erv289

[CIT0023] Guo Y Kong F-m Xu Y-f Zhao Y Liang X Wang Y-y An D-g Li S 2012 QTL mapping for seedling traits in wheat grown under varying concentrations of N, P and K nutrients. Theoretical and Applied Genetics 124, 851–865. 22089330 10.1007/s00122-011-1749-7

[CIT0024] Hamada A Nitta M Nasuda S Kato K Fujita M Matsunaka H Okumoto Y 2012 Novel QTLs for growth angle of seminal roots in wheat (*Triticum aestivum* L.). Plant and Soil 354, 395–405.

[CIT0025] Hawkesford MJ 2014 Reducing the reliance on nitrogen fertilizer for wheat production. Journal of Cereal Science 59, 276–283. 24882935 10.1016/j.jcs.2013.12.001 PMC4026125

[CIT0026] He X Fang J Li J 2014 A genotypic difference in primary root length is associated with the inhibitory role of transforming growth factor-beta receptor-interacting protein-1 on root meristem size in wheat. The Plant Journal 77, 931–943. 24467344 10.1111/tpj.12449

[CIT0027] Hochholdinger F Tuberosa R 2009 Genetic and genomic dissection of maize root development and architecture. Current Opinion in Plant Biology 12, 172–177. 19157956 10.1016/j.pbi.2008.12.002

[CIT0028] Howell T Hale I Jankuloski L Bonafede M Gilbert M Dubcovsky J 2014 Mapping a region within the 1RS.1BL translocation in common wheat affecting grain yield and canopy water status. Theoretical and Applied Genetics 127, 2695–2709. 25322723 10.1007/s00122-014-2408-6 PMC4236633

[CIT0029] Hund A Reimer R Messmer R 2011 A consensus map of QTLs controlling the root length of maize. Plant and Soil 344, 143–158.

[CIT0030] Kato Y Abe J Kamoshita A Yamagishi J 2006 Genotypic variation in root growth angle in rice (*Oryza sativa* L.) and its association with deep root development in upland fields with different water regimes. Plant and Soil 287, 117–129.

[CIT0031] Kao CH Zeng ZB Teasdale RD 1999 Multiple interval mapping for quantitative trait loci. Genetics 152, 1203–1216. 10388834 10.1093/genetics/152.3.1203 PMC1460657

[CIT0032] King J Gay A Sylvester-Bradley R Bingham I Foulkes J Gregory P Robinson D 2003 Modelling cereal root systems for water and nitrogen capture: towards an economic optimum. Annals of Botany 91, 383–390. 12547691 10.1093/aob/mcg033 PMC4244970

[CIT0033] Kitomi Y Kanno N Kawai S Mizubayashi T Fukuoka S Uga Y 2015 QTLs underlying natural variation of root growth angle among rice cultivars with the same functional allele of *DEEPER ROOTING 1* . Rice 8, 16. 25844121 10.1186/s12284-015-0049-2 PMC4385264

[CIT0034] Kubo K Elouafi L Watanabe N Nachit MM Inagaki MN Iwama K Jitsuyama Y 2007 Quantitative trait loci for soil-penetrating ability of roots in durum wheat. Plant Breeding 126, 375–378.

[CIT0035] Landi P Giuliani S Salvi S Ferri M Tuberosa R Sanguineti MC 2010 Characterization of *root-yield-1.06*, a major constitutive QTL for root and agronomic traits in maize across water regimes. Journal of Experimental Botany 61, 3553–3562. 20627896 10.1093/jxb/erq192

[CIT0036] Langridge P Reynolds MP 2015 Genomic tools to assist breeding for drought tolerance. Current Opinion in Biotechnology 32, 130–135. 25531270 10.1016/j.copbio.2014.11.027

[CIT0037] Laperche A Devienne-Barret F Maury O Le Gouis J Ney B 2006 A simplified conceptual model of carbon/nitrogen functioning for QTL analysis of winter wheat adaptation to nitrogen deficiency. Theoretical and Applied Genetics 113, 1131–1146. 16909280 10.1007/s00122-006-0373-4

[CIT0038] Letta T Maccaferri M Badebo A Ammar K Ricci A Crossa J Tuberosa R 2013 Searching for novel sources of field resistance to Ug99 and Ethiopian stem rust races in durum wheat via association mapping. Theoretical and Applied Genetics 126, 1237–1256. 23429902 10.1007/s00122-013-2050-8

[CIT0039] Li P Chen F Cai H Liu J Pan Q Liu Z Gu R Mi G Zhang F Yuan L 2015 A genetic relationship between nitrogen use efficiency and seedling root traits in maize as revealed by QTL analysis. Journal of Experimental Botany 66, 3175–3188. 25873660 10.1093/jxb/erv127 PMC4449538

[CIT0040] Liu X Li R Chang X Jing R 2013 Mapping QTLs for seedling root traits in a doubled haploid wheat population under different water regimes. Euphytica 189, 51–66.

[CIT0041] Lobet G Pages L Draye X 2011 A novel image-analysis toolbox enabling quantitative analysis of root system architecture. Plant Physiology 157, 29–39. 21771915 10.1104/pp.111.179895 PMC3165877

[CIT0042] Lopes MS Rebetzke GJ Reynolds M 2014 Integration of phenotyping and genetic platforms for a better understanding of wheat performance under drought. Journal of Experimental Botany 65, 6167–6177. 25246446 10.1093/jxb/eru384

[CIT0043] Lopez-Castaneda C Richards RA Farquhar GD 1995 Variation in early vigor between wheat and barley. Crop Science 35, 472–479.

[CIT0044] Ludlow MM Muchow RC 1990 A critical evaluation of traits for improving crop yields in water-limited environments. Advances in Agronomy 43, 107–153.

[CIT0045] Lynch JP 2013 Steep, cheap and deep: an ideotype to optimize water and N acquisition by maize root systems. Annals of Botany 112, 347–357. 23328767 10.1093/aob/mcs293 PMC3698384

[CIT0046] Maccaferri M Canè MA Sanguineti MC 2014 A consensus framework map of durum wheat (*Triticum durum* Desf.) suitable for linkage disequilibrium analysis and genome-wide association mapping. BMC Genomics 15, 873. 25293821 10.1186/1471-2164-15-873 PMC4287192

[CIT0047] Maccaferri M Mantovani P Tuberosa R Deambrogio E Giuliani S Demontis A Massi A Sanguineti MC 2008 A major QTL for durable leaf rust resistance widely exploited in durum wheat breeding programs maps on the distal region of chromosome arm 7BL. Theoretical and Applied Genetics 117, 1225–1240. 18712342 10.1007/s00122-008-0857-5

[CIT0048] Maccaferri M Ricci A Salvi S 2015 A high-density, SNP-based consensus map of tetraploid wheat as a bridge to integrate durum and bread wheat genomics and breeding. Plant Biotechnology Journal 13, 648–663. 25424506 10.1111/pbi.12288

[CIT0049] Maccaferri M Sanguineti MC Demontis A 2011 Association mapping in durum wheat grown across a broad range of water regimes. Journal of Experimental Botany 62, 409–438. 21041372 10.1093/jxb/erq287

[CIT0050] Maccaferri M Sanguineti MC Noli E Tuberosa R 2005 Population structure and long-range linkage disequilibrium in a durum wheat elite collection. Molecular Breeding 15, 271–289.

[CIT0051] Maccaferri M Sanguineti MC Xie C Smith JSC Tuberosa R 2007 Relationships among durum wheat accessions. II. A comparison of molecular and pedigree information. Genome 50, 385–399. 17546097 10.1139/g07-017

[CIT0052] Mace ES Singh V Van Oosterom EJ Hammer GL Hunt CH Jordan DR 2012 QTL for nodal root angle in sorghum (*Sorghum bicolor* L. Moench) co-locate with QTL for traits associated with drought adaptation. Theoretical and Applied Genetics 124, 97–109. 21938475 10.1007/s00122-011-1690-9

[CIT0053] Mackay TFC 1996 The nature of quantitative genetic variation revisited: Lessons from Drosophila bristles. Bioessays 18, 113–121. 8851044 10.1002/bies.950180207

[CIT0054] Manschadi AM Christopher JT Hammer GL Devoil P 2010 Experimental and modelling studies of drought-adaptive root architectural traits in wheat (*Triticum aestivum* L.). Plant Biosystems 144, 458–462.

[CIT0055] Manschadi AM Manske GGB Vlek PLG 2013 Root architecture and resource acquisition—wheat as a model plant. In: Eshel A Beeckman T, eds. Plant roots–the hidden half , 4th edn. Boca Raton, FL: CRC Press, 1–22.

[CIT0056] Manske GGB Vlek PLG 2002 Root architecture—wheat as a model plant . In: Waisel Y Eshel A Kafkafi U, eds. Plant roots–the hidden half , 3rd edn. Boca Raton, FL: CRC Press, 249–259.

[CIT0057] Mantovani P Maccaferri M Sanguineti MC 2008 An integrated DArT-SSR linkage map of durum wheat. Molecular Breeding 22, 629–648.

[CIT0058] Meister R Rajani MS Ruzicka D Schachtman DP 2014 Challenges of modifying root traits in crops for agriculture. Trends in Plant Science 19, 779–788. 25239776 10.1016/j.tplants.2014.08.005

[CIT0059] Mickelbart MV Hasegawa PM Bailey-Serres J 2015 Genetic mechanisms of abiotic stress tolerance that translate to crop yield stability. Nature Reviews Genetics 16, 237–251. 10.1038/nrg3901 25752530

[CIT0060] Miguel MA Postma JA Lynch JP 2015 Phene synergism between root hair length and basal root growth angle for phosphorus acquisition. Plant Physiology 167, 1430–1439. 25699587 10.1104/pp.15.00145 PMC4378183

[CIT0061] Milner SG Maccaferri M Huang BE Mantovani P Massi A Frascaroli E Tuberosa R Salvi S 2015 A multiparental cross population for mapping QTL for agronomic traits in durum wheat (*Triticum turgidum* ssp. *durum*). Plant Biotechnology Journal doi: 10.1111/pbi.12424. 10.1111/pbi.12424 PMC11388855 26132599

[CIT0062] Muñoz-Amatriaín M Cuesta-Marcos A Endelman JB 2014 The USDA barley core collection: genetic diversity, population structure, and potential for genome-wide association studies. PLoS One 9, e94688. 24732668 10.1371/journal.pone.0094688 PMC3986206

[CIT0063] Nakhforoosh A Grausgruber H Kaul H-P Bodner G 2015 Dissection of drought response of modern and underutilized wheat varieties according to Passioura’s yield-water framework. Frontiers in Plant Science 6, 570. 26257766 10.3389/fpls.2015.00570 PMC4511830

[CIT0064] Oyanagi A Nakamoto T Wada M 1993 Relationship between root-growth angle of seedlings and vertical-distribution of roots in the field in wheat cultivars. Japanese Journal of Crop Science 62, 565–570.

[CIT0065] Paez-Garcia A Motes CM Scheible WR Chen R Blancaflor EB Monteros MJ 2015 Root traits and phenotyping strategies for plant improvement. Plants 4, 334–355. 10.3390/plants4020334 PMC4844329 27135332

[CIT0066] Petrarulo M Marone D Ferragonio P Cattivelli L Rubiales D De Vita P Mastrangelo AM 2015 Genetic analysis of root morphological traits in wheat. Molecular Genetics and Genomics 290, 785–806. 25416422 10.1007/s00438-014-0957-7

[CIT0067] Pinto RS Reynolds MP 2015 Common genetic basis for canopy temperature depression under heat and drought stress associated with optimized root distribution in bread wheat. Theoretical and Applied Genetics 128, 575–585. 25707766 10.1007/s00122-015-2453-9 PMC4361760

[CIT0068] Ren Y He X Liu D Li J Zhao X Li B Tong Y Zhang A Li Z 2012 Major quantitative trait loci for seminal root morphology of wheat seedlings. Molecular Breeding 30, 139–148.

[CIT0069] Reynolds M Tuberosa R 2008 Translational research impacting on crop productivity in drought-prone environments. Current Opinion in Plant Biology 11, 171–179. 18329330 10.1016/j.pbi.2008.02.005

[CIT0070] Reynolds MP Pierre CS Saad ASI Vargas M Condon AG 2007 Evaluating potential genetic gains in wheat associated with stress-adaptive trait expression in elite genetic resources under drought and heat stress. Crop Science 47, S-172–S-189.

[CIT0071] Richard CAI Hickey LT Fletcher S Jennings R Chenu K Christopher JT 2015 High-throughput phenotyping of seminal root traits in wheat. Plant Methods 11, 13. 25750658 10.1186/s13007-015-0055-9 PMC4351910

[CIT0072] Richards RA Passioura JB 1989 A breeding program to reduce the diameter of the major xylem vessel in the seminal roots of wheat and its effect on grain yield in rain-fed environments. Australian Journal of Agricultural Research 40, 943–950.

[CIT0073] Salvi S Tuberosa R 2015 The crop QTLome comes of age. Current Opinion in Biotechnology 32, 179–185. 25614069 10.1016/j.copbio.2015.01.001

[CIT0074] Sanguineti MC Li S Maccaferri M Corneti S Rotondo F Chiari T Tuberosa R 2007 Genetic dissection of seminal root architecture in elite durum wheat germplasm. Annals of Applied Biology 151, 291–305.

[CIT0075] Sharma S DeMason DA Ehdaie B Lukaszewski AJ Waines JG 2010 Dosage effect of the short arm of chromosome 1 of rye on root morphology and anatomy in bread wheat. Journal of Experimental Botany 61, 2623–2633. 20444906 10.1093/jxb/erq097 PMC2882260

[CIT0076] Sosnowski O Charcosset A Joets J 2012 BioMercator V3: an upgrade of genetic map compilation and quantitative trait loci meta-analysis algorithms. Bioinformatics 28, 2082–2083. 22661647 10.1093/bioinformatics/bts313 PMC3400960

[CIT0077] Steele KA Price AH Witcombe JR Shrestha R Singh BN Gibbons JM Virk DS 2013 QTLs associated with root traits increase yield in upland rice when transferred through marker-assisted selection. Theoretical and Applied Genetics 126, 101–108. 22968512 10.1007/s00122-012-1963-y

[CIT0078] Tardieu F Tuberosa R 2010 Dissection and modelling of abiotic stress tolerance in plants. Current Opinion in Plant Biology 13, 206–212. 20097596 10.1016/j.pbi.2009.12.012

[CIT0079] Trachsel S Kaeppler S Brown K Lynch J 2011 Shovelomics: high throughput phenotyping of maize (*Zea mays* L.) root architecture in the field. Plant and Soil 341, 75–87.

[CIT0080] Tuberosa R 2012 Phenotyping for drought tolerance of crops in the genomics era. Frontiers in Physiology 3, 347, 1–26. 23049510 10.3389/fphys.2012.00347 PMC3446691

[CIT0081] Tuberosa R Pozniak C 2014 Durum wheat genomics comes of age. Molecular Breeding 34, 1527–1530.

[CIT0082] Tuberosa R Salvi S Sanguineti MC Landi P Maccaferri M Conti S 2002 Mapping QTLs regulating morpho-physiological traits and yield: case studies, shortcomings and perspectives in drought-stressed maize. Annals of Botany 89, 941–963. 12102519 10.1093/aob/mcf134 PMC4233811

[CIT0083] Tuberosa R Salvi S Sanguineti MC Maccaferri M Giuliani S Landi P 2003 Searching for quantitative trait loci controlling root traits in maize: a critical appraisal. Plant and Soil 255, 35–54.

[CIT0084] Uga Y Kitomi Y Ishikawa S Yano M 2015 Genetic improvement for root growth angle to enhance crop production. Breeding Science 65, 111–119. 26069440 10.1270/jsbbs.65.111 PMC4430504

[CIT0085] Uga Y Sugimoto K Ogawa S 2013 Control of root system architecture by *DEEPER ROOTING 1* increases rice yield under drought conditions. Nature Genetics 45, 1097. 23913002 10.1038/ng.2725

[CIT0086] Wang S Wong D Forrest K 2014 Characterization of polyploid wheat genomic diversity using a high-density 90 000 single nucleotide polymorphism array. Plant Biotechnology Journal 12, 787–796. 24646323 10.1111/pbi.12183 PMC4265271

[CIT0087] Wasson AP Rebetzke GJ Kirkegaard JA Christopher J Richards RA Watt M 2014 Soil coring at multiple field environments can directly quantify variation in deep root traits to select wheat genotypes for breeding. Journal of Experimental Botany 65, 6231–6249. 24963000 10.1093/jxb/eru250 PMC4223987

[CIT0088] Wasson AP Richards RA Chatrath R Misra SC Prasad SV Rebetzke GJ Kirkegaard JA Christopher J Watt M 2012 Traits and selection strategies to improve root systems and water uptake in water-limited wheat crops. Journal of Experimental Botany 63, 3485–3498. 22553286 10.1093/jxb/ers111

[CIT0089] Watt M Moosavi S Cunningham SC Kirkegaard JA Rebetzke GJ Richards RA 2013 A rapid, controlled-environment seedling root screen for wheat correlates well with rooting depths at vegetative, but not reproductive, stages at two field sites. Annals of Botany 112, 447–455. 23821620 10.1093/aob/mct122 PMC3698392

