# Supplementary material for: Prioritizing quantitative trait loci for root system architecture in tetraploid wheat
Source: J Exp Bot. 2016 Feb 13;67(4):1161–78. doi: 10.1093/jxb/erw039 (PMC4753857; doi:10.1093/jxb/erw039)
Supplement: Supplementary Data [file supp_67_4_1161__index.html]

Prioritizing quantitative trait loci for root system architecture in tetraploid wheat — Supplementary Data 

# Prioritizing quantitative trait loci for root system architecture in tetraploid wheat

## Supplementary Data

Data files

- Supplementary\_figures\_S1\_S4.pdf - Supplementary Data
- supplementary\_methods\_Tables\_S1\_S3.docx - Supplementary Data
- supplementary\_table\_S4.xlsx - Supplementary Data
- supplementary\_table\_S5\_S6.xlsx - Supplementary Data
- supplementary\_table\_S7.xlsx - Supplementary Data
