# Supplementary material for: Prioritizing quantitative trait loci for root system architecture in tetraploid wheat
Source: J Exp Bot. 2016 Feb 13;67(4):1161–78. doi: 10.1093/jxb/erw039 (PMC4753857; doi:10.1093/jxb/erw039)
Supplement: Supplementary Data [file supp_erw039_Supplementary_figures_S1_S4.pdf]

Title: **Prioritizing QTLs for root system architecture in tetraploid wheat**

Supplementary Figure S1-S4.

Authors: Marco Maccaferri*[,1], Walid El-Feki[1,2], Nazemi Ghasemali[1,3], Silvio Salvi[1], Maria Angela Canè M[1], Chiara Colalongo[1], Sandra Stefanelli[1], Roberto Tuberosa[1]

[1]Department of Agricultural Sciences, University of Bologna, 40127 Bologna, Italy
[2]Department of Crop Sciences, Faculty of Agriculture, Alexandria University, 23714 Alexandria, Egypt
[3]Department of Agriculture, Hajiabad Branch, Islamic Azad University, 21100 Hajiabad, Iran
* To whom correspondence should be addressed: marco.maccaferri@unibo.it

Supplementary Figure 1. frequency distribution of root seminal architecture (RSA) traits measured in the recombinant inbred populations Colosseo×Lloyd and Meridiano×Claudio.

Distributions are reported for the most relevant RSA traits.

List of RSA acronyms:

Root seminal architecture traits

TRN, total root number

Rt6, presence of the 6th asymmetric seminal root

PRL, primary root length

TRL, total root length

ARL, average root length

LRN, lateral root number

RGA, root growth angle

SL, shoot length

SDW, shoot dry weight

RDW, root dry weight

RSR, root to shoot ratio

iTGW, thousand grain weight of seeds used in the RSA experiment

Field traits

GY, grain yield

TGW, thousand grain weight in field trials

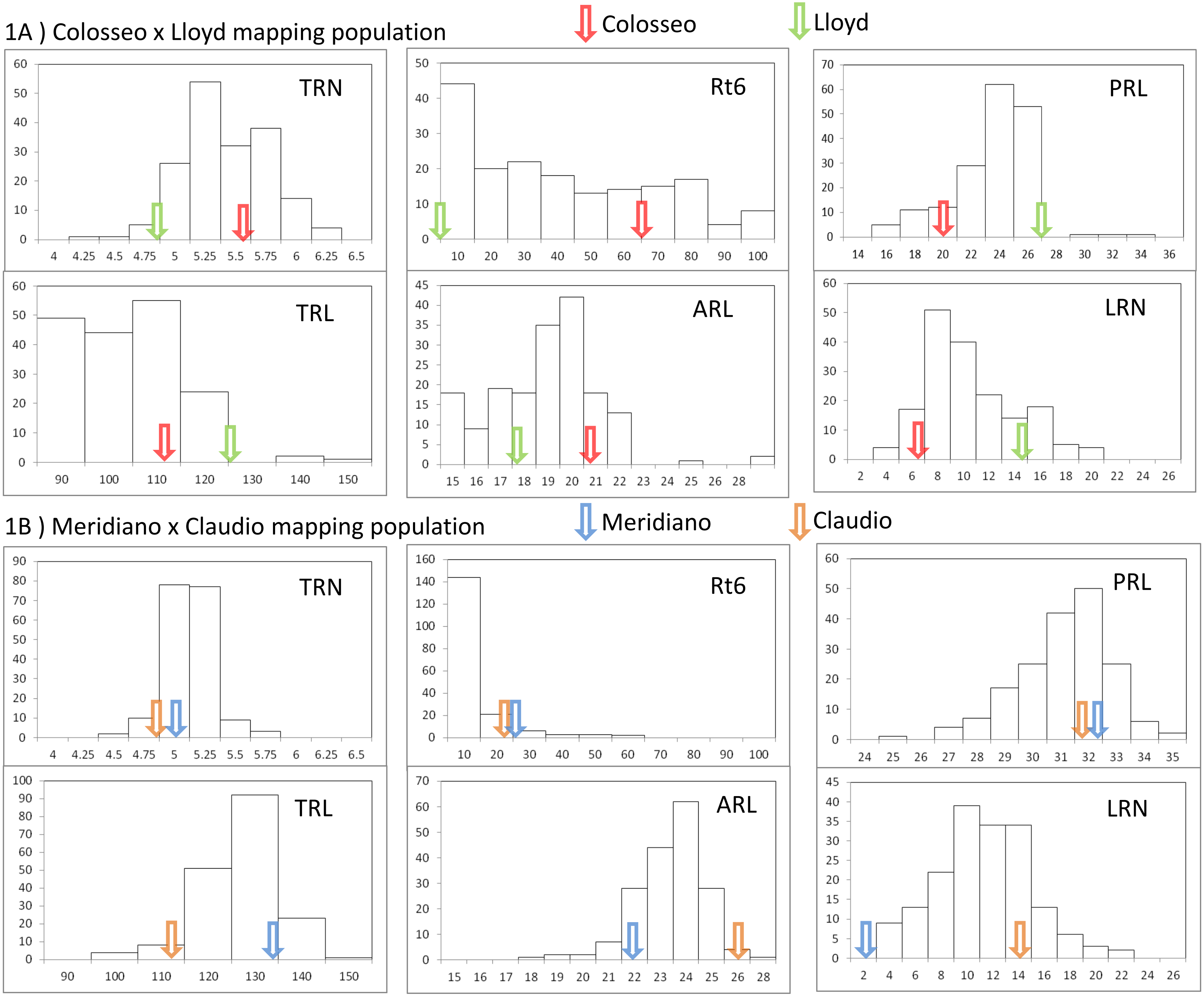
1A) Colosseo x Lloyd mapping population
Colosseo
Lloyd
TRN
Rt6
PRL
TRL
ARL
LRN
1B) Meridiano x Claudio mapping population
Meridiano
Claudio
TRN
Rt6
PRL
TRL
ARL
LRN

2A) Colosseo x Lloyd mapping population

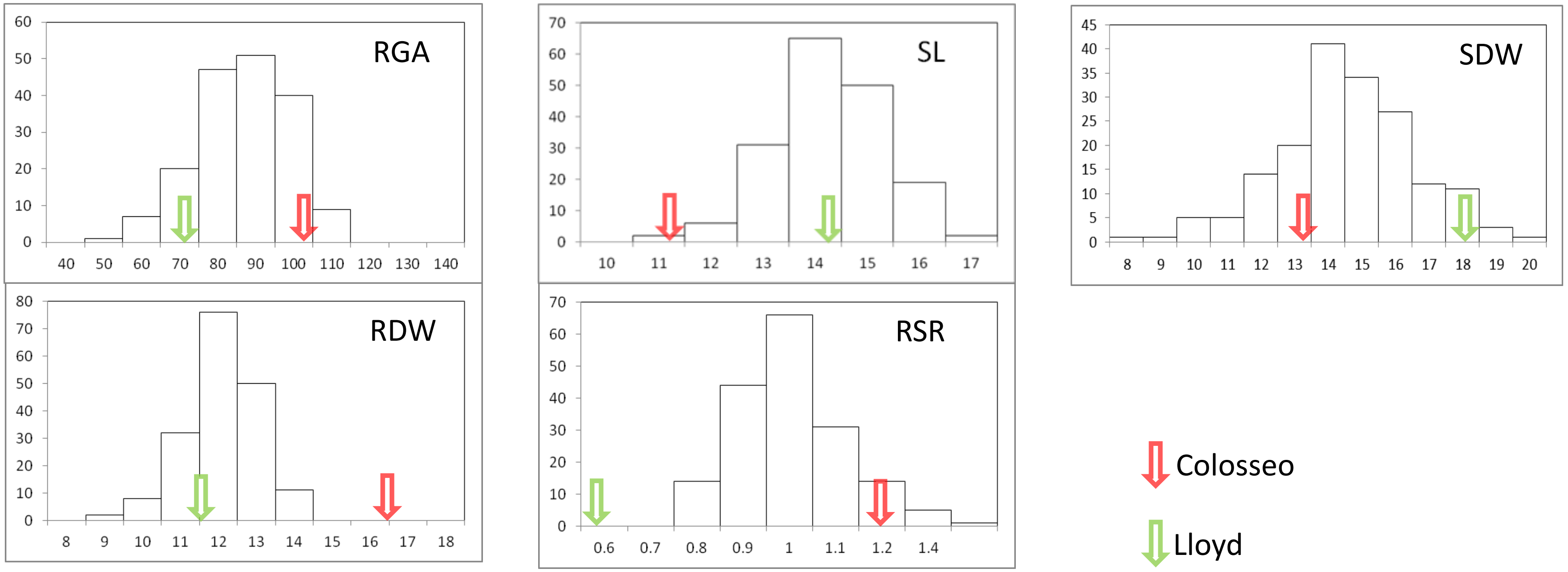

2B) Meridiano x Claudio mapping population

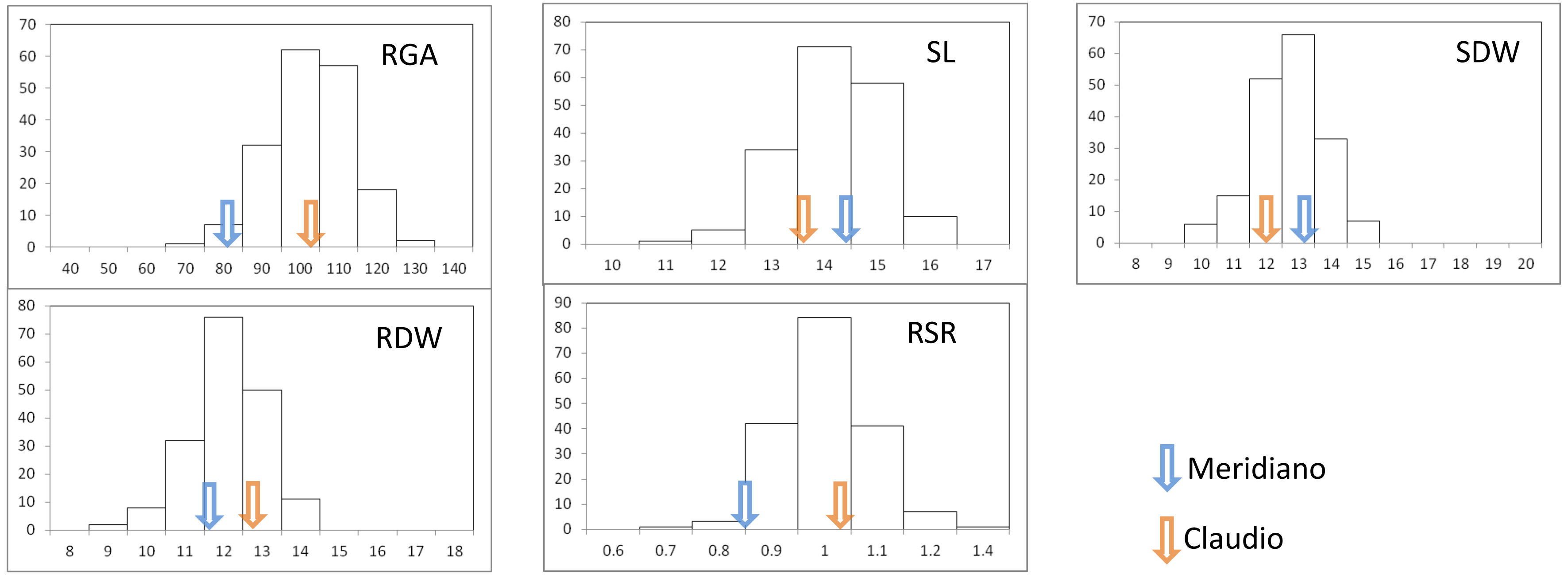

Supplementary Figure S2. Schematic representation of root system architecture (RSA) QTLs in tetraploid wheat projected onto an SNP-based tetraploid consensus map.

Root system architecture (RSA) QTLs from two durum wheat mapping population, the association mapping panel (Durum Panel, DP) and RSA QTLs from previously published studies in wheat are projected.

The consensus tetraploid wheat map has been published in Maccaferri *et al*. (2015).

QTLs from Colosseo × Lloyd (CxL) and Meridiano × Claudio (MxC) are reported on the left of chromosome bars while association mapping QTLs (AM-QTLs) are reported on the right. QTLs for root length and number are reported as blue-filled bars, seminal root angle (RGA) QTLs are reported in orange, shoot-related QTLs in green, grain weigth (GWT) QTLs in red.

QTLs from RIL populations with R^2 < 10% are reported as thin half-tone coloured bars, while QTLs with R^2 > 10% are reported as tick full-tone bars.
The parental allele with a positive associated effect is reported as round-bracketing using the acronyms: **Co**, Colosseo; **Ld**, Lloyd; **Me**, Meridiano; **Cl**, Claudio.

QTLs from association mapping (GWAS-QTLs, reported as DP QTLs) have been reported together with their significance level: **, marker-wise significance *P* 0.01 (-LOG*P*=2); ***, marker-wise significance, *P* 0.001 (-LOG*P*=3=; ****, experiment-wise significance, *P* 0.05 (-LOG*P*=4).

root system architecture (RSA) QTLs from previously published studies in wheat have been projected on the consensus map and reported as black-filled confidence interval bars. QTL references are reported as round-brecketed alpha-numeric acronyms using **Tt** for Triticum turgidum QTLs and **Ta** for Triticum aestivum QTLs.

Syntenic intervals of rice deep rooting and root growth angle QTLs have been projected and reported on the extreme-right of chromosome bars as orange-filled bars using the **Os** acronym.

Major RSA QTL-clusters based on concomitant co-location of RSA QTL confidence intervals from multiple wheat mapping stocks are reported as grey-banded intervals.

RSA trait acronyms: TRN, Total root number; R6th, Presence of the 6th asymmetric seminal root; PRL, primary root length; TRL, total root length; ARL, average root length; LRN, lateral root number; RGA, root growth angle; RDW, root dry weight; SDW, shoot dry weight; SL, shoot length; RSR, root-to-shoot ratio; RS, root surface; RD, root diameter; RV, root volume; GY, grain yield; iTGW, initial thousand grain weight of seeds used in the RSA experiment; TGW, thousand grain weight in field trials

References for projected root system architecture QTLs from previous studies in wheat

Code	Reference	Population	Species
Tt1	Petrarulo et al. (2015) Mol Genet Genomics 290:785-806.	Creso x Pedroso	Tetraploid wheat (Tt)
Ta2	Cao et al. (2014)	Xiaoyan 54 x Jing 411	Hexaploid wheat (Ta)
Ta3	Christopher et al. (2013) Theor Appl Genet 126:1563-1574.	Seri x Hartog	Hexaploid wheat (Ta)
Ta4	Atkinson et al. (2015) J Exp Bot 66:2283-2292.	Savannah x Rialto	Hexaploid wheat (Ta)
Ta5	Bai et al. (2013) J Exp Bot 64:1745-1753.	Avalon x cadenza	Hexaploid wheat (Ta)
Ta6	Liu et al. (2013) Euphytica 189:51-	Hanxuan 10 and Lumai 14	Hexaploid wheat (Ta)
Ta7	Guo et al. (2012)	Chuan 35050 x Shannong 483	Hexaploid wheat (Ta)
Ta8	Hamada et al. (2012)	U24 x AyahiKari	Hexaploid wheat (Ta)
Ta9	Ren et al. (2012)	Xiaoyan 54 x Jing 411	Hexaploid wheat (Ta)
Ta10	Laperche et al. (2006) Theor Appl Genet 113:1131-1146.	Arche x Recital	Hexaploid wheat (Ta)
Tt11	Kubo et al. (2007)	Jennah Khetifa x Cham1	Tetraploid wheat (Ta)
Ta12	An et al. (2006)	Hanxuan 10 x Lumai 14	Hexaploid wheat (Tt)

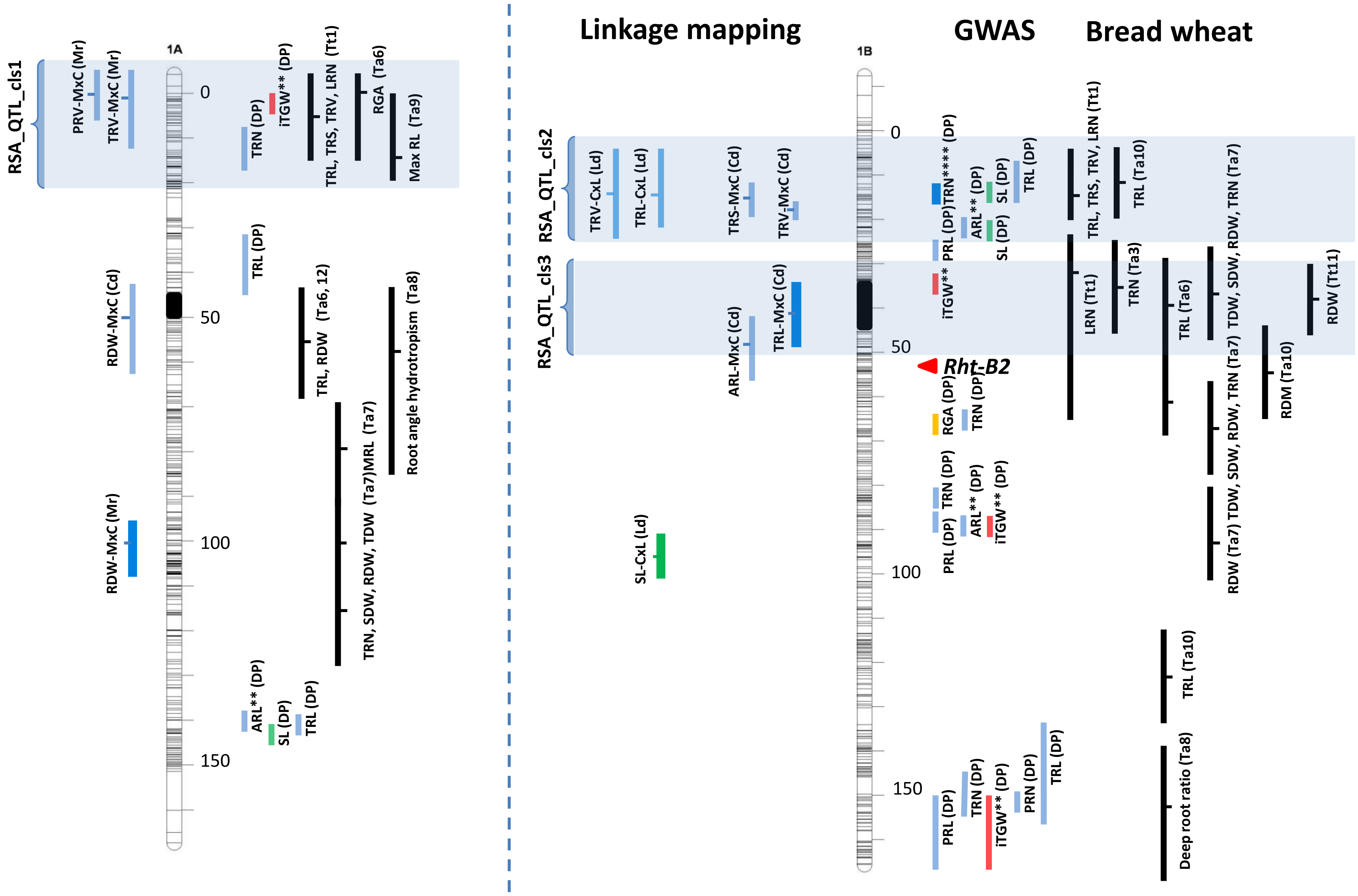

Linkage mapping
GWAS
Bread wheat
1A
1B
RSA_QTL_cls1
RSA_QTL_cls2
RSA_QTL_cls3
0
50
100
150
PRV-MxC (Mr)
TRV-MxC (Mr)
TRN (DP)
iTGW** (DP)
TRL, TRS, TRV, LRN (Tt1)
RGA (Ta6)
Max RL (Ta9)
TRL (DP)
RDW-MxC (Cd)
TRL, RDW (Ta6, 12)
Root angle hydrotropism (Ta8)
TRN, SDW, RDW, TDW (Ta7)MRL (Ta7)
RDW-MxC (Mr)
ARL** (DP)
SL (DP)
TRL (DP)
TRV-CxL (Ld)
TRL-CxL (Ld)
TRS-MxC (Cd)
TRV-MxC (Cd)
ARL-MxC (Cd)
TRL-MxC (Cd)
SL-CxL (Ld)
TRN**** (DP)
PRL (DP)
ARL** (DP)
SL (DP)
TRL (DP)
iTGW** (DP)
Rht-B2
RGA (DP)
TRN (DP)
TRN (DP)
PRL (DP)
ARL** (DP)
iTGW** (DP)
PRL (DP)
TRN (DP)
iTGW** (DP)
PRN (DP)
TRL (DP)
TRL, TRS, TRV, LRN (Tt1)
TRL (Ta10)
LRN (Tt1)
TRN (Ta3)
TRL (Ta6)
TDW, SDW, RDW, TRN (Ta7)
RDW (Tt11)
RDM (Ta10)
RDW, TRN (Ta7)
RDW (Ta7) TDW, SDW, RDW, TRN (Ta7)
TRL (Ta10)
Deep root ratio (Ta8)

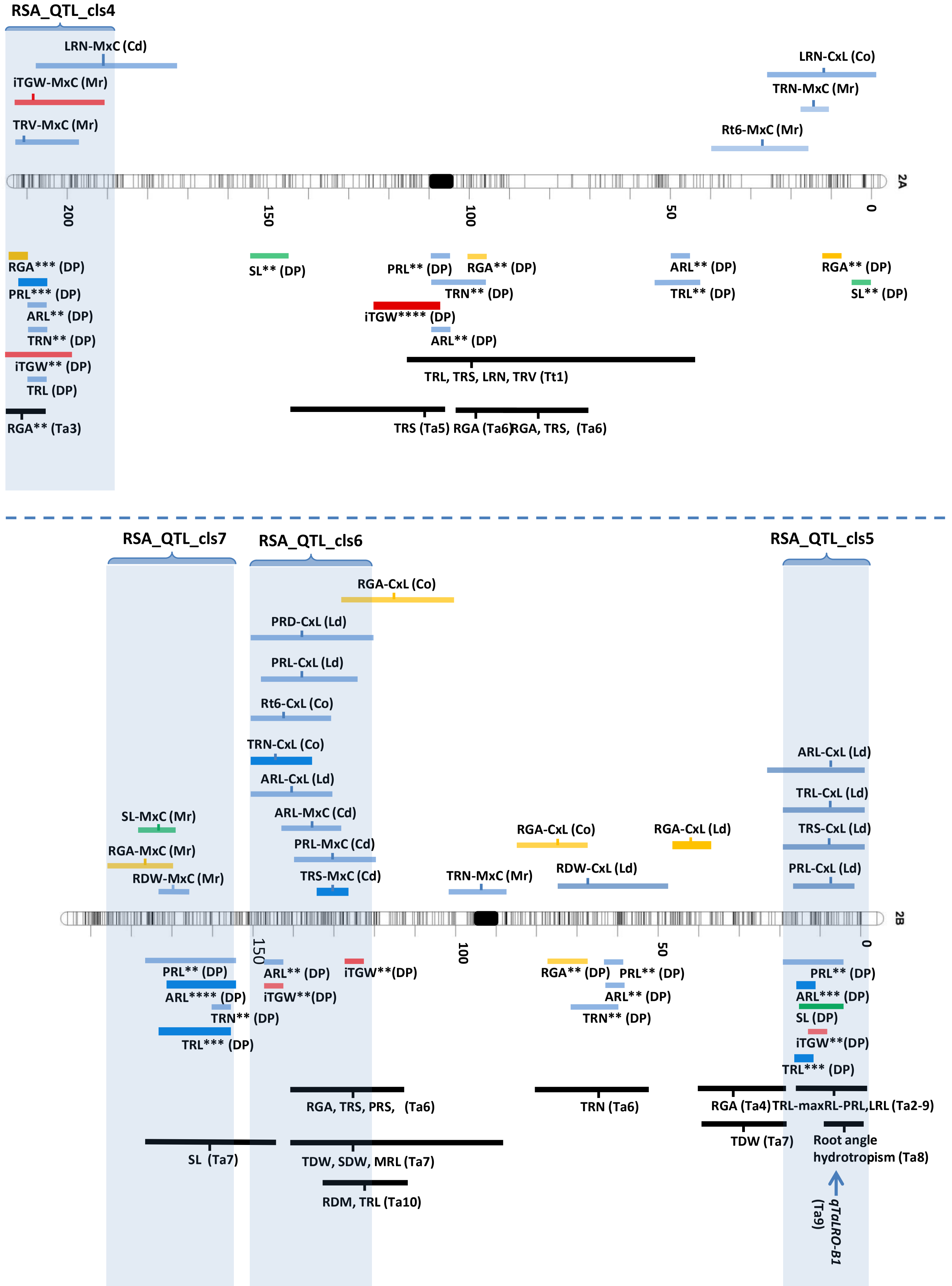

RSA_QTL_cls4
LRN-MxC (Cd)
iTGW-MxC (Mr)
TRV-MxC (Mr)
LRN-CxL (Co)
TRN-MxC (Mr)
Rt6-MxC (Mr)
2A
200
150
100
50
0
RGA*** (DP)
PRL*** (DP)
ARL** (DP)
TRN** (DP)
iTGW** (DP)
TRL (DP)
RGA** (Ta3)
SL** (DP)
PRL** (DP)
RGA** (DP)
TRN** (DP)
iTGW**** (DP)
ARL** (DP)
TRL, TRS, LRN, TRV (Tt1)
TRS (Ta5) RGA (Ta6)RGA, TRS, (Ta6)
ARL** (DP)
TRL** (DP)
RGA** (DP)
SL** (DP)
RSA_QTL_cls7
RSA_QTL_cls6
RSA_QTL_cls5
RGA-CxL (Co)
PRD-CxL (Ld)
PRL-CxL (Ld)
Rt6-CxL (Co)
TRN-CxL (Co)
ARL-CxL (Ld)
ARL-MxC (Cd)
PRL-MxC (Cd)
TRS-MxC (Cd)
SL-MxC (Mr)
RGA-MxC (Mr)
RDW-MxC (Mr)
RGA-CxL (Co)
RGA-CxL (Ld)
TRN-MxC (Mr)
RDW-CxL (Ld)
ARL-CxL (Ld)
TRL-CxL (Ld)
TRS-CxL (Ld)
PRL-CxL (Ld)
2B
150
100
50
0
PRL** (DP)
ARL**** (DP)
TRN** (DP)
TRL*** (DP)
ARL** (DP)
iTGW**(DP)
iTGW**(DP)
RGA** (DP)
PRL** (DP)
ARL** (DP)
TRN** (DP)
PRL** (DP)
ARL*** (DP)
SL (DP)
iTGW**(DP)
TRL*** (DP)
RGA, TRS, PRS, (Ta6)
TRN (Ta6)
RGA (Ta4)
TRL-maxRL-PRL,LRL (Ta2-9)
TDW (Ta7)
Root angle hydrotropism (Ta8)
SL (Ta7)
TDW, SDW, MRL (Ta7)
RDM, TRL (Ta10)
qTaLRO-B1 (Ta9)

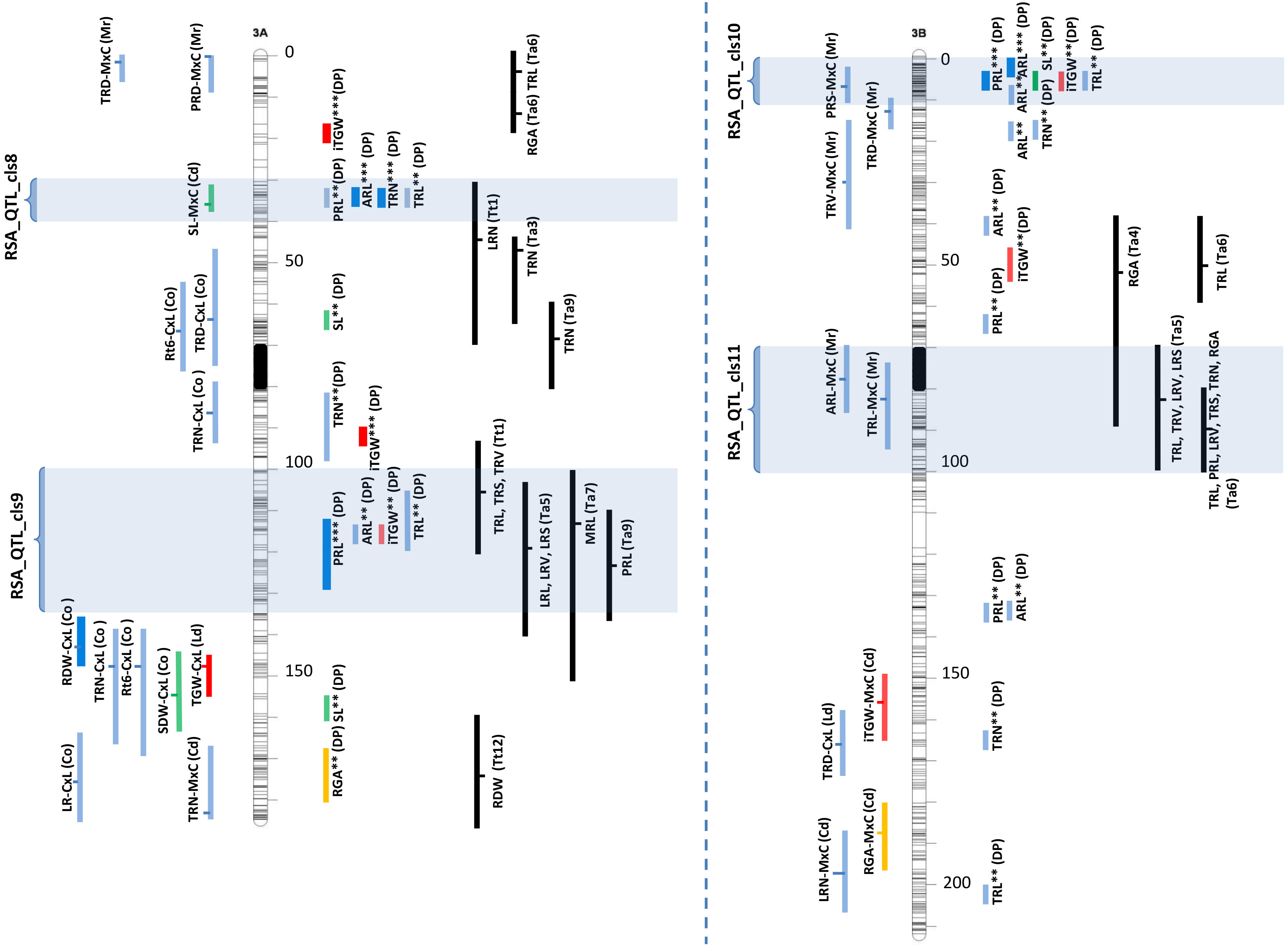

3A
3B
RSA_QTL_cls8
RSA_QTL_cls9
RSA_QTL_cls10
RSA_QTL_cls11
0
50
100
150
200
TRD-MxC (Mr)
PRD-MxC (Mr)
SL-MxC (Cd)
Rt6-CxL (Co)
TRD-CxL (Co)
TRN-CxL (Co)
RDW-CxL (Co)
TRN-CxL (Co)
Rt6-CxL (Co)
SDW-CxL (Co)
TGW-CxL (Ld)
LR-CxL (Co)
TRN-MxC (Cd)
iTGW***(DP)
PRL**(DP)
ARL*** (DP)
TRN*** (DP)
TRL** (DP)
SL** (DP)
TRN**(DP)
iTGW*** (DP)
PRL*** (DP)
ARL** (DP)
iTGW** (DP)
TRL** (DP)
SL** (DP)
RGA** (DP)
LRN (Tt1)
TRN (Ta3)
TRN (Ta9)
TRL (Ta6)
RGA (Ta6)
TRL, TRS, TRV (Tt1)
LRL, LRV, LRS (Ta5)
MRL (Ta7)
PRL (Ta9)
RDW (Tt12)
PRS-MxC (Mr)
TRD-MxC (Mr)
TRV-MxC (Mr)
ARL-MxC (Mr)
TRL-MxC (Mr)
TRD-CxL (Ld)
iTGW-MxC (Cd)
LRN-MxC (Cd)
RGA-MxC (Cd)
PRL*** (DP)
ARL*** (DP)
ARL** (DP)
SL** (DP)
iTGW** (DP)
TRL** (DP)
ARL**
TRN** (DP)
ARL** (DP)
iTGW** (DP)
PRL** (DP)
RGA (Ta4)
TRL (Ta6)
TRL, TRV, LRV, LRS (Ta5)
TRL, PRL, LRV, TRS, TRN, RGA (Ta6)
PRL** (DP)
ARL** (DP)
TRN** (DP)
TRL** (DP)

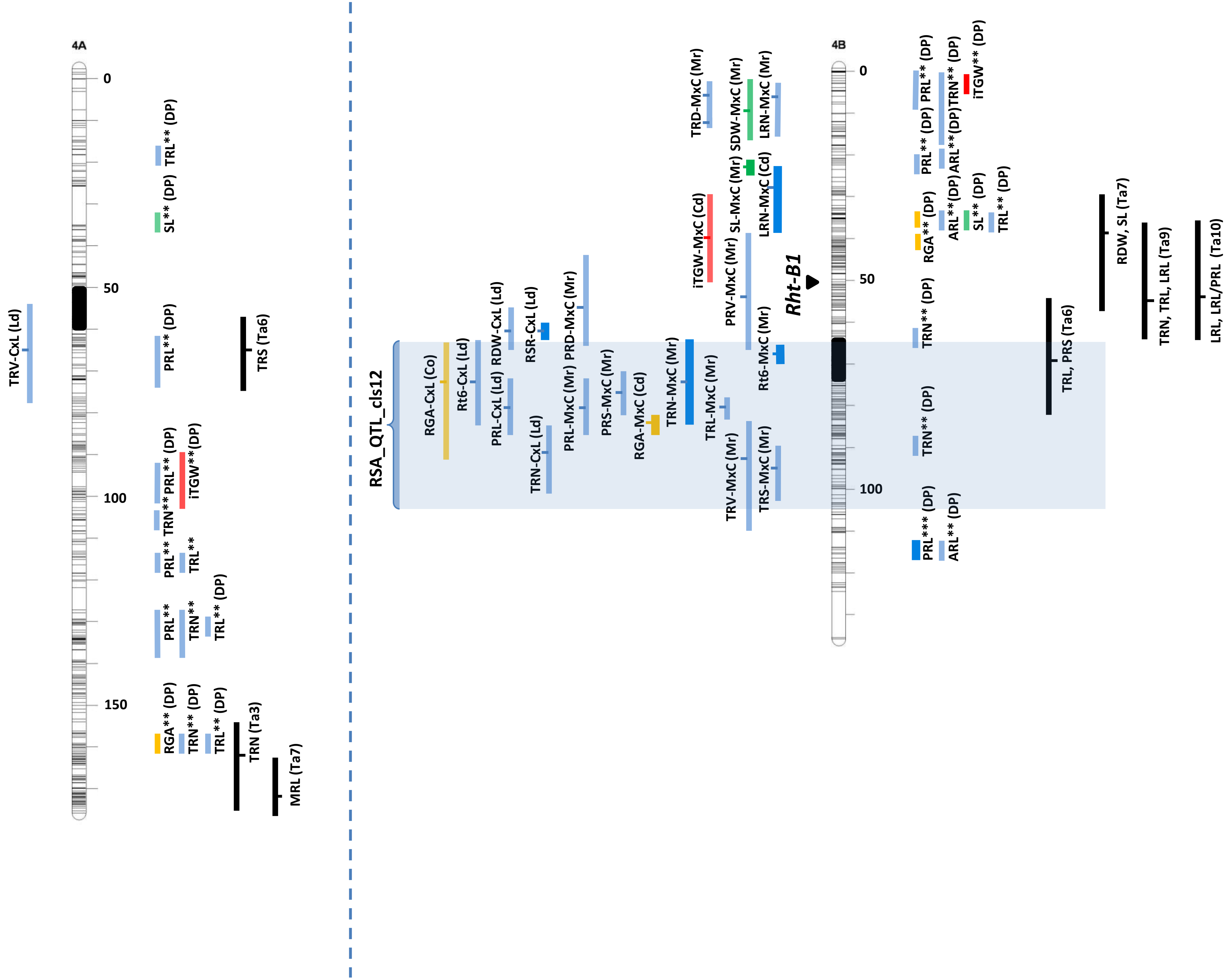
4A
0
50
100
150
TRV-CxL (Ld)
TRL** (DP)
SL** (DP)
PRL** (DP)
TRS (Ta6)
PRL** (DP)
iTGW**(DP)
TRN**
PRL**
TRL**
PRL**
TRN**
TRL** (DP)
RGA** (DP)
TRN** (DP)
TRL** (DP)
TRN (Ta3)
MRL (Ta7)
RSA_QTL_cls12
RGA-CxL (Co)
Rt6-CxL (Ld)
RDW-CxL (Ld)
PRL-CxL (Ld)
RSR-CxL (Ld)
TRN-CxL (Ld)
PRD-MxC (Mr)
PRL-MxC (Mr)
PRS-MxC (Mr)
RGA-MxC (Cd)
TRN-MxC (Mr)
TRD-MxC (Mr)
iTGW-MxC (Cd)
TRL-MxC (Mr)
SDW-MxC (Mr)
SL-MxC (Mr)
PRV-MxC (Mr)
TRV-MxC (Mr)
LRN-MxC (Mr)
LRN-MxC (Cd)
Rt6-MxC (Mr)
TRS-MxC (Mr)
Rht-B1
4B
0
50
100
PRL** (DP)
PRL** (DP)
TRN** (DP)
ARL**(DP)
iTGW** (DP)
RGA** (DP)
ARL**(DP)
SL** (DP)
TRL** (DP)
TRN** (DP)
TRN** (DP)
PRL*** (DP)
ARL** (DP)
TRL, PRS (Ta6)
RDW, SL (Ta7)
TRN, TRL, LRL (Ta9)
LRL, LRL/PRL (Ta10)

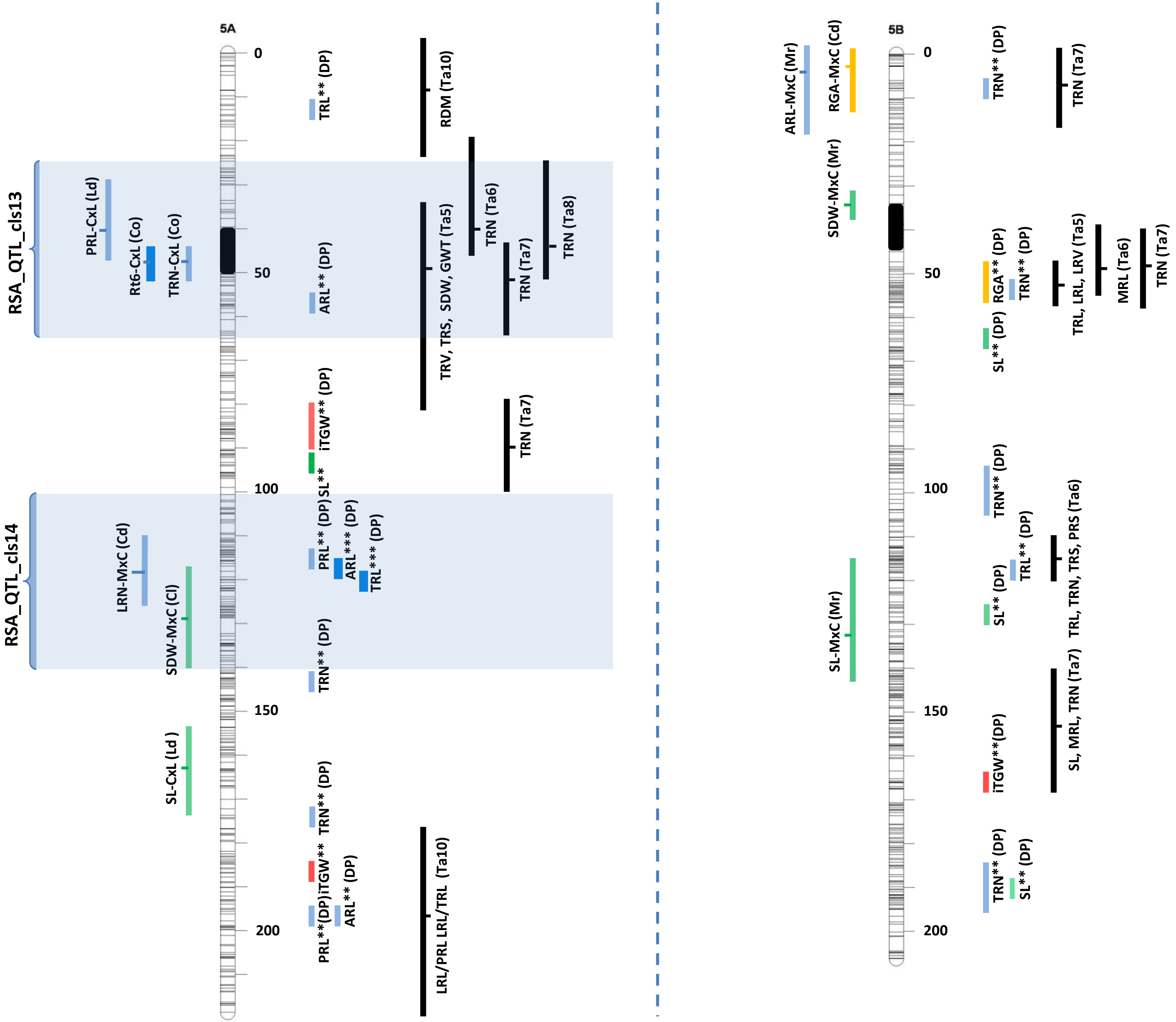

5A
0
50
100
150
200
RSA_QTL_cls13
RSA_QTL_cls14
PRL-CxL (Ld)
Rt6-CxL (Co)
TRN-CxL (Co)
LRN-MxC (Cd)
SDW-MxC (Cl)
SL-CxL (Ld)
TRL** (DP)
ARL** (DP)
iTGW** (DP)
SL**
PRL** (DP)
ARL*** (DP)
TRL*** (DP)
TRN** (DP)
TRN** (DP)
iTGW**
PRL**(DP)
ARL** (DP)
RDM (Ta10)
TRV, TRS, SDW, GWT (Ta5)
TRN (Ta6)
TRN (Ta7)
TRN (Ta8)
TRN (Ta7)
LRL/PRL LRL/TRL (Ta10)
5B
0
50
100
150
200
ARL-MxC (Mr)
RGA-MxC (Cd)
SDW-MxC (Mr)
SL-MxC (Mr)
TRN** (DP)
RGA** (DP)
TRN** (DP)
SL** (DP)
TRN** (DP)
TRL** (DP)
SL** (DP)
iTGW**(DP)
TRN** (DP)
SL** (DP)
TRN (Ta7)
TRL, LRL, LRV (Ta5)
MRL (Ta6)
TRN (Ta7)
TRL, TRN, TRS, PRS (Ta6)
SL, MRL, TRN (Ta7)

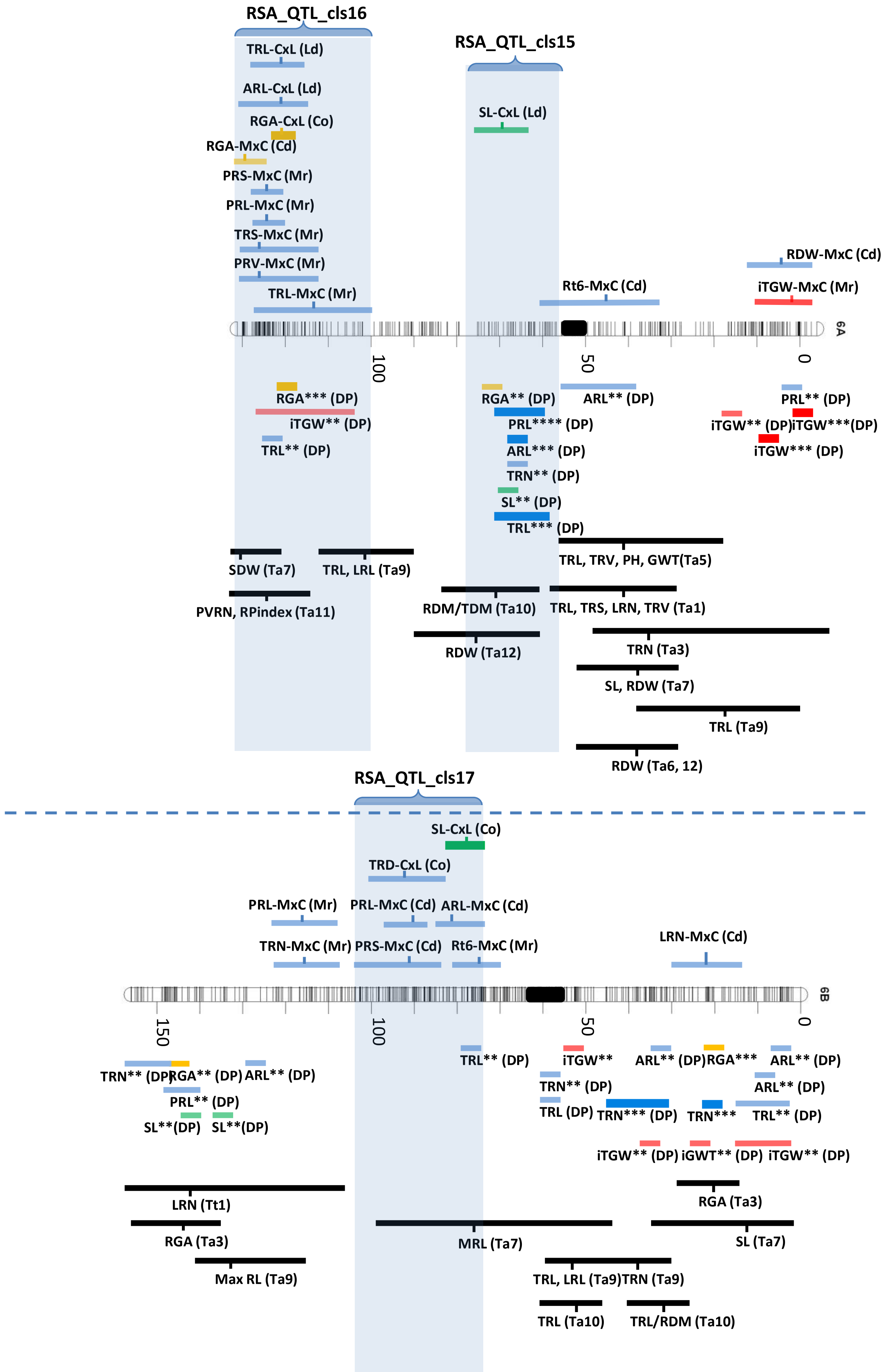
RSA_QTL_cls16
RSA_QTL_cls15
RSA_QTL_cls17
6A
6B
0
50
100
150
TRL-CxL (Ld)
ARL-CxL (Ld)
RGA-CxL (Co)
RGA-MxC (Cd)
PRS-MxC (Mr)
PRL-MxC (Mr)
TRS-MxC (Mr)
PRV-MxC (Mr)
TRL-MxC (Mr)
SL-CxL (Ld)
Rt6-MxC (Cd)
RDW-MxC (Cd)
iTGW-MxC (Mr)
RGA*** (DP)
iTGW** (DP)
TRL** (DP)
RGA** (DP)
PRL**** (DP)
ARL*** (DP)
TRN** (DP)
SL** (DP)
TRL*** (DP)
ARL** (DP)
PRL** (DP)
iTGW** (DP)iTGW***(DP)
iTGW*** (DP)
SDW (Ta7)
TRL, LRL (Ta9)
PVRN, RPindex (Ta11)
TRL, TRV, PH, GWT(Ta5)
RDM/TDM (Ta10)
TRL, TRS, LRN, TRV (Ta1)
RDW (Ta12)
TRN (Ta3)
SL, RDW (Ta7)
TRL (Ta9)
RDW (Ta6, 12)
SL-CxL (Co)
TRD-CxL (Co)
PRL-MxC (Mr)
PRL-MxC (Cd)
ARL-MxC (Cd)
TRN-MxC (Mr)
PRS-MxC (Cd)
Rt6-MxC (Mr)
LRN-MxC (Cd)
TRL** (DP)
iTGW**
ARL** (DP)RGA***
ARL** (DP)
TRN** (DPRGA** (DP) ARL** (DP)
PRL** (DP)
SL**(DP)
SL**(DP)
TRN** (DP)
TRL (DP)
TRN*** (DP)
TRN***
ARL** (DP)
TRL** (DP)
iTGW** (DP) iGWT** (DP) iTGW** (DP)
LRN (Tt1)
RGA (Ta3)
Max RL (Ta9)
MRL (Ta7)
RGA (Ta3)
SL (Ta7)
TRL, LRL (Ta9)TRN (Ta9)
TRL (Ta10)
TRL/RDM (Ta10)

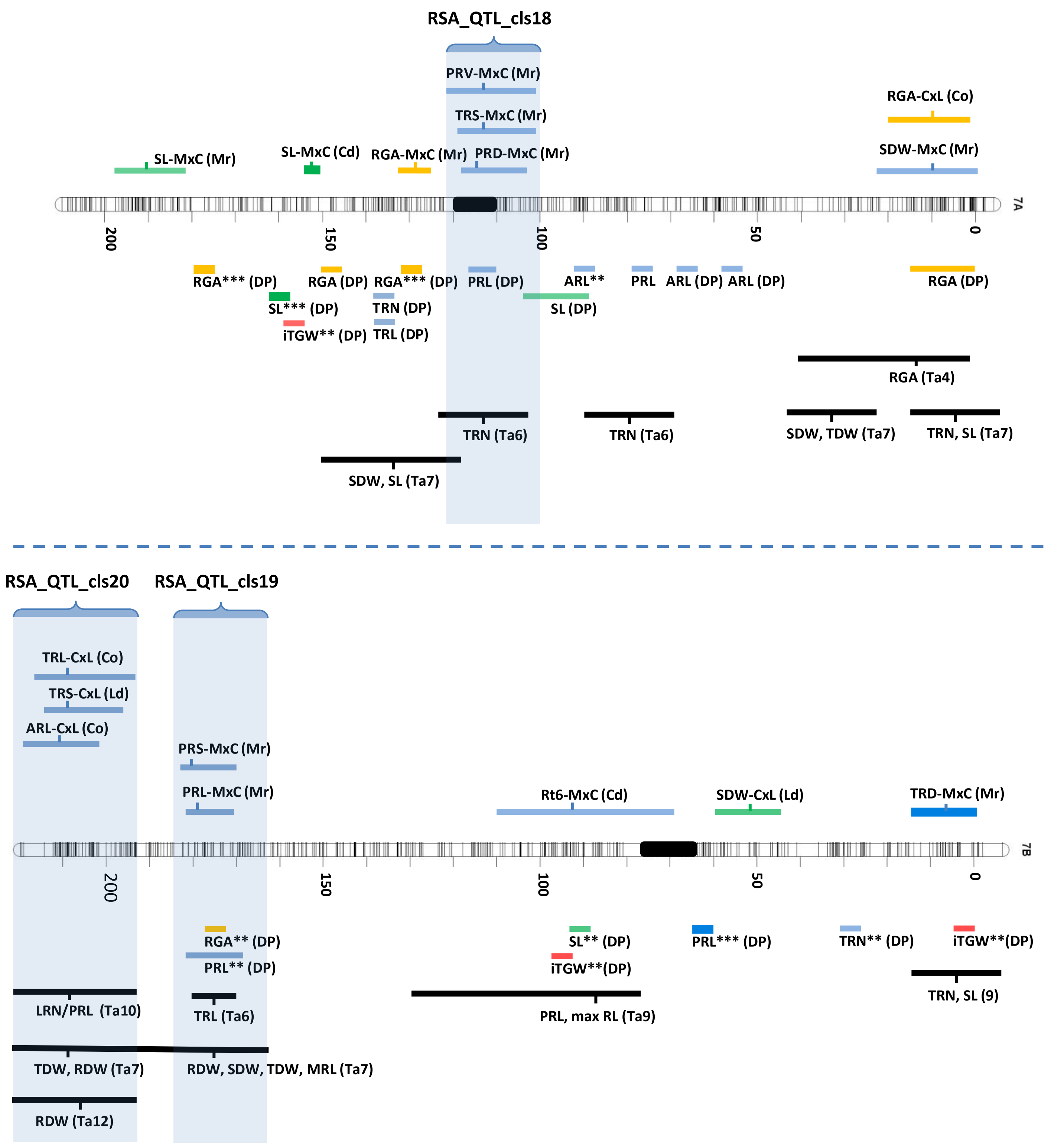
RSA_QTL_cls18
PRV-MxC (Mr)
TRS-MxC (Mr)
RGA-CxL (Co)
SL-MxC (Mr)
SL-MxC (Cd)
RGA-MxC (Mr)
PRD-MxC (Mr)
SDW-MxC (Mr)
7A
200
150
100
50
0
RGA*** (DP)
RGA (DP)
RGA*** (DP)
PRL (DP)
ARL**
PRL
ARL (DP)
ARL (DP)
RGA (DP)
SL*** (DP)
TRN (DP)
SL (DP)
iTGW** (DP)
TRL (DP)
RGA (Ta4)
TRN (Ta6)
TRN (Ta6)
SDW, TDW (Ta7)
TRN, SL (Ta7)
SDW, SL (Ta7)
RSA_QTL_cls20
RSA_QTL_cls19
TRL-CxL (Co)
TRS-CxL (Ld)
ARL-CxL (Co)
PRS-MxC (Mr)
PRL-MxC (Mr)
Rt6-MxC (Cd)
SDW-CxL (Ld)
TRD-MxC (Mr)
7B
200
150
100
50
0
RGA** (DP)
SL** (DP)
PRL*** (DP)
TRN** (DP)
iTGW**(DP)
PRL** (DP)
iTGW**(DP)
TRN, SL (9)
LRN/PRL (Ta10)
TRL (Ta6)
PRL, max RL (Ta9)
TDW, RDW (Ta7)
RDW, SDW, TDW, MRL (Ta7)
RDW (Ta12)

Supplementary Figure S3. Schematic representation of root system architecture (RSA) QTLs , grain yield (GY) and thousand grain weight (TKW) in tetraploid wheat projected onto an SNP-based tetraploid consensus map.

Root system architecture (RSA) QTLs were identified in two durum wheat mapping population and the association mapping panel and RSA QTLs from previously published studies in wheat are projected. The tetraploid wheat consensus map used as a reference has been published by Maccaferri et al. (2015).

QTLs from Colosseo × Lloyd and Meridiano × Claudio are reported on the left of chromosome bars while association mapping QTLs (AM-QTLs) are reported on the right. QTLs for root length and number are reported as blue-filled bars, seminal root angle (RGA) QTLs are reported in orange, shoot-related QTLs in green, grain weigth (GWT) QTLs in red.

QTLs from RIL populations with R^2 < 10% are reported as thin half-tone coloured bars, while QTLs with R^2 > 10% are reported as tick full-tone bars.
The parental allele with increasing trait effect is reported as round-bracketed using acronyms: **Co**, Colosseo; **Ld**, Lloyd; **Me**, Meridiano; **Cl**, Claudio.

RSA QTLs from association mapping (Durum Panel, DP) are reported on the left of chromosome bars together with their significance level: **, marker-wise significance P 0.01; ***, marker-wise significance, P 0.001; ****, experiment-wise significance, P 0.05

Grain yield (GY) and grain weight (TKW) QTLs from association mapping (Durum Panel, DP) are reported on the right of the chromosome bars. GY and TKW QTLs have been searched in the Durum Panel using data from 15 field trials carried out in Mediterranean environments as reported in Maccaferri et al. (2011). Association mapping QTLs have been graphically reported as a plot of QTL density on a 1 cM basis along the chromosomes, based on the QTL significance intervals. According to Maccaferri et al. (2011), QTL density has been reported as a cumulated density plot for: (i) highly stressed low-yielding environments (**red plot**, three environments), (ii) medium yielding, stressed environments (**green plot**, five envs.), (iii) high-yielding environments (**dark violet plot**, seven envs.).

Major RSA QTL-clusters based on concomitant co-location of RSA QTL confidence intervals from multiple wheat mapping stocks are reported as grey-banded intervals.

RSA and field trait acronyms: TRN, Total root number; R6th, Presence of the 6th asymmetric seminal root; PRL, primary root length; TRL, total root length; ARL, average root length; LRN, lateral root number; RGA, seminal root angle; RDW, root dry weight; SDW, shoot dry weight; SL, shoot length; RSR, root-to-shoot ratio; RS, root surface; RD, root diameter; RV, root volume; GY, grain yield; iGWT, grain weight of seeds used in the RSA experiment; fGWT, grain weight in field trials

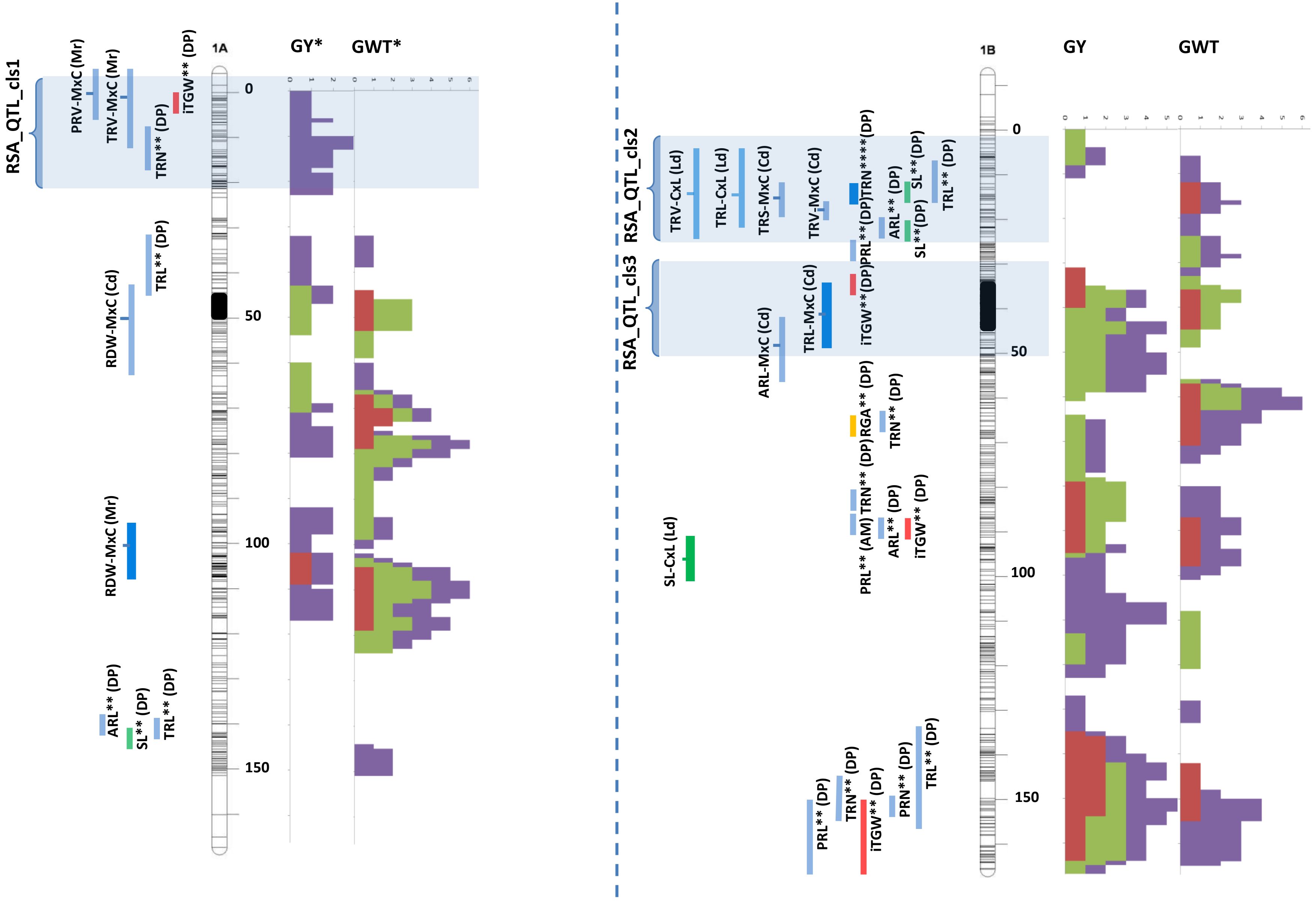

*: QTL density plot (QTL number over 15 environments in total, plotted on a 1cM basis)

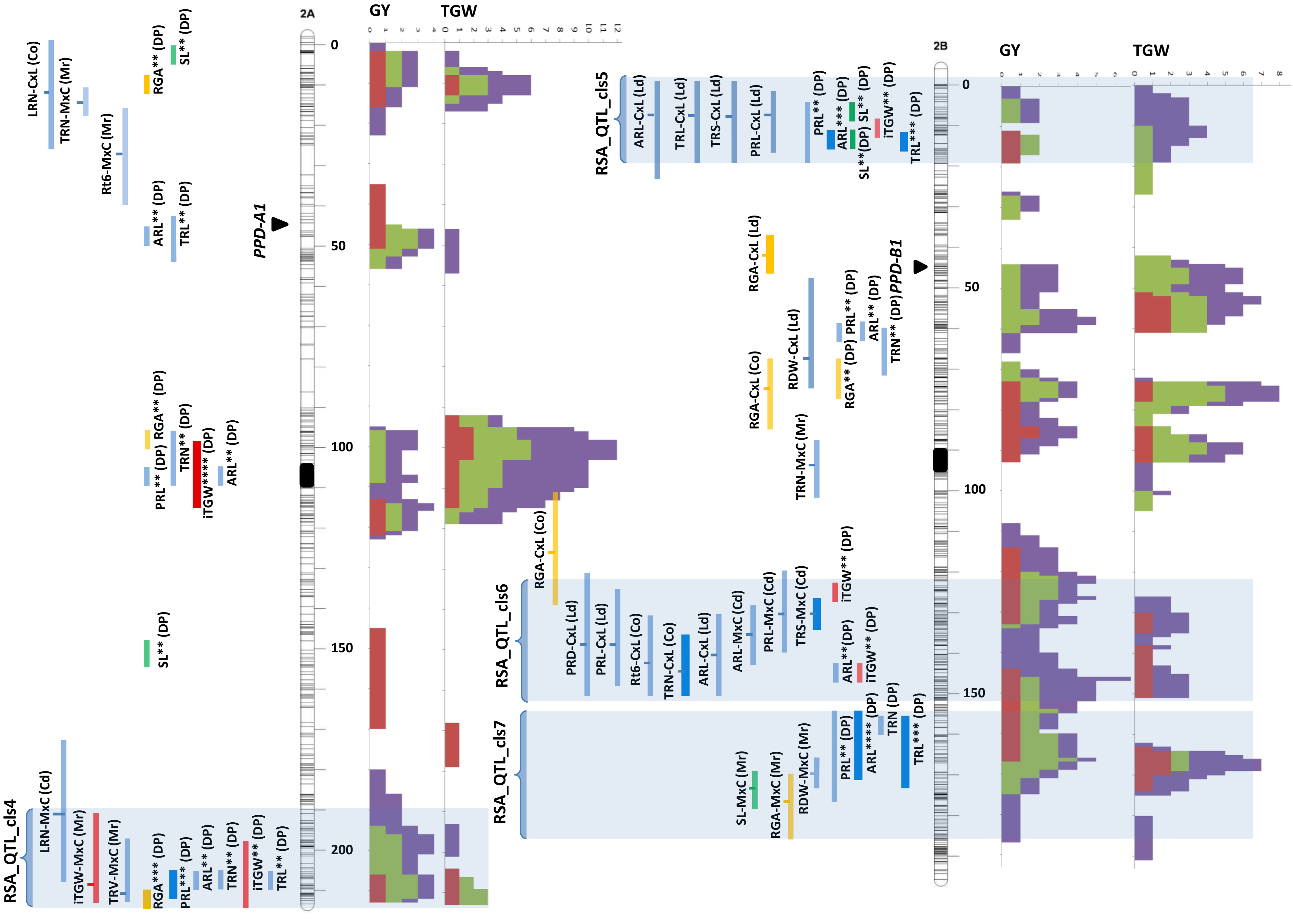
2A
GY
TGW
PPD-A1
RSA_QTL_cls4
LRN-CxL (Co)
TRN-MxC (Mr)
Rt6-MxC (Mr)
RGA** (DP)
SL** (DP)
ARL** (DP)
TRL** (DP)
PRL** (DP)
RGA** (DP)
TRN** (DP)
iTGW**** (DP)
ARL** (DP)
SL** (DP)
LRN-MxC (Cd)
iTGW-MxC (Mr)
TRV-MxC (Mr)
RGA*** (DP)
PRL*** (DP)
ARL** (DP)
TRN** (DP)
iTGW** (DP)
TRL** (DP)
0
50
100
150
200
2B
PPD-B1
RSA_QTL_cls5
ARL-CxL (Ld)
TRL-CxL (Ld)
TRS-CxL (Ld)
PRL-CxL (Ld)
PRL** (DP)
ARL*** (DP)
SL**(DP)
SL** (DP)
iTGW** (DP)
TRL*** (DP)
RGA-CxL (Ld)
RGA-CxL (Co)
RDW-CxL (Ld)
RGA** (DP)
PRL** (DP)
ARL** (DP)
TRN** (DP)
TRN-MxC (Mr)
RSA_QTL_cls6
RGA-CxL (Co)
PRD-CxL (Ld)
PRL-CxL (Ld)
Rt6-CxL (Co)
TRN-CxL (Co)
ARL-CxL (Ld)
ARL-MxC (Cd)
PRL-MxC (Cd)
TRS-MxC (Cd)
iTGW** (DP)
ARL**(DP)
iTGW** (DP)
RSA_QTL_cls7
SL-MxC (Mr)
RGA-MxC (Mr)
RDW-MxC (Mr)
PRL** (DP)
ARL**** (DP)
TRN (DP)
TRL*** (DP)

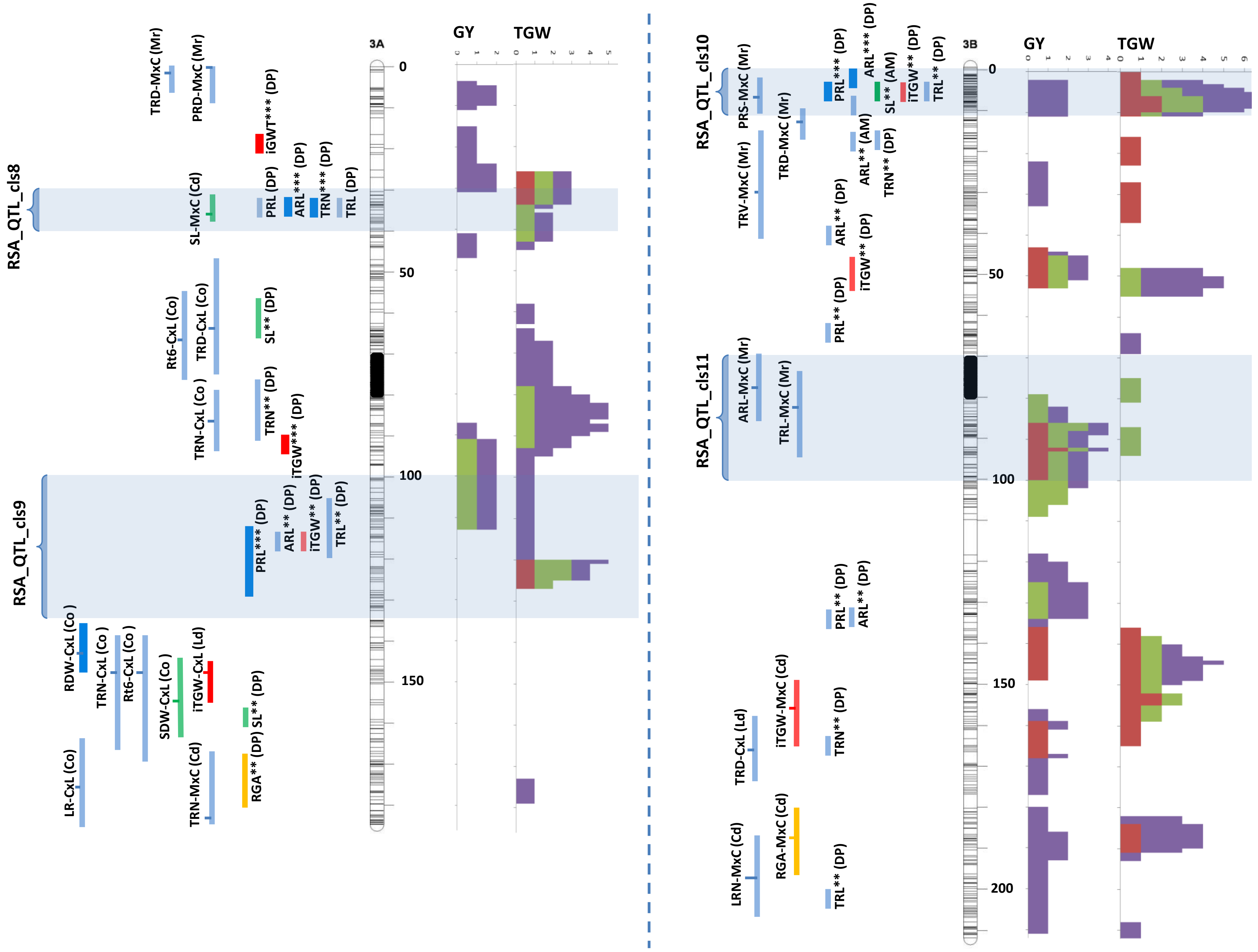
3A
GY
TGW
3B
RSA_QTL_cls8
RSA_QTL_cls9
RSA_QTL_cls10
RSA_QTL_cls11
TRD-MxC (Mr)
PRD-MxC (Mr)
iGWT*** (DP)
SL-MxC (Cd)
PRL (DP)
ARL*** (DP)
TRN*** (DP)
TRL (DP)
Rt6-CxL (Co)
TRD-CxL (Co)
SL** (DP)
TRN-CxL (Co)
TRN** (DP)
iTGW*** (DP)
PRL*** (DP)
ARL** (DP)
iTGW** (DP)
TRL** (DP)
RDW-CxL (Co)
TRN-CxL (Co)
Rt6-CxL (Co)
SDW-CxL (Co)
iTGW-CxL (Ld)
SL** (DP)
LR-CxL (Co)
TRN-MxC (Cd)
RGA** (DP)
PRS-MxC (Mr)
TRD-MxC (Mr)
TRV-MxC (Mr)
PRL*** (DP)
ARL*** (DP)
SL** (AM)
iTGW** (DP)
TRL** (DP)
ARL** (AM)
TRN** (DP)
ARL** (DP)
iTGW** (DP)
PRL** (DP)
ARL-MxC (Mr)
TRL-MxC (Mr)
PRL** (DP)
ARL** (DP)
TRD-CxL (Ld)
iTGW-MxC (Cd)
TRN** (DP)
LRN-MxC (Cd)
RGA-MxC (Cd)
TRL** (DP)
0
50
100
150
200

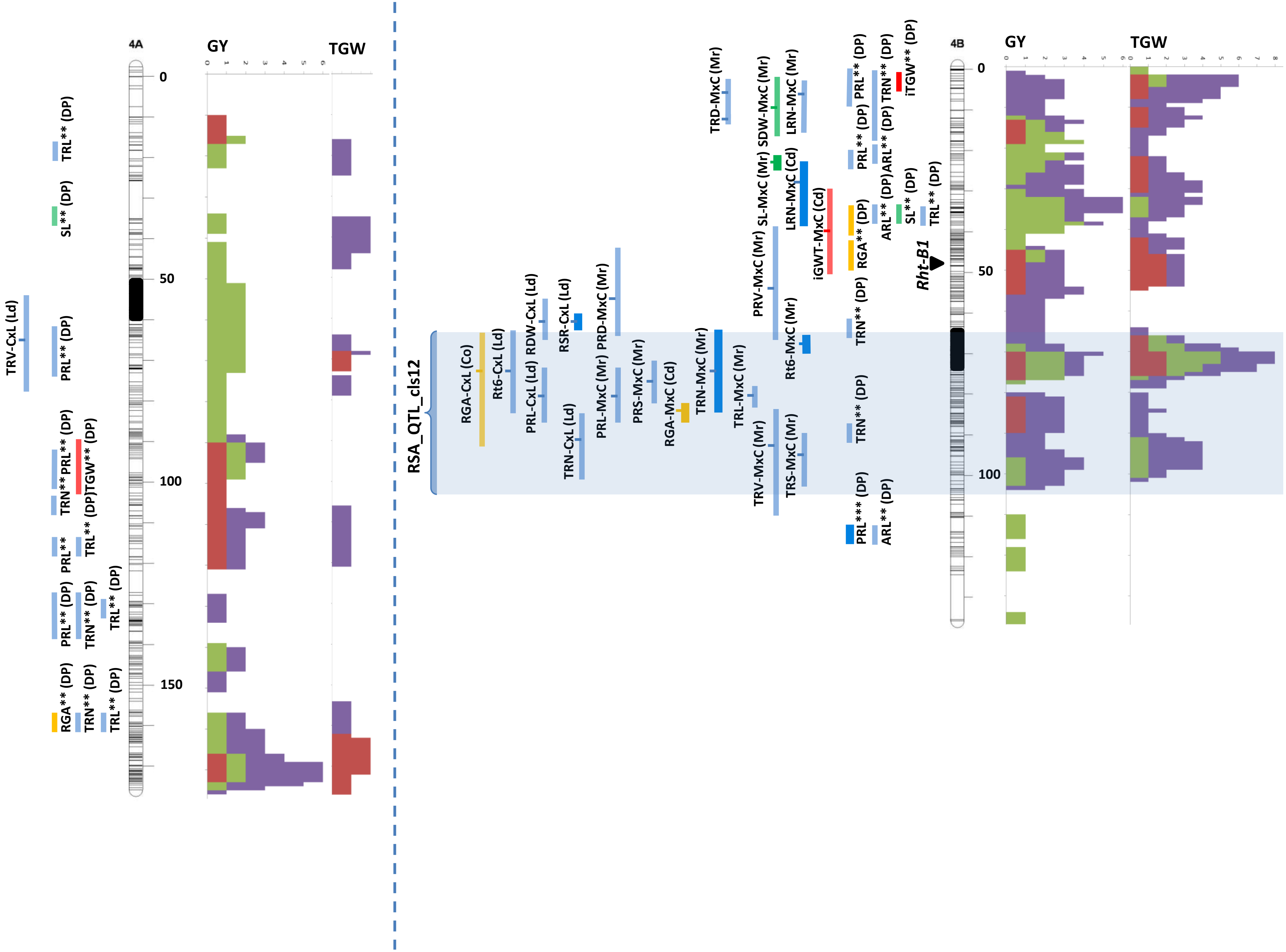

4A
GY
TGW
0
50
100
150
TRL** (DP)
SL** (DP)
TRV-CxL (Ld)
PRL** (DP)
TRN** PRL** (DP)
TGW** (DP)
TRL** (DP)
PRL**
PRL** (DP)
TRN** (DP)
TRL** (DP)
RGA** (DP)
TRN** (DP)
TRL** (DP)
RSA_QTL_cls12
RGA-CxL (Co)
Rt6-CxL (Ld)
PRL-CxL (Ld)
RDW-CxL (Ld)
TRN-CxL (Ld)
RSR-CxL (Ld)
PRD-MxC (Mr)
PRL-MxC (Mr)
PRS-MxC (Mr)
RGA-MxC (Cd)
TRN-MxC (Mr)
TRL-MxC (Mr)
TRD-MxC (Mr)
SDW-MxC (Mr)
SL-MxC (Mr)
PRV-MxC (Mr)
TRV-MxC (Mr)
LRN-MxC (Mr)
LRN-MxC (Cd)
Rt6-MxC (Mr)
TRS-MxC (Mr)
iGWT-MxC (Cd)
PRL** (DP)
PRL** (DP)
RGA** (DP)
TRN** (DP)
TRN** (DP)
PRL*** (DP)
TRN** (DP)
ARL** (DP)
ARL** (DP)
ARL** (DP)
iTGW** (DP)
SL** (DP)
TRL** (DP)
Rht-B1
4B
GY
TGW
0
50
100

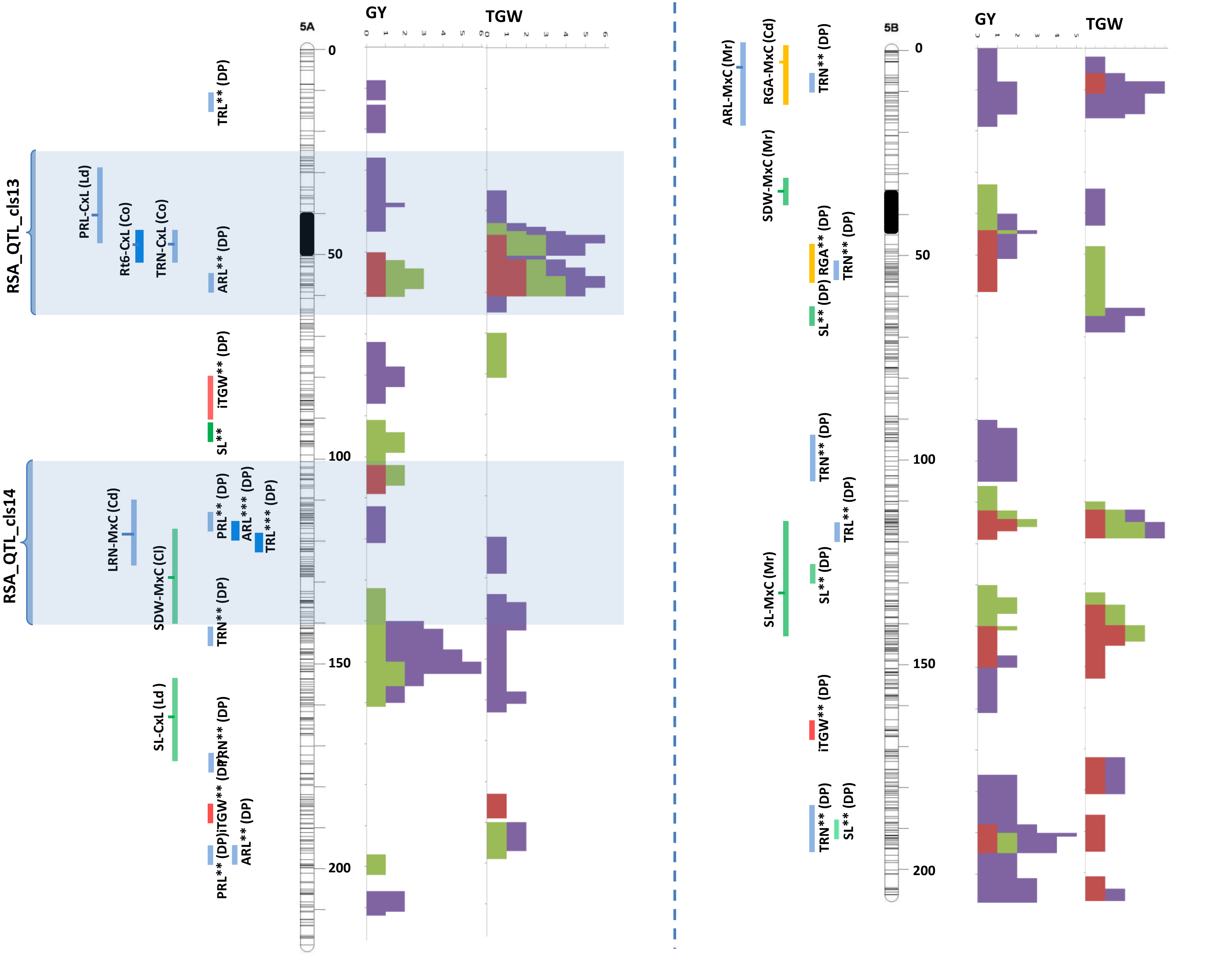
5A
GY
TGW
0
50
100
150
200
RSA_QTL_cls13
RSA_QTL_cls14
TRL** (DP)
PRL-CxL (Ld)
Rt6-CxL (Co)
TRN-CxL (Co)
ARL** (DP)
iTGW** (DP)
SL**
LRN-MxC (Cd)
SDW-MxC (Cl)
PRL** (DP)
ARL*** (DP)
TRL*** (DP)
TRN** (DP)
SL-CxL (Ld)
TRN** (DP)
iTGW** (DP)
PRL** (DP)
ARL** (DP)
5B
GY
TGW
0
50
100
150
200
ARL-MxC (Mr)
RGA-MxC (Cd)
TRN** (DP)
SDW-MxC (Mr)
RGA** (DP)
TRN** (DP)
SL** (DP)
TRN** (DP)
TRL** (DP)
SL-MxC (Mr)
SL** (DP)
iTGW** (DP)
TRN** (DP)
SL** (DP)

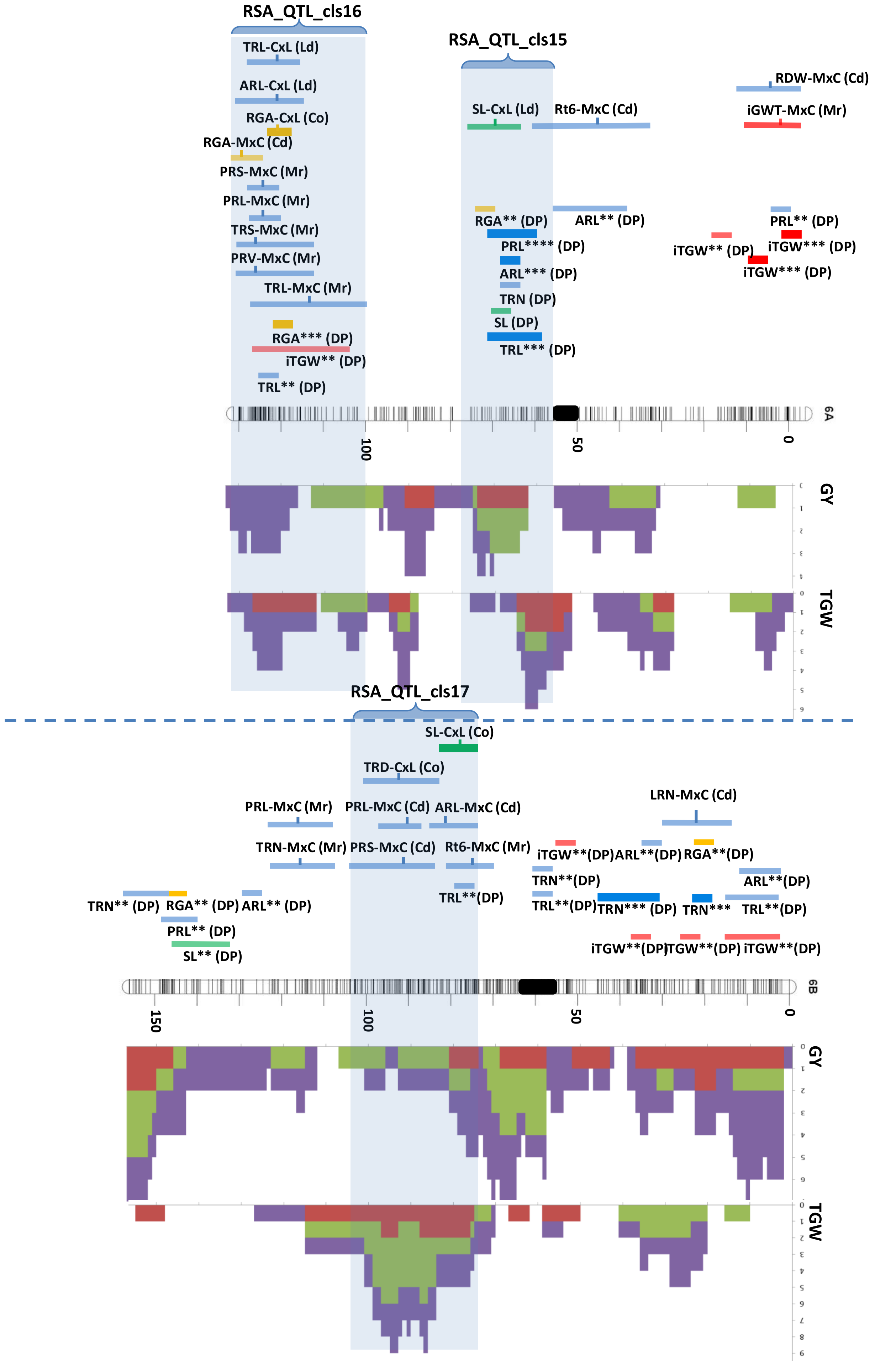

RSA_QTL_cls16
TRL-CxL (Ld)
ARL-CxL (Ld)
RGA-CxL (Co)
RGA-MxC (Cd)
PRS-MxC (Mr)
PRL-MxC (Mr)
TRS-MxC (Mr)
PRV-MxC (Mr)
TRL-MxC (Mr)
RGA*** (DP)
iTGW** (DP)
TRL** (DP)
RSA_QTL_cls15
SL-CxL (Ld)
Rt6-MxC (Cd)
RGA** (DP)
ARL** (DP)
PRL**** (DP)
ARL*** (DP)
TRN (DP)
SL (DP)
TRL*** (DP)
RDW-MxC (Cd)
iGWT-MxC (Mr)
PRL** (DP)
iTGW** (DP)
iTGW*** (DP)
iTGW*** (DP)
6A
0
50
100
GY
TGW
RSA_QTL_cls17
SL-CxL (Co)
TRD-CxL (Co)
PRL-MxC (Mr)
PRL-MxC (Cd)
ARL-MxC (Cd)
LRN-MxC (Cd)
TRN-MxC (Mr)
PRS-MxC (Cd)
Rt6-MxC (Mr)
iTGW**(DP)
ARL**(DP)
RGA**(DP)
TRN**(DP)
ARL**(DP)
TRN** (DP)
RGA** (DP)
ARL** (DP)
TRL**(DP)
TRL**(DP)
TRN*** (DP)
TRN***
TRL**(DP)
PRL** (DP)
SL** (DP)
iTGW**(DP)
iTGW**(DP)
iTGW**(DP)
6B
0
50
100
150
GY
TGW

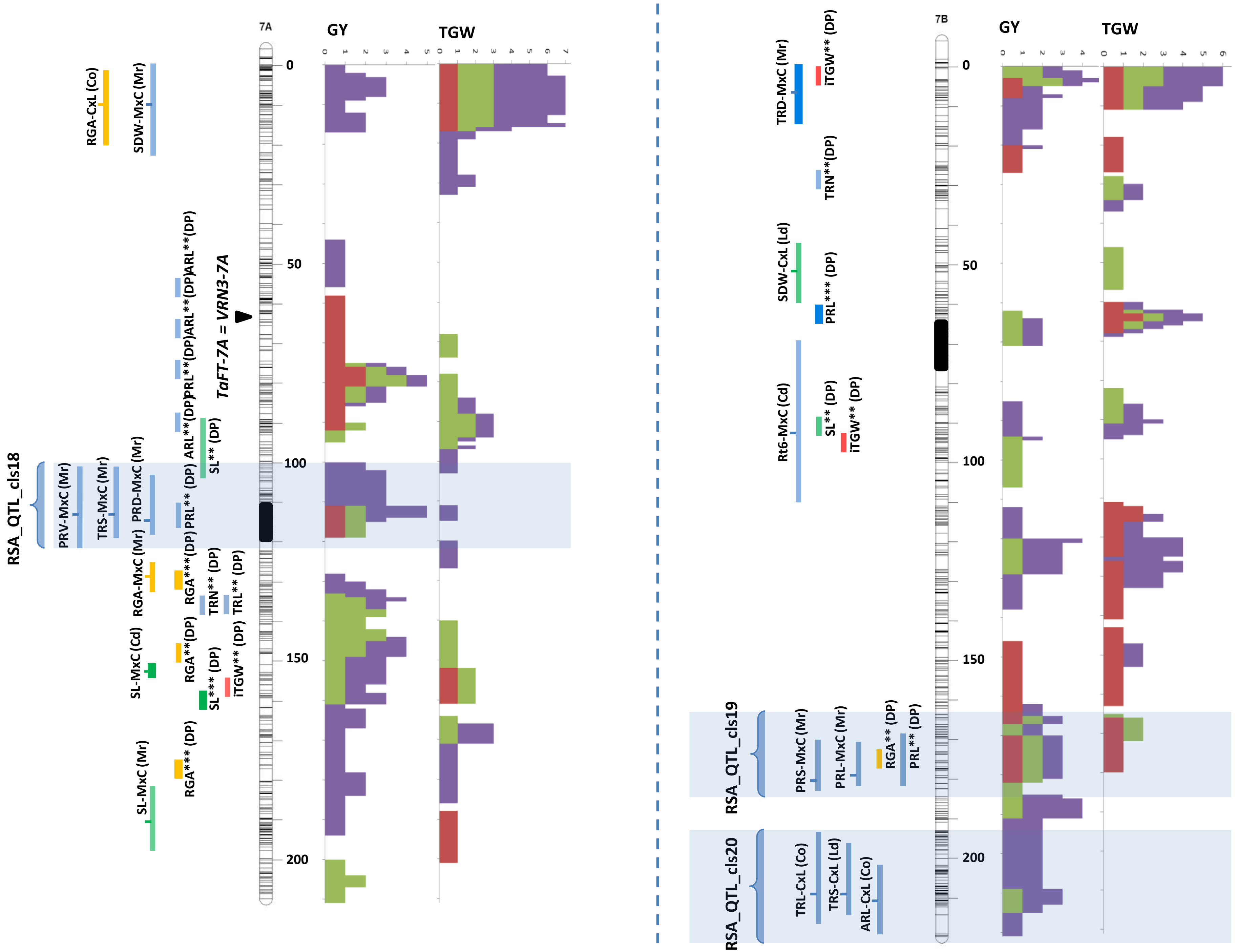

7A
GY
TGW
RGA-CxL (Co)
SDW-MxC (Mr)
TaFT-7A = VRN3-7A
ARL**(DP)
PRL**(DP)
ARL**(DP)
SL** (DP)
PRL** (DP)
RSA_QTL_cls18
PRV-MxC (Mr)
TRS-MxC (Mr)
PRD-MxC (Mr)
RGA-MxC (Mr)
RGA*** (DP)
TRN** (DP)
TRL** (DP)
SL-MxC (Cd)
RGA**(DP)
SL*** (DP)
iTGW** (DP)
SL-MxC (Mr)
RGA*** (DP)
7B
TRD-MxC (Mr)
iTGW** (DP)
TRN**(DP)
SDW-CxL (Ld)
PRL*** (DP)
Rt6-MxC (Cd)
SL** (DP)
iTGW** (DP)
RSA_QTL_cls19
PRS-MxC (Mr)
PRL-MxC (Mr)
RGA** (DP)
PRL** (DP)
RSA_QTL_cls20
TRL-CxL (Co)
TRS-CxL (Ld)
ARL-CxL (Co)

Supplementary Figure S4. Schematic ideotypes of wheat plants with shallow and deep root system architecture consequent to different seminal root growth angle (RGA) QTLs which can potentially contribute towards these ideotypes through marker-assisted selection.

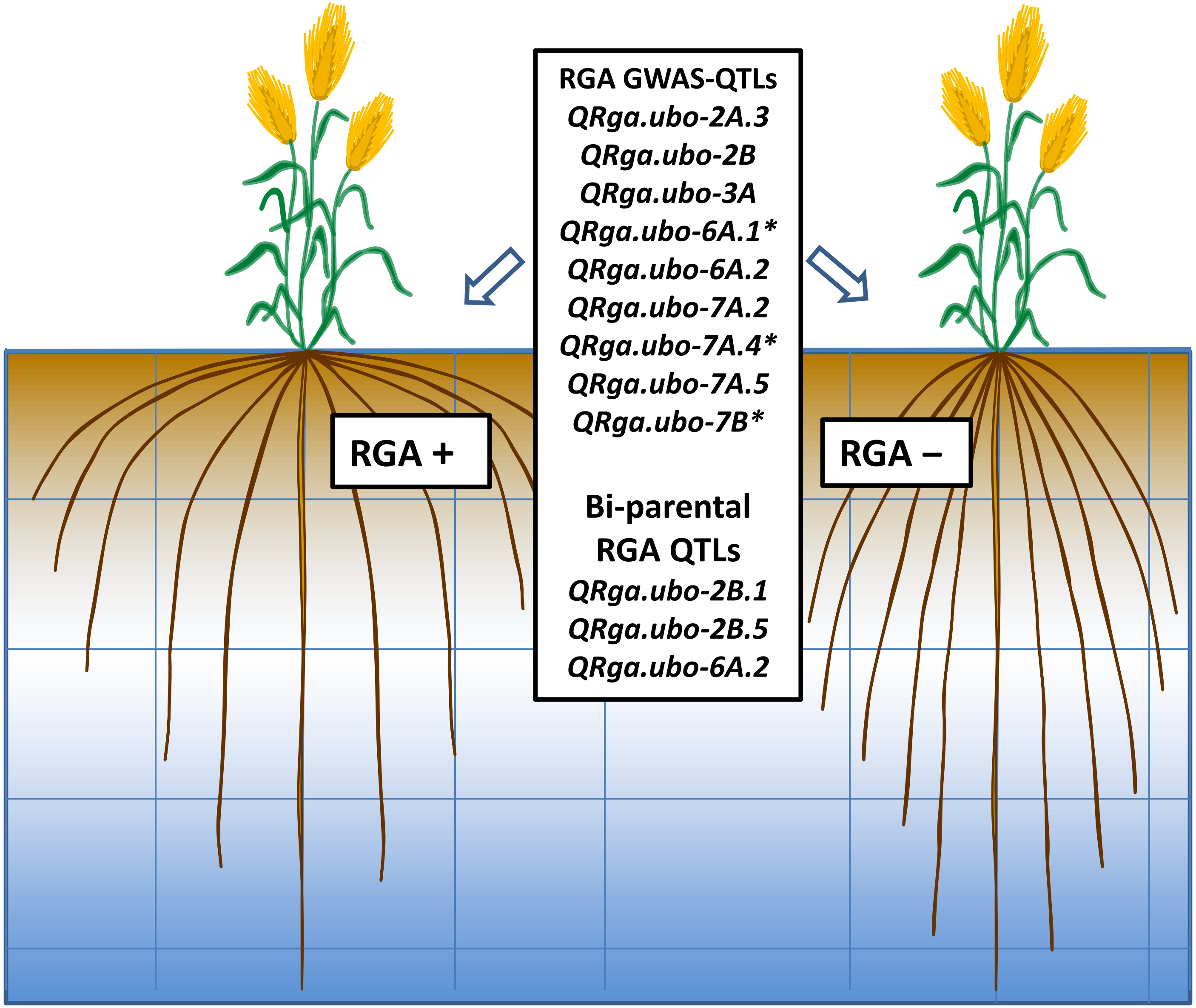

* QTL effect consistently associated to GY across the majority of 15 Mediterranean environments (see Suppl. Table 7)
