# Supplementary material for: Prioritizing quantitative trait loci for root system architecture in tetraploid wheat
Source: J Exp Bot. 2016 Feb 13;67(4):1161–78. doi: 10.1093/jxb/erw039 (PMC4753857; doi:10.1093/jxb/erw039)
Supplement: Supplementary Data [file supp_erw039_supplementary_methods_Tables_S1_S3.docx]

**Title: Prioritizing QTLs for root system architecture in tetraploid wheat**

**Supplementary Text S1-S3 & Supplementary Table S1-S3**

Authors: Marco Maccaferri*,^1^, Walid El-Feki^1^,^2^, Nazemi Gashemali^1^,^3^, Silvio Salvi^1^, Maria Angela Canè M^1^, Chiara Colalongo^1^, Sandra Stefanelli^1^, Roberto Tuberosa^1^

^1^Department of Agricultural Sciences, University of Bologna, 40127 Bologna, Italy

^2^Department of Crop Sciences, Faculty of Agriculture, Alexandria University, 23714 Alexandria, Egypt

^3^Department of Agriculture, Hajiabad Branch, Islamic Azad University, 21100 Hajiabad, Iran

*To whom correspondence should be addressed: marco.maccaferri@unibo.it

**Supplementary Text S1. QTL analysis in the two recombinant inbred line populations. Methodology.**

QTL analysis was carried out based on single-marker analysis and multiple interval mapping (MIM, Kao et al., 1999) in Windows QTL Cartographer v2.5 (http://statgen.ncsu.edu/qtlcart/WQTLCart.htm).

For MIM, the initial model was found using the MIM forward search method and the Bayesian Information Content criterion (Zeng *et al*., 1999) and a walk speed of 1 cM-step. The QTL model was iteratively refined by repeating cycles of QTL search, significance test and refinement of QTL map positions until no significant QTLs was detected. Final QTL model was built based on QTLs with LOD value comprised between 2 and 3 ("suggestive") and QTLs with LOD ≥ 3 ("putative").

A QTL was considered as “major” when the *R*^2^ > 10% in RIL populations and *R*^2^ > 5% in GWAS.

Confidence intervals were calculated according to the Darvasi's formula for RILs; CI = 163/(N × *R*^2^), where N is the number of RILs and *R*^2^ is the determination coefficient of each QTL.

**Supplementary Text S2. Genome-wide association (GWAS) analysis in the Unibo-DP durum panel. Methodology.**

After filtering for minor allele frequency ≥ 0.05 (corresponding to ten or fewer accessions) markers were used for the GWAS test in TASSEL software, v5.0 (Bradbury et al., 2007), based on a mixed linear model including populations structure covariate-(Q) and kinship matrix (K) for relationships among accessions (see Canè et al., 2014). LD decay rate among SNP and DArT® markers was assessed in Haploview (Barrett et al., 2005). The Haploview Tagger function (based on analysis of marker pairwise r2 values) was used to estimate the number of unique non-redundant bi-allelic markers (Tagger filter set at r2 = 1.00), which was equal to 5,108 and the number of independent tests (Tagger filter set at r2 = 0.3, Carlson et al., 2004), equal to 771. The latter was used to define an experiment-wise significance threshold based on the Bonferroni adjustment for multiple GWAS tests (P ≤ 0.05), which was equal to -LOG10 P = 4.19 (rounded to 4). Since the density of mapped SNPs exceeded the average genetic distance for LD decay to r2 = 0.3 (2.2 cM), GWAS-QTLs were detected as multiple marker-trait associations (MTA) of SNPs in LD. Significance intervals of GWAS-QTLs were reported as the intervals including all SNPs associated to the trait (P ≤ 0.01 experiment-wise) and in LD (r2 ≥ 0.03). Confidence intervals were defined based on the GWAS peak ± 2.2 cM on both map sides.

Three levels of significance were considered for reporting the GWAS-QTLs: (i) marker-wise P ≤ 0.01, (-LOG10 P ≥ 2.00) for "suggestive QTLs", (ii) marker-wise P ≤ 0.001 (-LOG10 P ≥ 3.00) for "nominal QTLs", (iii) experiment-wise P ≤ 0.05 (-LOG10 P ≥ 4.00), for "major QTLs". Since the density of mapped SNPs exceeded the average genetic distance for LD decay to r2 = 0.3 (2.2 cM), GWAS-QTLs were detected as multiple marker-trait associations (MTA) of SNPs in LD. Significance intervals of GWAS-QTLs were reported as the intervals including all SNPs associated to the trait (P ≤ 0.01 experiment-wise) and in LD (r2 ≥ 0.03). Confidence intervals were defined based on the GWAS peak ± 2.4 cM on both map sides (Maccaferri et al. 2014).

**References:**

Barrett JC, Fry B, Maller J, Daly MJ. 2005. Haploview: analysis and visualization of LD and haplotype maps. Bioinformatics 21, 263-265.

Bradbury PJ, Zhang Z, Kroon DE, Casstevens TM, Ramdoss Y, Buckler ES. 2007. TASSEL: software for association mapping of complex traits in diverse samples. Bioinformatics 23, 2633-2635.

Carlson CS, Eberle MA, Rieder MJ, Yi Q, Kruglyak L, Nickerson DA. 2004. Selecting a maximally informative set of single-nucleotide polymorphisms for association analyses using linkage disequilibrium. The American Journal of Human Genetics 74,106-120.

Kao CH, Zeng ZB, Teasdale RD. 1999. Multiple interval mapping for quantitative trait loci. Genetics 152: 1203-1216.

Maccaferri M, Cane' MA, Sanguineti MC, Salvi S, Colalongo MC, Massi A, Clarke F, Knox R, Pozniak CJ, Clarke JM, Fahima T, Dubcovsky J, Xu S, Ammar K, Karsai I, Vida G, Tuberosa R. 2014. A consensus framework map of durum wheat (Triticum durum Desf.) suitable for linkage disequilibrium analysis and genome-wide association mapping. BMC Genomics. 2014 15:873.

Zeng ZB, Kao CH, Basten CJ. 1999. Estimating the genetic architecture of quantitative traits. Genetics Research 74, 279-289.

**Supplementary Table S1. Correlation coefficients among root system architecture measurements for the Colosseo×Lloyd and Meridiano×Claudio recombinant inbred line populations as assessed at seedling stage.**

| **Genotype** | **iGWT** | **RGA** | **R6th** | **LRN** | **TRN** | **SL** | **SDW** | **RDW** | **RSR** | **PRL** | **TRL** |
| --- | --- | --- | --- | --- | --- | --- | --- | --- | --- | --- | --- |
| Colosseo × Lloyd population | | | | | | | | | | | |
| RGA | 0.09 | 1 |  |  |  |  |  |  |  |  |  |
| R6th | -0.23 | -0.10 | 1 |  |  |  |  |  |  |  |  |
| LRN | 0.18 | 0.02 | -0.05 | 1 |  |  |  |  |  |  |  |
| TRN | -0.18 | 0.02 | 0.93 | 0.01 | 1 |  |  |  |  |  |  |
| SL | -0.19 | -0.04 | 0.19 | 0.08 | 0.27 | 1 |  |  |  |  |  |
| SDW | -0.29 | -0.09 | 0.24 | 0.04 | 0.31 | 0.52 | 1 |  |  |  |  |
| RDW | -0.08 | -0.11 | 0.31 | 0.12 | 0.36 | 0.33 | 0.68 | 1 |  |  |  |
| RSR | 0.25 | -0.02 | 0.08 | 0.10 | 0.08 | -0.22 | -0.34 | 0.46 | 1 |  |  |
| PRL | 0.10 | -0.06 | -0.01 | 0.25 | 0.01 | 0.03 | 0.17 | 0.23 | 0.08 | 1 |  |
| TRL | 0.15 | -0.09 | 0.27 | 0.24 | 0.33 | 0.11 | 0.25 | 0.39 | 0.19 | 0.86 | 1 |
| ARL | 0.16 | -0.20 | -0.06 | 0.24 | -0.02 | 0.03 | 0.20 | 0.30 | 0.14 | 0.91 | 0.92 |
| Meridiano × Claudio population | | | | | | | | | | | |
| RGA | -0.12 | 1 |  |  |  |  |  |  |  |  |  |
| R6th | -0.14 | 0.07 | 1 |  |  |  |  |  |  |  |  |
| LRN | 0.13 | 0.05 | 0.03 | 1 |  |  |  |  |  |  |  |
| TRN | -0.13 | 0.12 | 0.71 | 0.01 | 1 |  |  |  |  |  |  |
| SL | -0.24 | 0.08 | 0.17 | 0.05 | 0.21 | 1 |  |  |  |  |  |
| SDW | -0.08 | 0.03 | 0.11 | 0.03 | 0.14 | 0.70 | 1 |  |  |  |  |
| RDW | -0.35 | 0.06 | 0.12 | 0.06 | 0.30 | 0.39 | 0.51 | 1 |  |  |  |
| RSR | -0.26 | 0.03 | 0.01 | 0.02 | 0.15 | - 0.33 | -0.52 | 0.47 | 1 |  |  |
| PRL | -0.45 | 0.04 | 0.20 | 0.12 | 0.15 | 0.36 | 0.30 | 0.47 | 0.15 | 1 |  |
| TRL | -0.36 | -0.03 | 0.42 | 0.25 | 0.59 | 0.38 | 0.36 | 0.58 | 0.19 | 0.75 | 1 |
| ARL | -0.15 | 0.01 | -0.02 | 0.10 | 0.01 | 0.03 | 0.15 | 0.56 | 0.12 | 0.80 | 0.85 |

*P* ≤ 0.001

*P* ≤ 0.01

*P* ≤ 0.05

Significance:

**Supplementary Text S3. Phenotypic correlations between seminal root and shoot traits.**

iTGW showed weak though significant correlations with both shoot and root seminal traits (*r* = -0.45 for PRL, -0.35 for RDW and -0.29 for SDW). As to iTGW, the only significant correlations were detected in Mr×Cl (*r* = -0.45 with PRL and -0.36 with TRL).

Among root traits, RGA showed no correlation with any other root and shoot trait, while most other root traits were positively associated with SL and SDW (*r* values from 0.25 to 0.35).

In both populations, presence of lateral roots in the primary seminal root correlated weekly and positively with PRL, TRL and iTGW (*r* from 0.12 to 0.25). A strong correlation was found between RDW and SDW (*r* = 0.68 and 0.51 for Co×Ld and Mr×Cd populations, respectively). Root diameter, surface and volume of primary roots were all correlated with each other and also with root length and root number at *r* > 0.60 (data not reported). SL was correlated with SDW (*r* = 0.52 and 0.70 in Co×Ld and Mr×Cd, respectively) and, to a lesser extent, with RDW (*r* = 0.33 and 0.39, respectively).

**Supplementary Table S2. QTLs detected for root system architecture (RSA) traits detected in the Colosseo×Lloyd mapping population at the seedling stage. QTLs are grouped in QTL clusters.**

| **Trait ^1^** | **QTL** | **Marker** | **Confidence interval ^2^**  **(cM)** | **LOD**  **(unit)** | **Additive effect ^3^**  **(homozygous allelic subst.)** | **Additive effect ^4^**  **(%)** | ***R*^2^**  **(%)** | **Projected Confidence**  **interval ^5^**  **(cM)** | |  |
| --- | --- | --- | --- | --- | --- | --- | --- | --- | --- | --- |
| TRL | *QTrl.ubo-1B* | *IWB30286* | 0-4-11 | 3.24 | -7.70 | 7.8 | 6.6 | | 6-14-20 | |
| TRV | *QTrv.ubo-1B* | *IWB12258* | 0-2-11 | 2.90 | -0.04 | 18.1 | 5.1 | | 6-9-21 | |
| SL | *QSl. ubo-1B* | *KBO0203* | 87-89-92 | 11.74 | -1.06 | 7.7 | 19.5 | | 95-96-101 | |
| LRN | *QLrn. ubo-2A* | *IWB9477* | 0-6-15 | 2.50 | 1.68 | 17.7 | 4.9 | | 0-14-24 | |
| TRL | *QTrl. ubo-2B.1* | *wPt-7970* | 3-8-13 | 4.92 | -9.00 | 9.1 | 9.8 | | 2-7-16 | |
| ARL | *QArl. ubo-2B.1* | *wPt-7970* | 2-8-14 | 3.44 | -1.58 | 8.5 | 7.9 | | 0-8-19 | |
| PRL | *QPrl. ubo-2B.1* | *wPt-7970* | 2-8-15 | 3.05 | -1.42 | 6.2 | 7.3 | | 0-8-19 | |
| TRS | *QTrs. ubo-2B.1* | *wPt-7970* | 1-8-16 | 2.85 | -1.76 | 11.4 | 6.5 | | 0-8-23 | |
| RGA | *QRga. ubo-2B.1a* | *IWB4604* | 32-36-40 | 6.21 | -9.96 | 12.2 | 11.7 | | 37-43-45 | |
| RGA | *QRga. ubo-2B.1b* | *IWB39220* | 57-65-72 | 2.10 | 5.96 | 7.3 | 6.2 | | 69-77-87 | |
| RDW | *QRdw. ubo-2B.1* | *IWB62688* | 42-53-65 | 2.58 | -0.92 | 6.9 | 4.0 | | 46-67-77 | |
| RGA | *QRga. ubo-2B.2* | *IWB62718* | 0-3-14 | 2.00 | 4.98 | 6.1 | 4.4 | | 100-114-130 | |
| TRN | *QTrn. ubo-2B.2* | *wPt-3651* | 18-23-27 | 6.31 | 0.24 | 4.5 | 11.1 | | 138-145-150 | |
| ARL | *QArl. ubo-2B.2* | *wPt-3651* | 15-21-27 | 3.46 | -1.38 | 7.4 | 7.8 | | 132-141-150 | |
| RT6 | *QRt6. ubo-2B.2* | *wPt-3651* | 16-22-27 | 5.83 | 17.58 | 48.4 | 8.8 | | 134-143-150 | |
| PRL | *QPrl. ubo-2B.2* | *wPt-3042* | 8-17-21 | 4.04 | -1.68 | 7.3 | 7.0 | | 132-141-152 | |
| PRD | *QPrd. ubo-2B.2* | *wPt-3042* | 6-17-23 | 2.54 | -0.002 | 4.6 | 5.4 | | 130-141-156 | |
| TRD | *QTrd. ubo-3A.2* | *CFA2234* | 7-16-24 | 2.78 | 0.02 | 8.6 | 5.3 | | 50-64-75 | |
| RT6 | *QRt6. ubo-3A.2* | IWB72529 | 10-17-24 | 3.62 | 14.02 | 38.6 | 6.9 | | 55-67-75 | |
| TRN | *QTrn. ubo-3A.2* | *IWB11027* | 30-35-40 | 4.89 | 0.22 | 4.6 | 9.2 | | 81-87-94 | |
| RDW | *QRdw. ubo-3A.2* | *IWB72058* | 90-93-96 | 3.97 | 1.62 | 12.2 | 16.0 | | 137-139-141 | |
| SDW | *QSdw. ubo-3A.2* | *BARC51* | 107-116-125 | 2.53 | 0.92 | 6.6 | 5.2 | | 146-156-169 | |
| TRN | *QTrn. ubo-3A.2* | *IWB58806* | 103-111-118 | 3.13 | 0.18 | 3.4 | 6.2 | | 144-148-162 | |
| RT6 | *QRt6. ubo-3A.2* | *IWB58806* | 103-111-118 | 3.34 | 13.7 | 37.7 | 6.0 | | 144-148-163 | |
| TGW | *QTgw. ubo-3A.2* | *IWB58806* | 106-111-115 | 3.92 | -3.14 | 6.2 | 9.6 | | 146-148-155 | |
| LRN | *QLrn. ubo-3A.2* | *IWB63935* | 126-135-144 | 2.52 | 1.76 | 18.5 | 5.2 | | 170-184-194 | |
| TRD | *QTrd. ubo-3B* | *IWB10030* | 122-130-137 | 3.03 | -0.02 | 8.6 | 6.6 | | 158-165-172 | |
| TRV | *QTrv. ubo-4A.2* | *KBO0058* | 44-54-64 | 2.67 | -0.04 | 18.1 | 4.6 | | 54-66-77 | |
| RDW | *QRdw. ubo-4B* | *GWM165* | 50-56-63 | 2.92 | -0.96 | 7.2 | 6.9 | | 59-62-68 | |
| RSR | *QRsr. ubo-4B* | *GWM165* | 53-56-60 | 3.45 | -0.24 | 25 | 14.7 | | 60-62-65 | |
| RT6 | *QRt6. ubo-4B* | *IWB154* | 63-71-80 | 2.58 | -12.06 | 33.2 | 5.3 | | 68-74-85 | |
| RGA | *QRga.ubo-4B* | *IWB60481* | 60-72-84 | 2.12 | 4.78 | 5.8 | 4.0 | | 66-75-91 | |
| TRN | *QTrn. ubo-4B* | *GWM6* | 80-89-99 | 2.9 | -0.16 | 3.0 | 5.0 | | 85-93-99 | |
| TRN | *QTrn. ubo-5A.1* | *IWB47433* | 43-47-50 | 8.09 | 0.26 | 4.9 | 13.7 | | 45-48-50 | |
| PRL | *QPrl. ubo-5A.1* | *IWB66553* | 29-37-46 | 3.2 | -1.56 | 6.8 | 5.3 | | 31-40-47 | |
| RT6 | *QRt6. ubo-5A.1* | *IWB47433* | 44-47-49 | 10.27 | 23.36 | 64.3 | 16.6 | | 46-48-50 | |
| SL | *QSl. ubo-5A.2* | *CFD391* | 37-50-63 | 2.51 | -0.44 | 3.2 | 3.6 | | 153-153-173 | |
| SL | *QSl.6 ubo-6A* | *IWA6724* | 65-72-80 | 3.58 | -0.56 | 4.1 | 6.4 | | 62-69-77 | |
| TRL | *QTrl. ubo-6A* | *IWA441* | 118-125-132 | 3.94 | -8.02 | 8.1 | 6.8 | | 116-122-128 | |
| RGA | *QRga. ubo-6A* | *IWA441* | 122-125-128 | 9.11 | 10.02 | 12.2 | 17.8 | | 119-122-123 | |
| ARL | *QArl. ubo-6A* | *IWA441* | 117-125-133 | 2.84 | -1.24 | 6.6 | 5.6 | | 115-122-129 | |
| SL | *QSl. ubo-6B* | *IWB10698* | 76-78-80 | 5.49 | 1.42 | 10.3 | 20.3 | | 77-79-81 | |
| TRD | *QTrd. ubo-6B* | *IWB49257* | 84-90-97 | 3.39 | 0.02 | 8.6 | 7.0 | | 85-93-100 | |
| RGA | *QRga. ubo-7A* | *wPt-9207* | 8-16-25 | 3.55 | 6.00 | 7.3 | 5.5 | | 0-10-20 | |
| SDW | *QSdw. ubo-7B* | *GWM573* | 51-57-63 | 3.59 | -1.12 | 8 | 8.0 | | 46-52-58 | |
| TRL | *QTrl. ubo-7B* | *UBW26* | 194-203-212 | 2.56 | 6.46 | 6.5 | 5.2 | | 198-209-215 | |
| TRS | *QTrs. ubo-7B* | *UBW26* | 195-203-211 | 2.82 | -1.76 | 11.3 | 5.9 | | 199-209-214 | |
| ARL | *QArl. ubo-7B* | *wPt-6865* | 199-209-219 | 2.65 | 1.18 | 6.3 | 4.7 | | 205-212-222 | |

**^1^** LRN; Lateral root number, RT6; % plants with root6th, RDW; Root dry weight (mg/seedling), RSR; Root to shoot ratio, SDW; Shoot dry weight (mg/seedling); SL, Shoot lenght (cm); RGA, seminal root angle (°); TRN, total root number; TGW, grain weight measured as 1,000 grain weight; ARL, Average root length; MRL, Maximum root length; PRD, Primary root diameter; PRL, Primary root length; TRD, Total root diameter; TRL, Total root length; TRS, Total root surface; TRV, Total root volume.

**^2^** Upper- and lower QTL confidence interval positions. The central value reports the QTL peak location

**^3^** Additive effect calculated as homozygous allelic substitution (double dose allelic substitution) = Average trait value of Colosseo’s allele – average trait value of Lloyd’s allele

**^4^** Percent phenotypic variation explained by the QTL.

^5^ QTL confidence intervals and QTL peaks projected on the tetraploid consensus map (Maccaferri et al. 2015).

**Supplementary Table S3.** QTLs detected for root system architecture traits in the Meridiano×Claudio mapping population. QTLs are grouped in QTL clusters.

| **Trait ^1^** | **QTL** | **Marker** | **Confidence interval ^2^**  **(cM)** | **LOD**  **(unit)** | **Additive**  **effect ^3^**  **(homozygous allelic subst.)** | **Additive effect ^4^**  **(%)** | ***R*^2^**  **(%)** | **Projected confidence**  **interval ^5^**  **(cM)** |
| --- | --- | --- | --- | --- | --- | --- | --- | --- |
| PRV | *QPrv.ubo-1A* | *wPt-4676* | 0-0-9 | 2.30 | 0.01 | 3.5 | 5.13 | 1-2-6 |
| TRV | *QTrv.ubo-1A* | *wPt-4676* | 0-0-12 | 1.83 | 0.01 | 4 | 3.73 | 1-2-11 |
| RDW | *QRdw.ubo-1A2* | *wPt-731018* | 0-6-17 | 1.79 | -0.39 | -3.3 | 4.35 | 45-51-63 |
| RDW | *QRdw.ubo-1A2* | *wmc304* | 45-50-54 | 2.61 | 0.62 | 5.3 | 10.9 | 98-101-104 |
| TRS | *QTrs.ubo-1B* | *tPt-5413* | 11-18-24 | 3.56 | -0.21 | -3.7 | 7.18 | 12-16-17 |
| TRV | *QTrv.ubo-1B* | *tPt-5413* | 14-20-25 | 3.67 | -0.01 | -6 | 8.90 | 16-17-17 |
| TRL | *QTrl.ubo-1B* | *wPt-733882* | 46-50-54 | 3.72 | -5.10 | -4.2 | 10.78 | 35-42-50 |
| ARL | *QArl. ubo-1B* | *wPt-733882* | 51-55-59 | 3.97 | 0.00 | -3.5 | 9.82 | 43-51-57 |
| TRN | *QTrn.ubo-2A* | *IWB12312* | 7-17-26 | 1.76 | 0.08 | 1.7 | 4.79 | 10-15-18 |
| RT6 | *QRt6.ubo-2A1* | *IWB72948* | 23-34-45 | 3.27 | 4.24 | 26.5 | 4.15 | 17-26-39 |
| LRN | *QLrn.ubo-2A2* | *wPt-669355* | 17-26-36 | 2.49 | -1.58 | -15.2 | 4.77 | 176-206-207 |
| TRV | *QTrv.ubo-2A* | *wmc658* | 13 -26-38 | 1.86 | 0.01 | 4 | 3.76 | 197-210-211 |
| TGW | *QTgw.ubo-2A2* | *wPt-2696* | 19-27-38 | 2.72 | 1.60 | 3.3 | 3.51 | 200-212-226 |
| TRN | *QTrn.ubo-2B* | *wmc265* | 92-99-106 | 3.56 | 0.10 | 2.0 | 6.73 | 89-94-103 |
| PRL | *QPrl.ubo-2B* | *wPt-4300a* | 113-125-138 | 1.88 | -0.61 | -2.0 | 3.70 | 110-123-134 |
| TRS | *QTrs.ubo-2B* | *wmc441* | 121-124-127 | 5.04 | -0.31 | -5.5 | 15.29 | 119-123-125 |
| ARL | *QArl. ubo-2B* | *wPt-2430* | 132-139-146 | 3.38 | -0.66 | -1.3 | 6.69 | 128-133-138 |
| RDW | *QRdw.ubo-2B* | *IWB1384* | 183-190-198 | 1.63 | 0.46 | 4 | 6.15 | 167-170-173 |
| SL | *QSl.ubo-2B* | *IWB1384* | 189-198-207 | 2.16 | 0.40 | 2.9 | 5.32 | 170-173-175 |
| RGA | *QRga.ubo-2B* | *IWB13829 = IWB13830* | 202-209-216 | 3.11 | 5.72 | 5.8 | 6.72 | 175-177-183 |
| TRD | *QTrd.ubo-3A1* | *barc57* | 0-2-9 | 2.85 | 0.01 | 3.5 | 7.01 | 0-1-7 |
| PRD | *QPrd.ubo-3A* | *barc57* | 0-0-10 | 1.86 | 0.01 | 3 | 4.75 | 0-0-8 |
| SL | *QSl.ubo-3A2* | *IWB71974* | 22-27-32 | 5.29 | -0.55 | -4.1 | 9.88 | 34-37-38 |
| TRN | *QTrn.ubo-3A* | *IWB69167* | 26-42-57 | 1.52 | -0.07 | -1.3 | 2.97 | 170-183-184 |
| PRS | *QPrs.ubo-3B* | *wPt-744251* | 5-16-26 | 1.69 | 0.21 | 2.8 | 4.31 | 3-8-12 |
| TRD | *QTrd.ubo-3B1* | *wPt-744251* | 22-30-37 | 1.84 | 0.01 | 3.3 | 6.02 | 10-14-17 |
| TRV | *QTrv.ubo-3B1* | *IWB39127* | 38-55-71 | 1.31 | 0.01 | 3.4 | 2.83 | 17-27-40 |
| ARL | *QArl. ubo-3B1* | *IWB40465* | 97-105-113 | 2.80 | 0.61 | 0 | 5.70 | 69-75-85 |
| TRL | *QTrl.ubo-3B1* | *IWB72454* | 100-112-124 | 1.85 | 3.08 | 2.5 | 3.93 | 72-84-93 |
| TGW | *QGwt.ubo-3B1* | *wPt-2698b* | 7-15-31 | 4.69 | -2.20 | -4.6 | 6.52 | 148-158-167 |
| RGA | *QRga.ubo-3B2* | *wPt-5947* | 41-52-63 | 2.23 | -4.54 | -4.6 | 4.23 | 181-188-196 |
| LRN | *QLrn.ubo-3B* | *wPt-9488* | 61-73-85 | 1.92 | -1.40 | -13.5 | 3.75 | 189-202-206 |
| TRD | *QTrd.ubo-4B* | *IWB74227* | 0-0-6 | 1.76 | 0.01 | 3.5 | 7.13 | 2-3-9 |
| SDW | *QSdw.ubo-4B* | *IWB55598* | 1-6-11 | 2.71 | 0.63 | 5.2 | 9.93 | 5-10-16 |
| LRN | *QLrn.ubo-4B.1* | *IWA4662* | 11-23-30 | 2.19 | 2.70 | 26.0 | 15.26 | 11-22-21 |
| SL | *QSl.ubo-4B* | *IWB72442* | 22-25-26 | 6.72 | 0.77 | 5.6 | 19.51 | 21-22-24 |
| LRN | *QLrn.ubo-4B.2* | *gwm1278* | 39-41-43 | 6.62 | -3.76 | -36.2 | 26.85 | 23-33-35 |
| TGW | *QTgw.ubo-4B* | *wmc89* | 50-59-70 | 9.37 | -4.20 | -8.7 | 12.5 | 31-42-51 |
| PRV | *QPrv.ubo-4B* | *barc340* | 50 -64-78 | 1.51 | 0.01 | 2.8 | 3.28 | 41-56-66 |
| PRD | *QPrd.ubo-4B* | *gwm513* | 53-63-73 | 2.03 | 0.01 | 3 | 4.67 | 45-57-65 |
| RT6 | *QRt6. ubo-4B* | *IWB9672* | 75-79-83 | 7.19 | 7.06 | 44.1 | 11.23 | 66-66-70 |
| TRN | *QTrn.ubo-4B* | *kbo0170* | 79-83-86 | 7.22 | 0.15 | 2.9 | 14.86 | 69-75-81 |
| PRL | *QPrl.ubo-4B* | *IWB28273* | 85-95-105 | 2.45 | 0.68 | 2.2 | 4.61 | 75-82-87 |
| PRS | *QPrs.ubo-4B* | *IWB28273* | 87-95-102 | 2.90 | 0.25 | 3.3 | 6.33 | 76-82-86 |
| TRL | *QTrl.ubo-4B* | *Kbo0236* | 93-97-101 | 6.10 | 5.50 | 4.5 | 12.61 | 80-82-89 |
| RGA | *QRga.ubo-4B* | *IWB12276* | 95-99-102 | 6.46 | -8.20 | -8.4 | 13.81 | 82-83-86 |
| TRV | *QTrv.ubo-4B* | *wPt-663949* | 100-113-126 | 1.69 | 0.01 | 3.8 | 3.49 | 84-93-108 |
| TRS | *QTrs.ubo-4B* | *gwm6* | 107-115-123 | 3.03 | 0.19 | 3.4 | 5.95 | 89-94-105 |
| LRN | *QLrn.ubo-5A2* | *IWB10594* | 10-16-23 | 2.86 | -1.94 | -18.7 | 6.98 | 110-116-126 |
| SDW | *QSdw.ubo-5A2* | *wPt-730410* | 27-32-38 | 3.53 | -0.58 | -4.7 | 8.32 | 128-135-140 |
| RGA | *QRga.ubo-5B1* | *IWB75279* | 0-0-8 | 3.19 | -5.50 | -5.6 | 6.14 | 2-2-13 |
| ARL | *QArl. ubo-5B1* | *IWB7137* | 0-3-12 | 2.47 | 0.60 | -24.0 | 5.30 | 2-7-17 |
| SDW | *QSdw.ubo-5B2* | *IWB2610* | 0-0-6 | 2.61 | 0.60 | 4.6 | 7.68 | 34-34-39 |
| SL | *QSl.ubo-5B4* | *IWB71624* | 50-59-68 | 2.61 | 0.40 | 2.9 | 5.12 | 129-136-142 |
| RDW | *QRdw.ubo-6A1* | *IWB64435* | 2-10-18 | 2.41 | -0.46 | -3.9 | 5.88 | 0-4-11 |
| TGW | *QTgw.ubo-6A1* | *IWB66509* | 0-8-21 | 2.73 | 1.60 | 3.3 | 3.44 | 0-0-10 |
| RT6 | *QRt6. ubo-6A2* | *IWB38287* | 0-7-23 | 5.19 | 3.56 | 22.2 | 2.94 | 33-43-61 |
| TRL | *QTrl.ubo-6A2* | *IWB50538* | 62-72-83 | 1.78 | 3.22 | 2.6 | 4.36 | 104-113-126 |
| TRS | *QTrs.ubo-6A2* | *wPt-732741* | 74-85-97 | 2.14 | 0.16 | 2.8 | 3.99 | 116-127-134 |
| PRV | *QPrv.ubo-6A* | *wPt-732741* | 73-85-97 | 1.78 | 0.07 | 3.1 | 3.92 | 115-127-134 |
| PRL | *QPrl.ubo-6A* | *wPt-732328* | 77-83-90 | 3.77 | 0.84 | 2.7 | 7.07 | 123-126-129 |
| PRS | *QPrs.ubo-6A2* | *IWB72197* | 78-83-88 | 4.31 | 0.30 | 4.0 | 9.28 | 123-126-128 |
| RGA | *QRga.ubo-6A2* | *IWB69393* | 85-92-98 | 2.94 | -5.84 | -5.9 | 6.98 | 127-130-136 |
| LRN | *QLnr.ubo-6B* | *wPt-1437* | 39-46-53 | 2.46 | -1.86 | -17.9 | 6.40 | 15-22-30 |
| RT6 | *QRt6. ubo-6B* | *IWA8011* | 116.9-123-129 | 5.22 | 6.04 | 37.8 | 8.10 | 72-76-80 |
| ARL | *QArl. ubo-6B* | *IWB8011* | 122.7-129-135 | 2.89 | -0.72 | -3.1 | 7.67 | 76-80-85 |
| PRS | *QPrs.ubo-6B* | *gpw3241* | 134-143-153 | 2.14 | -0.22 | -2.9 | 4.72 | 84-91-103 |
| PRL | *QPrl.ubo-6B* | *gpw3241* | 138-143-148 | 3.53 | -1.00 | -3.2 | 9.34 | 86-90-95 |
| PRL | *QPrl.ubo-6B* | *barc178* | 161-167-176 | 2.54 | 0.81 | 2.6 | 6.44 | 108-113-122 |
| TRN | *QTrn.ubo-6B* | *gwm219* | 161-170-179 | 2.77 | 0.90 | 1.7 | 5.35 | 108-117-122 |
| SDW | *QSdw.ubo-7A1* | *IWB73134* | 0-9-20 | 1.97 | 0.42 | 3.4 | 4.28 | 0-7-22 |
| TRS | *QTrs.ubo-7A 2* | *IWB73246* | 0-6-11 | 4.51 | 0.24 | 4.2 | 9.34 | 99-113-117 |
| PRV | *QPrv.ubo-7A 2* | *IWB73246* | 0-6-13 | 2.62 | 0.01 | 3.9 | 6.17 | 99-113-119 |
| PRD | *QPrd.ubo-7A2* | *IWB73246* | 1-6-11 | 3.64 | 0.01 | 4.3 | 9.59 | 102-114-118 |
| RGA | *QRga.ubo-7A2* | *IWB35428* | 22-28-36 | 2.63 | 5.62 | 5.7 | 6.46 | 125-130-135 |
| SL | *QSl.ubo-7A2* | *IWB10968* | 50-48-51 | 5.89 | -0.64 | -4.7 | 13.55 | 151-147-154 |
| SL | *QSl.ubo-7A2* | *wPt-3782* | 89-97-105 | 2.99 | 0.41 | 3.0 | 5.59 | 183-191-198 |
| TRD | *QTrd.ubo-7B* | *IWB73246* | 2-6-9 | 5.86 | 0.01 | 4.7 | 13.67 | 0-6-16 |
| RT6 | *QRt6. ubo-7B* | *IWB73338* | 64-81-100 | 2.96 | -3.40 | -21.3 | 2.62 | 69-93-109 |
| PRS | *QPrs.ubo-7B* | *wPt-5646* | 196-203-211 | 3.03 | 0.26 | 3.4 | 6.54 | 169-179-182 |
| PRL | *QPrl.ubo-7B* | *wPt-4300b* | 197-202-208 | 3.89 | 0.95 | 3.1 | 8.50 | 170-178-181 |

**^1^** LRN; Lateral root number, RT6; % plants with root6th, RDW; Root dry weight (mg/seedling), RSR; Root to shoot ratio, SDW; Shoot dry weight (mg/seedling); SL, Shoot lenght (cm); RGA, seminal root angle (°); TRN, total root number; TGW, grain weight measured as 1,000 grain weight; ARL, Average root length; MRL, Maximum root length; PRD, Primary root diameter; PRL, Primary root length; TRD, Total root diameter; TRL, Total root length; TRS, Total root surface; TRV, Total root volume.

**^2^** Upper- and lower QTL confidence interval positions. The central value reports the QTL peak location

**^3^** Additive effect calculated as homozygous allelic substitution (double dose allelic substitution) = Average trait value of Meridiano’s allele – average trait value of Claudio’s allele

**^4^** Percent phenotypic variation explained by the QTL.

^5^ QTL-confidence intervals and QTL-peaks projected on the tetraploid consensus map (Maccaferri et al. 2015).
